# Fine-tuning of stable organic free-radical photosensitizers for photodynamic immunotherapy†

**DOI:** 10.1039/d3sc06826a

**Published:** 2024-04-03

**Authors:** Xiang Wang, Gaona Shi, Rao Wei, Meng Li, Qingyang Zhang, Tiantai Zhang, Chuan-Feng Chen, Hai-Yu Hu

**Affiliations:** a State Key Laboratory of Bioactive Substances and Function of Natural Medicine, Institute of Materia Medica, Chinese Academy of Medical Sciences, Peking Union Medical College Beijing 100050 China haiyu.hu@imm.ac.cn ttzhang@imm.ac.cn; b Beijing National Laboratory for Molecular Sciences, CAS Key Laboratory of Molecular Recognition and Function, Institute of Chemistry, Chinese Academy of Sciences Beijing 100190 China cchen@iccas.ac.cn

## Abstract

Photodynamic immunotherapy (PDI) is an innovative approach to cancer treatment that utilizes photodynamic therapy (PDT) and photosensitizers (PSs) to induce immunogenic cell death (ICD). However, currently most commonly used PSs have restricted capabilities to generate reactive oxygen species (ROS) *via* a type-II mechanism under hypoxic environments, which limits their effectiveness in PDI. To overcome this, we propose a novel approach for constructing oxygen independent PSs based on stable organic free-radical molecules. By fine-tuning the characteristics of tris(2,4,6-trichlorophenyl)-methyl (TTM) radicals through the incorporation of electron-donating moieties, we successfully found that TTMIndoOMe could produce substantial amounts of ROS even in hypoxic environments. *In vitro* experiments showed that TTMIndoOMe could effectively produce O_2_˙^−^, kill tumor cells and trigger ICD. Moreover, *in vivo* experiments also demonstrated that TTMIndoOMe could further trigger anti-tumor immune response and exhibit a superior therapeutic effect compared with PDT alone. Our study offers a promising approach towards the development of next-generation PSs functioning efficiently even under hypoxic conditions and also paves the way for the creation of more effective PSs for PDI.

## Abstract 2

We developed a novel strategy to enhance type I photodynamic therapy by using doublet radicals instead of radical intermediates, which effectively generated ROS, triggered anti-tumor immune response and superior photodynamic immunotherapy efficacy.

## Graphical abstract

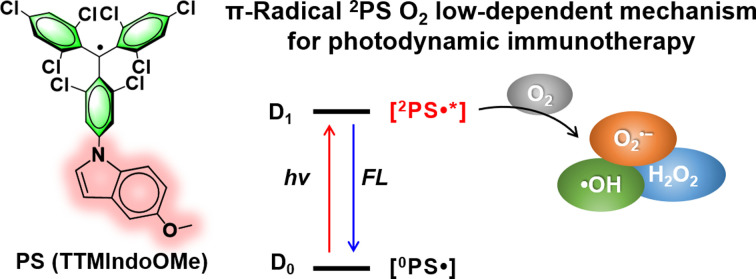

## Introduction

Photodynamic immunotherapy (PDI) has emerged as a promising therapeutic approach for combating cancer, which utilizes photodynamic therapy (PDT) to induce cancer cell death, thereby triggering innate and adaptive immune responses against the tumor.^1–10^ Photosensitizers (PSs) utilized in PDI generate reactive oxygen species (ROS) upon irradiation, which damages subcellular components, plasma membranes, and biomolecules, leading to oxidative stress-based cell death.^11–19^ Nevertheless, the efficacy of PDI is influenced by various factors, including the immunosuppressive tumor microenvironment, hypoxia-induced ROS resistance, and the limited availability of efficient PSs.^1–3^ Therefore, developing novel PSs with superior ROS-releasing efficacy, efficient induction of immunogenic cell death (ICD), and simple chemical structures is an urgent need for achieving effective PDI.

**Scheme 1:**
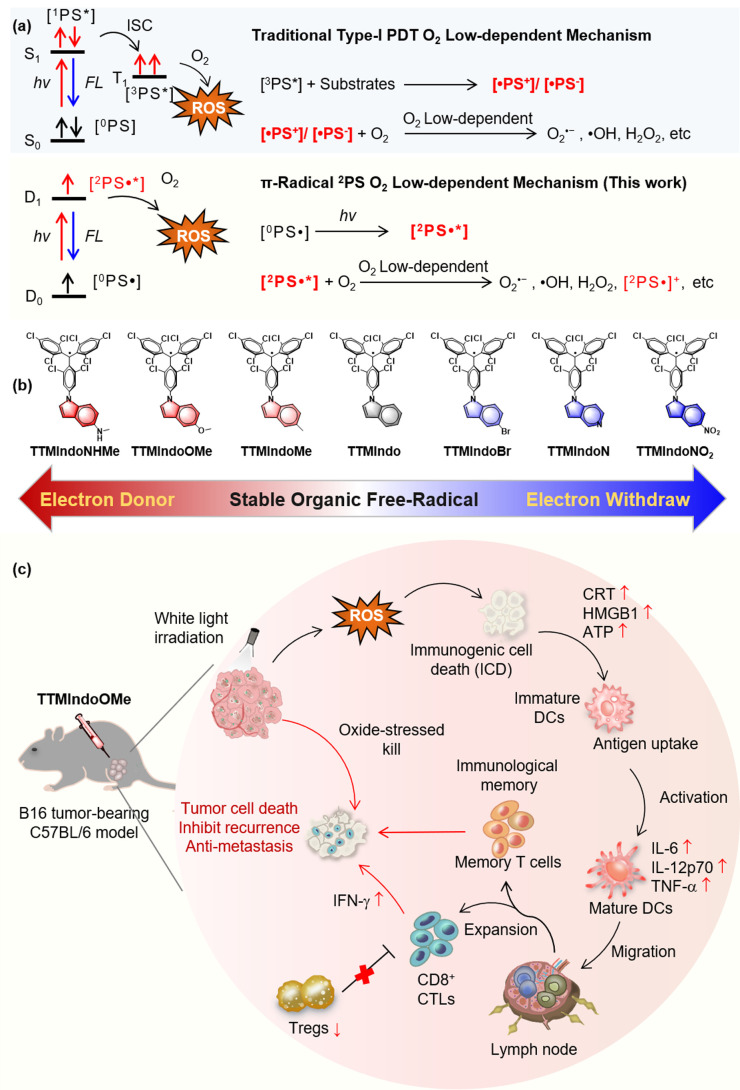
(a) The mechanism for the generation of oxidizing species. (b) The chemical structures of the TTMIndo derivatives. (c) Schematic mechanism of TTMIndoOMe for photodynamic immunotherapy of a B16 tumor-bearing C57BL/6 model *in vivo*.

The two mechanisms in PDT, type I and type II, refer to different ROS generation pathways.^14–21^ Specifically, type I PDT has an advantage over conventional type II PDT in the tumor microenvironment (TME) due to its lower oxygen dependence. In type I PDT, the PS is excited from the ground singlet state (S_0_) to the excited singlet state (S_*n*_), and then to the excited triplet state (T_*n*_) through intersystem crossing (ISC) upon photoactivation. The excited triplet excitons generate PS free radicals by exchanging electrons with triplet oxygen or biological substrates in the environment, which produce reactive oxygen radicals like superoxide anions (O_2_˙^–^) and hydroxyl radicals (˙OH) that kill tumor cells. The PS free radicals are crucial to the generation of ROS in PDT under hypoxia (Scheme 1a).^22–24^ However, most type I PSs are inorganic nanomaterials and heavy metal chelates that often exhibit high cytotoxicity without light irradiation. Moreover, they have low biocompatibility and are also non-biodegradable and expensive. Therefore, developing novel organic type I PSs for PDI would be highly beneficial.

Developing hypoxia-tolerant PSs for effective PDI in the hypoxic TME remains a significant and unresolved challenge. Stable organic radical molecules, characterized by their possession of unpaired electrons, exhibit remarkable photophysical properties that have found diverse applications in functional materials and photocatalysis.^25–32^ These molecules possess the unique ability to adopt doublet excited states upon irradiation.^33–35^ The unpaired electron narrows the energy gaps of the radicals, allowing red-shift for the absorption. The excited unpaired electron is able to react with O_2_ to produce ROS (O_2_˙^–^ and ˙OH) efficiently *via* an electron transfer mechanism, reducing dependence on oxygen during treatment.^30,36–41^ Nevertheless, despite their inherent advantages, the utilization of organic radical PSs in PDI has yet to be explored. Herein, we present a pioneering molecular design strategy wherein stable organic free radicals are employed as substitutes for conventional free radical intermediates [˙PS^+^]/[˙PS^−^] typically generated during the photochemical process. Our organic radical PS exhibited highly promising advantages, including (1) superior ROS generation under hypoxic environments triggered by white light, (2) effective induction of ICD in B16 tumor cells, (3) efficient improvement of proliferation and differentiation of CD8^+^ T cells and differentiation of T memory cells, and (4) efficient PDI in mice tumor models. This development opens new opportunities for future PDT and PDI research against cancer in the TME. To our knowledge, organic radical PSs for PDI have not been reported.

The type I mechanism of PDT was known for its inefficiency, primarily due to the limited formation of [˙PS^+^]/[˙PS^−^] intermediates resulting from restricted collisions between PSs and electron-rich substrates.^42,43^ In our study, we proposed a novel approach to address this limitation and enhance the generation of ROS under hypoxic conditions by substituting the [˙PS^+^]/[˙PS^−^] intermediates with doublet π-radicals (Scheme 1a). We hypothesized that upon light irradiation, these doublet π-radicals could efficiently transfer electrons to O_2_, resulting in the production of O_2_˙^−^. To validate our concept, we designed and synthesized a series of tris(2,4,6-trichlorophenyl)methyl (TTM) π-radicals using methods previously reported in the literature.^35,44^ TTM consists of three aryl groups surrounding the central methyl radical. The TTM radicals exhibit ambipolar properties due to their unique unpaired electron structures, enabling them to easily accept or release electrons. To our knowledge, stable organic free radicals have received limited attention as PSs in prior studies,^29^ and their potential application in PDI remains unexplored. Specifically, we employed TTM π-radicals as the core structure and incorporated the indole bulky π-group to bolster stability.^45–48^ Furthermore, we fine-tuned their ability to react with oxygen or biological substrates in the tumor microenvironment under irradiation by utilizing substituent groups with varying push–pull electron effects in the indole moiety. Scheme 1b illustrates the chemical structures of TTMIndo derivative compounds synthesized with ease on a milligram scale *via* nucleophilic reaction and oxidation. Detailed information regarding the synthetic procedure and structure characterization can be found in the ESI.† Notably, TTMIndoOMe exhibited substantial ROS generation upon irradiation under normoxic and hypoxic environments in HepG2 cells. Furthermore, TTMIndoOMe effectively induced ICD in B16 cells by triggering the release of damage-associated molecular pattern molecules (DAMPs), adenosine triphosphate (ATP), high mobility group box 1 (HMGB1), and calreticulin (CRT), thereby activating the immune system, promoting the secretion of pro-inflammatory cytokines, and specifically activating dendritic cells (DCs) (Scheme 1c). *In vivo* experiments further demonstrated that TTMIndoOMe induced ICD in the TME upon irradiation, enhanced the proliferation and differentiation of CD8^+^ T cells, and promoted the differentiation of T memory cells to inhibit tumor growth in a C57BL/6 mice tumor model. More importantly, TTMIndoOMe could induce the differentiation of T memory cells, which shows great potential in inhibiting tumor growth over a long period and thus opens new opportunities for future PDT and PDI research against cancer in the TME.

## Results and discussion

### Photophysical properties and theoretical study of TTM π-radical PSs

**Fig. 1:**
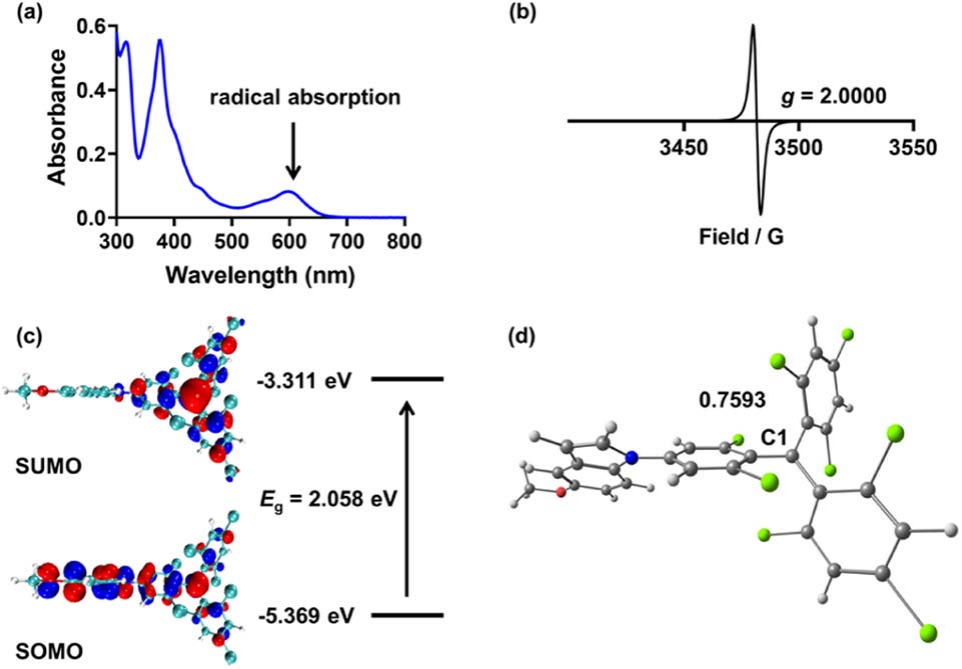
(a) The absorption spectrum of TTMIndoOMe in CHCl_3_ (10 μM). (b) The electron paramagnetic resonance spectrum of TTMIndoOMe in toluene (4 mM) at room temperature. (c) The frontier orbitals and energy gap of TTMIndoOMe calculated using DFT methods (B3LYP/6-31G(d)). (d) The Mülliken spin density distribution in C1 of TTMIndoOMe.

Firstly, we investigated the photophysical properties of the TTMIndo derivative compounds. The UV-Vis absorption spectra of these compounds in chloroform, PBS and water displayed two distinctive bands: a strong peak at 376 nm attributed to the localized transition from TTM and a weak absorption band around 610 nm (*ε* = 5000 L mol^−1^ cm^−1^) designated as the charge-transfer transition, both of which are indicative of radical absorption features (Fig. 1a and S1†). Next, we confirmed the presence of an unpaired electron in the radicals through electron paramagnetic resonance (EPR) measurements. The EPR spectra of the TTMIndo derivative compounds were examined in toluene, and the resulting spectra, double integrals, and spins of EPR signals (Fig. 1b and S2, Table S1†) provided clear evidence of the existence of an unpaired electron in the TTMIndo derivative compounds. These compounds exhibited EPR signals with a *g* factor (theoretical *g* = 2.0023 for a free electron) that was attributed to the TTM group, indicating the intrinsic presence of an unpaired electron in the molecule.

The energy levels of the frontier molecular orbitals (singly occupied molecular orbital (SOMO) and singly unoccupied molecular orbital (SUMO)) were determined through density functional theory (DFT) calculations using the Gaussian 16 program (Fig. 1c and S3†). The absorption spectra of TTMIndo derivative compounds exhibited a weak absorption band centred at approximately 610 nm, corresponding to an energy gap of 2.06 eV, which was assigned to the electronic transition from SOMO to SUMO. The strong, shorter-wavelength band in the absorption spectra was attributed to transitions from SOMO/HOMO to higher-energy orbitals. Additionally, energy level analysis showed that there was an intramolecular charge transfer (ICT) between the TTM radical and indole groups, corresponding to the weak absorption band in the absorption spectra of TTMIndo derivative compounds. Moreover, the calculated spin densities and Mülliken spin densities of the TTMIndo derivative compounds demonstrated a significant dependence on the substituent groups in the indole groups with different push–pull electron effects (Fig. 1d and S4†). There was a substantial increase in spin densities in the C1 of TTMIndo derivative compounds ascribed to the enhancement of the electron-withdrawing effect of the substituent groups in the indole group. The lower spin densities in C1 of TTMIndoNHMe, TTMIndoMe, and TTMIndoOMe indicated that the unpaired electron had a more delocalized π-system compared to TTMIndo derivative compounds with electron-withdrawing substituent groups in indole groups. The simulated calculations indicate enhanced stability of TTMIndo derivatives with electron-donating substituent groups due to a more delocalized π-system. This stability in a physiological system suggests their potential for bio-applications.

### Evaluation of ROS generation capability of TTM π-radical PSs

**Fig. 2:**
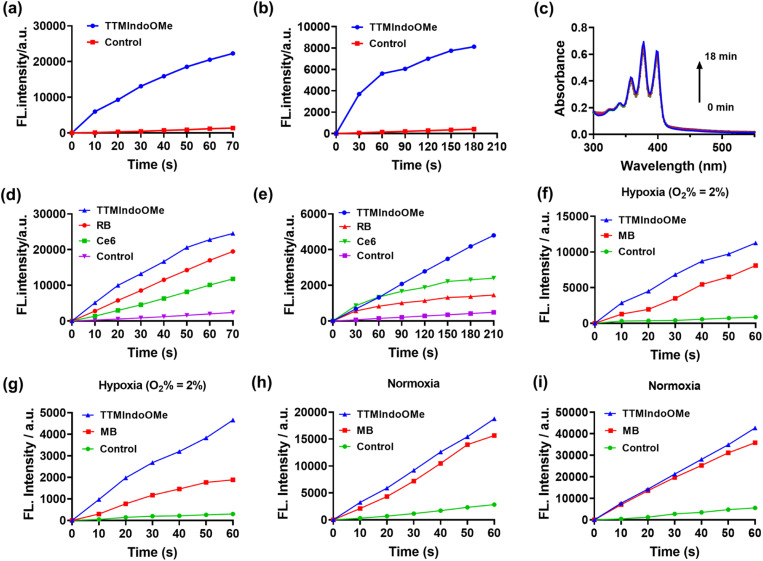
ROS (a) and O_2_˙^−^ (b) generation of TTMIndoOMe, and 2% DMSO as co-solvent in water upon irradiation with a white light source. (c) ^1^O_2_ generation of TTMIndoOMe in water upon irradiation with a white light source. ROS (d) and O_2_˙^–^ (e) generation of TTMIndoOMe, and commercial PSs Ce6 and RB in PBS upon irradiation with a white light source. ROS (f) and O_2_^·–^ (g) generation of TTMIndoOMe, and commercial PS methylene blue (MB) in PBS upon irradiation with a white light source under hypoxic (O_2_% = 2%) conditions. ROS (h) and O_2_^·–^ (i) generation of TTMIndoOMe, and commercial PS MB in PBS upon irradiation with a white light source under ambient conditions. DCFH as a ROS probe (40 μM, *λ*_ex_ = 480 nm, and *λ*_em_ = 525 nm), DHE as an O_2_^·–^ probe (20 μM, *λ*_ex_ = 525 nm, and *λ*_em_ = 625 nm), ABDA as an ^1^O_2_ probe (50 μM), and irradiation with a white LED light source (20 mW cm^−2^).

To evaluate the potential of TTMIndo derivatives as PSs for PDT, we investigated their ability to generate ROS in PBS upon 20 mW cm^−2^ white light LED irradiation, using a dichlorodihydrofluorescein (DCFH) probe which shows green emission in the presence of ROS. As depicted in Fig. 2a, S6, and S9a,†TTMIndo derivatives featuring electron-donating substituents within the indole groups, TTMIndoOMe and TTMIndoNHMe, demonstrated significant ROS generation, which is higher than that of TTMIndo. This was evidenced by a significant increase in the fluorescence emission intensity of DCFH at around 525 nm with increasing irradiation time. In contrast, TTMIndo derivatives with electron-withdrawing substituents, TTMIndoBr, TTMIndoN, and TTMIndoNO_2_, exhibited only modest ROS generation capabilities (Fig. S6 and S9a†). Surprisingly, TTMIndoMe exhibited no detectable ROS generation, potentially attributable to its low water solubility causing the aggregation-caused quenching (ACQ) effect (Fig. S5†). Among them, TTMIndoOMe demonstrated an approximately 2.08-fold higher ROS generation efficiency than the commercial PSs Chlorin e6 (Ce6) and 1.26-fold higher than Rose Bengal (RB) (Fig. 2d and S10a–c†). To further elucidate the nature of the ROS generated by TTMIndo derivatives, the probes 9,10-anthracenediyl-bis(methylene)dimalonic acid (ABDA) and dihydroethidium (DHE) were utilized to detect ^1^O_2_ and O_2_˙^−^, respectively. Under normoxic conditions, we hypothesized that radicals would not trigger ^1^O_2_ generation upon irradiation because energy could not transfer to ambient triplet oxygen molecules. To confirm this and investigate ^1^O_2_ generation by TTMIndo derivatives, we measured the absorption decrease at 380 nm in the presence of ^1^O_2_ using the ABDA probe and found a negligible generation of ^1^O_2_ by the TTMIndo derivatives under irradiation (Fig. 2c, S7 and S9c†). In contrast, we observed significant generation of O_2_˙^−^ by the TTMIndo π-radicals with electron-donating substituents, as indicated by the increased fluorescence emission intensity of DHE at around 625 nm (Fig. 2b, S8 and S9b†). The TTMIndoOMe compound showed approximately 2.00-fold higher O_2_˙^−^ generation efficiency than the commercial PSs Ce6 and 3.29-fold higher than RB, indicating its potential as an effective PS for type I PDT (Fig. 2e and S10d–f†). In addition, we compared the ROS and O_2_˙^−^ generation efficiency of TTMIndoOMe with that of Methylene Blue (MB), a well-known type I and II photosensitizer. The comparisons of ROS and O_2_˙^−^ generation abilities of TTMIndoOMe and MB were conducted under both hypoxic (O_2_% = 2%) and ambient conditions. The results depicted in Fig. 2f–i demonstrate that TTMIndoOMe's ROS and O_2_˙^−^ generation were 1.39-fold and 2.47-fold higher than that of MB under hypoxic (O_2_% = 2%) conditions and 1.20-fold and 1.56-fold higher than that of MB under ambient conditions (Fig. S11 and S12†) due to the ^1^O_2_ generation for MB as a type II photosensitizer under ambient conditions. These results indicate that TTMIndoOMe exhibits superior type I photosensitizing capabilities for PDT against hypoxia. The proposed mechanism of TTMIndo π-radicals as a PS involves the excitation of the doublet ground state (D_0_) to its doublet excited state (D_1_) under irradiation, followed by electron exchange with triplet oxygen or biological substrates to produce O_2_˙^−^*via* the type I PDT mechanism (Fig. S13†). Our results demonstrated that the introduction of substituents with push–pull electron effects in the radical compounds of TTMIndo derivatives allowed for precise modulation of the equilibrium between stability and the ability to generate ROS.

### *In vitro* evaluation of ROS generation capability and photocytotoxicity of TTM π-radical PSs

**Fig. 3:**
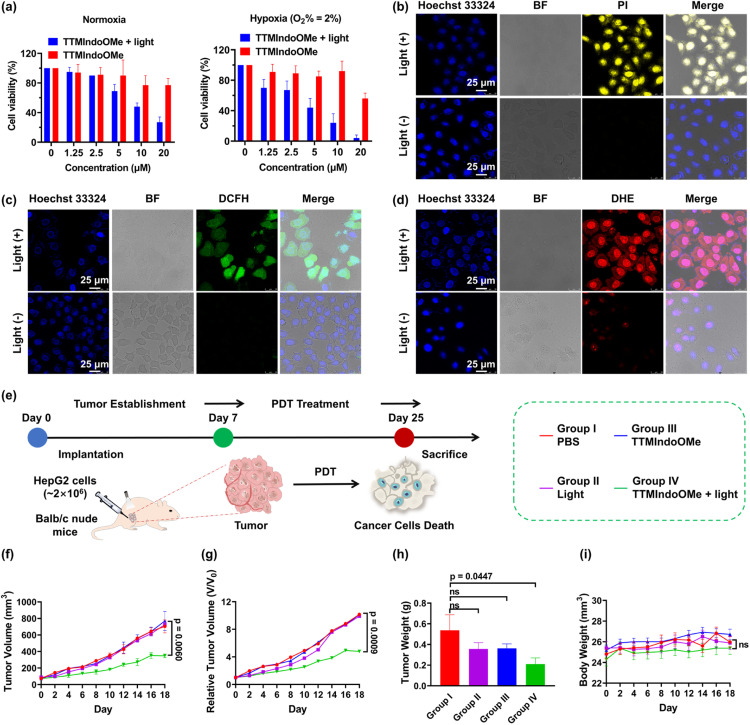
(a) HepG2 cell cytotoxicity of TTMIndoOMe in the dark (red) and upon white light irradiation (20 mW cm^−2^, 10 minutes) (blue) under normoxic (left) and hypoxic (O_2_% = 2%) (right) conditions; data are given as the mean ± SD (*n* = 3). (b) Confocal fluorescence images of dead HepG2 cells stained under normoxic conditions (PI, dead cell marker, *λ*_ex_ = 488 nm, and *λ*_em_ = 600–650 nm). Scale bar: 25 μm. Confocal fluorescence images of intracellular ROS (c) and O_2_˙^–^ (d) in HepG2 cells under normoxic conditions (DCFH, ROS generation marker, green, *λ*_ex_ = 488 nm, and *λ*_em_ = 495–550 nm) and (DHE, O_2_˙^–^ generation marker, red, *λ*_ex_ = 535 nm, and *λ*_em_ = 600–650 nm). Scale bar: 25 μm. (e) Schematic illustration of the *in vivo* experimental procedures. (f) The tumor growth curves in all groups. (g) The relative tumor growth curves in all groups. (h) The tumor weight from all groups on the 18^th^ day post treatment. (i) The mice body weight curves in all groups. For the TTMIndoOMe plus light treatment group in the *in vivo* experiment, the dose of TTMIndoOMe was 100 μL PBS, 20 mg kg^−1^ by subcutaneous injections, and irradiated with white LED light (20 mW cm^−2^, 15 minutes). Data are given as the mean ± SEM (*n* = 6) *in vivo*.

Given TTMIndoOMe's superior ROS generation capability, we investigated its photostability and stability in cell lysates. The absorption bands at ∼610 nm of TTMIndoOMe remained unchanged, indicating its good photostability (Fig. S10†). For stability evaluation, HPLC analysis was conducted on the lysates of different cells after incubation with TTMIndoOMe for various durations (Fig. S14–S17†). Notably, TTMIndoOMe exhibited excellent stability in B16, A549, and HepG2 tumor cells. Therefore, we selected TTMIndoOMe as a potential photosensitizer and investigated its anticancer effect on HepG2, A549, and B16 cells using an MTS assay. As depicted in Fig. 3 and S18,†TTMIndoOMe exhibited a dose-dependent inhibition of cell proliferation under light irradiation, while showing negligible cytotoxicity to HepG2, A549, and B16 cells in the dark. To explore the phototherapeutic potential of TTMIndoOMe in treating hypoxic tumors, we examined its PDT effect on HepG2 cells in a hypoxic environment (O_2_% = 2%). Encouragingly, upon light irradiation, TTMIndoOMe demonstrated a dose-dependent inhibition of cell proliferation, indicating its potential as an effective phototherapeutic agent (Fig. 3a).

Inspired by the promising photocytotoxicity of TTMIndoOMe against cancer cells, even under hypoxic conditions, we further investigated its effectiveness *via* confocal fluorescence imaging under normoxia and hypoxia (O_2_% = 2%), using an all-cell/dead-cell co-staining viability assay with Hoechst 33324/propidium iodide (PI), as illustrated in Fig. 3b and S15.† Intracellular ROS production was also confirmed using DCFH-DA/DHE, with green/bright red fluorescence assigned to the generation of total ROS/O_2_˙^−^ (Fig. 3c, d and S19–S21†). These observations were consistent with the results discussed above.

### PDT efficacy of TTMIndoOMe in immunocompetent nude mice models with HepG2 tumors

After demonstrating the PDT efficacy of TTMIndoOMe*in vitro*, we proceeded to investigate its efficacy *in vivo* using a HepG2 tumor-bearing immunocompetent nude mice model. The mice were randomly divided into four groups (six mice per group): PBS only (group I), light only (group II), TTMIndoOMe only (group III), and TTMIndoOMe with light irradiation (group IV). Tumor volumes were recorded every 2 days for each group (Fig. 3e and S22†). The results indicated that TTMIndoOMe with light irradiation partially suppressed tumor growth, whereas no tumor suppression was observed in the groups treated with groups I–III (Fig. 3f–h and j). Moreover, the body weight of the mice in each group showed no differences during treatment (Fig. 3i), indicating no side effects of TTMIndoOMe on the mice. These results demonstrate the *in vivo* PDT efficacy of TTMIndoOMe in the tumor microenvironment and its good biocompatibility, suggesting the potential for future PDT radical PS design.

### Induction of ICD by TTMIndoOMe on B16, A549 and HepG2 cells

**Fig. 4:**
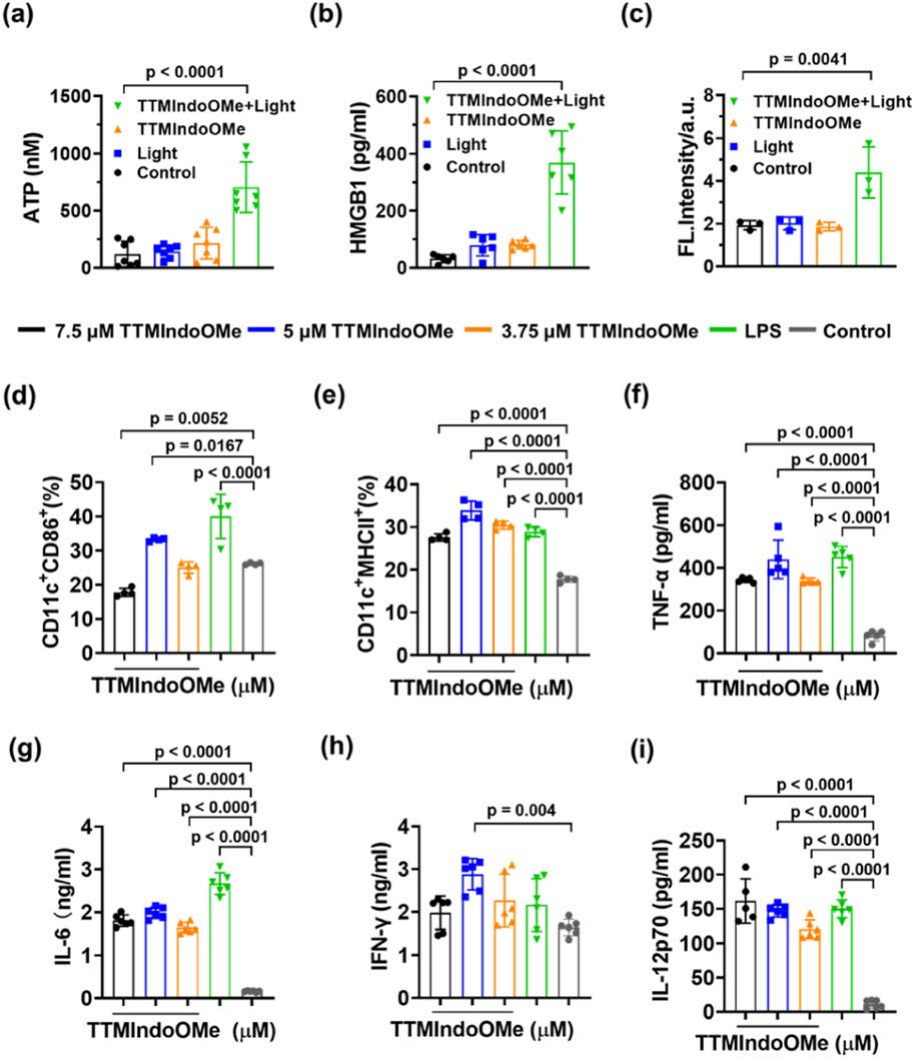
Characterization of TTMIndoOMe plus light-treated B16 tumor cells *in vitro*. ATP secretion (nM) (a) and HMGB1 release (pg mL^−1^) (b) from B16 cells upon indicated treatments (*n* = 6). (c) FL intensity of CRT exposure on the membrane of B16 cells upon indicated treatment capture using a confocal fluorescence microscope (*n* = 3). (d) Flow cytometric examination of CD11c^+^CD86^+^ DC population (%) and (e) CD11c^+^MHCII^+^ DC population (%) stimulated by B16 cells after indicated treatments. Secretion of TNF-α (f), IL-6 (g), IFN-γ (h), and IL-12p70 (i) by BMDCs measured using ELISA after indicated treatments. Data are given as the mean ± SD (*n* = 6).

The above study demonstrated that TTMIndoOMe can cause cancer cell death by necrosis and/or apoptosis, and we further investigated the PDI effect of TTMIndoOMe on B16, A549 and HepG2 cells. The tumor cells treated with TTMIndoOMe plus light irradiation exhibited significantly elevated levels of ATP (Fig. 4a, S23a and c†) and extracellular HMGB1 compared to those treated with TTMIndoOMe or light irradiation alone (Fig. 4b, S23b and d†). Moreover, immunofluorescence staining revealed CRT expression on the membrane of B16, A549 and HepG2 cells treated with TTMIndoOMe plus light irradiation (Fig. 4c and S24–S26†). These findings suggest that the combined treatment of TTMIndoOMe and light irradiation induces a more profound ICD effect on tumor cells compared to individual treatments.

Subsequently, we assessed the ICD on the maturation of DCs *in vitro*. Flow cytometric analysis revealed that B16 cells treated with TTMIndoOMe (5 μM) plus light irradiation exhibited enhanced DC maturation, as indicated by increased expression of CD11c^+^CD86^+^ (30.48 ± 3.50%) and CD11c^+^MHCII^+^, (33.03 ± 2.15%) compared to the control group (CD11c^+^CD86^+^, 26.18 ± 0.32%; CD11c^+^MHCII^+^, 22.00 ± 3.36%) (Fig. 4d and e). Consistent with these findings, the combined treatment of TTMIndoOMe and light irradiation induced the highest secretion of immune-related cytokines (TNF-α, IL-6, IFN-γ, and IL-12p70) compared to individual treatments (Fig. 4f–i). These results demonstrate the ability of TTMIndoOMe, in combination with light irradiation, to effectively penetrate B16 cells and enhance intracellular ROS generation. This ultimately resulted in direct cytotoxicity towards B16 cells and ICD responses. Consequently, TTMIndoOMe holds promising potential as a photodynamic immunotherapeutic agent against B16 tumor cells.

### Evaluation of the immunotherapy efficacy of TTMIndoOMe against B16 tumors *in vivo*

**Fig. 5:**
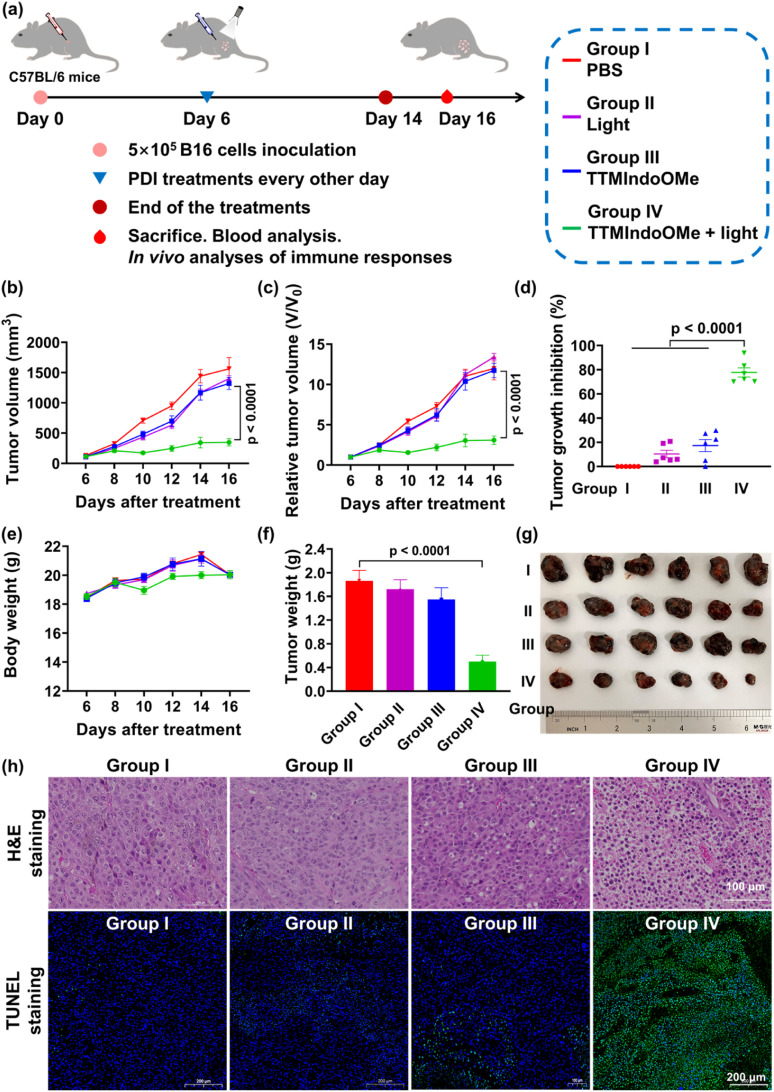
Evaluation of TTMIndoOMe plus light for photodynamic immunotherapy of B16 tumor in mice. (a) Schematic illustration of the experimental design. (b) Tumor growth curves, (c) relative tumor volume, (d) tumor growth inhibition and (e) body weight of B16 tumor-bearing mice within 10 days after different treatments as indicated. (f) B16 tumor weight on day 16 after different treatments as indicated. (g) Representative photograph of B16 tumors collected from all groups at the end of treatments. (h) H&E and TUNEL staining of tumor tissue slices resected from mice after indicated treatments. Scale bar: 100 μm for H&E and 200 μm for TUNEL. Group I: PBS; group II: model + light; group III: model + TTMIndoOMe; group IV: model + TTMIndoOMe + light. For the TTMIndoOMe plus light treatment group in the *in vivo* experiment, the dose of TTMIndoOMe was 100 μL PBS, 15 mg kg^−1^ by subcutaneous injections, and irradiated with white LED light (20 mW cm^−2^, 15 minutes). Data are given as the mean ± SEM (*n* = 6) *in vivo*.

Having demonstrated the ICD effect of TTMIndoOMe plus light-treated B16 tumor cells *in vitro*, we next evaluated the therapeutic efficacy of TTMIndoOMe against B16 tumors in C57BL/6 mice. Once the tumor size reached ∼100 mm^3^, the mice were randomly divided into different treatment groups: PBS only (group I), light alone (group II), TTMIndoOMe alone (group III), TTMIndoOMe plus light (group IV), and blank control (group V) (Fig. 5a). 30 min after subcutaneous injection of PBS or TTMIndoOMe (15 mg kg^−1^ in PBS, 100 μL), the tumors were either subjected to irradiation (groups II and IV) or left non-irradiated (groups I, III and V). The body weight and tumor volume of each mouse were monitored every 2 days.

Notably, compared to the PBS-treated group (group I), significant retardation in tumor growth was observed in mice receiving TTMIndoOMe plus light treatment (group IV) (Fig. 5b and c). After PDI treatment, the tumor growth inhibition of group IV was nearly 80%, exhibiting its efficient PDI therapeutic efficiency (Fig. 5d). Importantly, no significant changes in the body weight were observed following different treatments (Fig. 5e), and routine blood tests revealed no apparent alterations caused by TTMIndoOMe administration (Fig. S27†). Consistently, the tumor weight in the TTMIndoOMe plus light treatment group (group IV) was significantly decreased on day 14 compared to the PBS-treated group (group I) (Fig. 5f). Histological examination of tumor tissue slices using hematoxylin and eosin (H&E) staining and terminal deoxynucleotidyl transferase dUTP nick end labelling (TUNEL) staining confirmed that TTMIndoOMe plus light treatment induced greater tumor cell death (Fig. 5g and h). These findings affirm TTMIndoOMe's favourable safety profile in mice, highlighting its potential as a low-toxicity therapeutic agent.

**Fig. 6:**
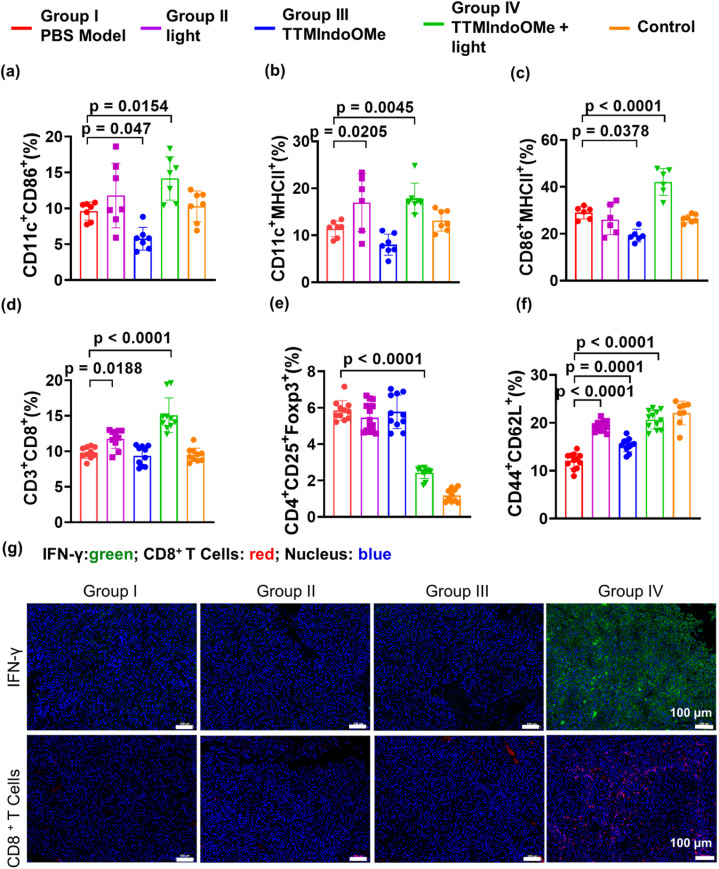
TTMIndoOMe plus light treatment elicits strong anti-tumor immunity. Flow cytometric analysis of DC maturation of CD11c^+^CD86^+^ (a), CD11c^+^MHCII^+^ (b) and CD86^+^MHCII^+^ (c) in draining lymph nodes (dLNs). (d) Flow cytometric analysis of CD3^+^CD8^+^ T cells in tumor bearing mice after indicated treatments. (e) Flow cytometric analysis of the Tregs (CD4^+^CD25^+^Foxp3^+^ T cells) in tumor bearing mice. (f) Flow cytometric analysis of CD44^+^CD62L^+^ T cells in the spleen of tumor bearing mice after indicated treatments. Blue: model + PBS; red: model + light; green: model + TTMIndoOMe; purple: model + TTMIndoOMe + light; orange: control. (g) Immunofluorescence staining of IFN-γ and CD8^+^ T cells in the tumor tissues after indicated treatments. Scale bars: 100 μm. Data are given as the mean ± SEM (*n* = 6) *in vivo*.

We further investigated the *in vivo* ICD effect and immune response induced by TTMIndoOMe. The CRT exposure in B16 tumors treated with TTMIndoOMe + light (group IV) was significantly higher compared to tumors treated with light (group II) or TTMIndoOMe alone (group III). Flow cytometric analysis of DC maturation in the draining lymph nodes (LNs) at 15 days post-treatment showed a higher proportion of mature DCs in group IV (CD11c^+^CD86^+^, 14.17 ± 1.15%; CD11c^+^MHCII^+^, 17.84 ± 1.24%; CD86^+^MHCII^+^, 42.13 ± 2.33%) compared to PBS-treated mice (group I, CD11c^+^CD86^+^, 9.62 ± 0.47%; CD11c^+^MHCII^+^, 11.39 ± 0.64%; CD86^+^MHCII^+^, 29.00 ± 1.08%) (Fig. 6a–c and S28a–c†). This indicates that the induction of ICD by TTMIndoOMe plus light promotes DC maturation in the LNs (Fig. 6a–c). Immunofluorescence staining of IFN-γ and CD8^+^ T cells in the tumor tissues confirmed increased secretion of IFN-γ and CD8^+^ T cells in group IV compared to the other groups (Fig. 6g and S29†), indicating a prominent proinflammatory response elicited by TTMIndoOMe plus light. The measurement of T lymphocytes in the spleen and tumors revealed a significantly higher percentage of CD3^+^CD8^+^ T cells in group IV (15.08 ± 0.77%) compared to group I, group II, and group III (Fig. 6d and S28d†). In the B16 tumor mouse model, TTMIndoOMe plus light treatment markedly suppressed the percentage of Tregs (CD4^+^CD25^+^Foxp3^+^ T cells) in group IV (2.42 ± 0.11%) compared to the PBS-treated group (group I, 5.85 ± 0.19%) (Fig. 6e and S28e†). Consequently, the CD8^+^ T cells to Tregs ratio (CD8^+^ T cells/Tregs) in group IV (6.30 ± 1.00%) was the highest among all the groups (group I: 1.69 ± 0.24%; group II: 2.23 ± 0.38%; group III: 1.69 ± 0.36%; group V: 8.86 ± 3.18%) and corresponded to the strongest inhibition of tumor growth observed in group IV (Fig. 6d, e and S28d, e†). Furthermore, the evaluation of immune memory effects in B16 tumor-bearing C57BL/6 mice demonstrated a significant increase in the population of memory T lymphocytes (Tem: CD44^+^CD62L^+^) from 12.43 ± 0.45% in PBS-treated mice to 20.56 ± 0.82% in mice treated with TTMIndoOMe plus light (group IV) on day 10 post-treatment (Fig. 6f and S28f†). Immunofluorescence staining of CD8^+^ T cells and IFN-γ further supported that TTMIndoOMe plus light treatment effectively promoted the recruitment of effector T cells within the tumor tissues (Fig. 6g). This suggests a more profound systemic immune response induced by TTMIndoOMe plus light, contributing to tumor growth prevention. Our experiments collectively demonstrated that TTMIndoOMe, upon with light irradiation, elicited potent ICD responses, enhanced DC maturation, recruited effector T cells while suppressing Tregs, and modulated memory T cells to activate a robust systemic immune response. These effects significantly inhibited the growth of B16 tumors, highlighting the potential of TTMIndoOMe and light as an effective strategy for cancer treatment.

## Conclusions

In summary, we proposed a novel and effective strategy for designing PS based on organic free radicals to enhance PDI under hypoxic conditions. By incorporating aromatic indole structures into the TTM core, we obtained a series of stable π-radicals. Through modulation of the push–pull electron effects of the substituents, we discovered that incorporating electron-donating groups into TTM radicals enhanced the ROS generation upon irradiation. By investigating the type of ROS generated, we revealed that the organic π-radical TTMIndoOMe generated O_2_˙^−^ through electron transfer. Notably, TTMIndoOMe demonstrated remarkable ICD induction and facilitated the maturation of DCs, enabling efficient presentation of tumor-specific antigens to naïve T cells and triggering a robust anti-tumor immune response. Our strategy effectively enhanced ROS generation and induced ICD in cancer cells by utilizing doublet π-radicals. These findings will open new avenues for the development of next-generation PSs and the design of innovative PDT/PDI strategies targeting hypoxic tumors. Moreover, these discoveries also offer new possibilities for the design of controlled-release PS complexes, further enhancing the efficacy and precision of cancer treatments.

## Ethical statement

All animal experiments were approved by the Animal Care and Use Committee of the Institute of Materia Medica, Chinese Academy of Medical College (Approval No. 00003996 and 00004181).

## Data availability

ESI† is available and includes the synthesis and characterizations of compounds, the detections of cellular ROS levels, cell cytotoxicity, immunogenic cell death upon white LED light irradiation, the *in vivo* experiments of photodynamic immunotherapy efficacy and antitumor immune response.

## Author contributions

H.-Y. H., C.-F. C. and T. Z. conceived the research, designed the research approach, and supervised the study; X. W. and G. S. designed and performed the experiments and conducted the data analysis with the help of R. W., L. M. and Q. Z.; X. W., G. S., T. Z., C.-F. C. and H.-Y. H. wrote the manuscript with input from all the authors. All authors have given approval to the final version of the manuscript.

## Conflicts of interest

There are no conflicts to declare.

## Supplementary Material

SC-015-D3SC06826A-s001
