# Supplementary material for: Fine-tuning of stable organic free-radical photosensitizers for photodynamic immunotherapy
Source: Chem Sci. 2024 Apr 3;15(17):6421–31. doi: 10.1039/d3sc06826a (PMC11062115; doi:10.1039/d3sc06826a)
Supplement: SC-015-D3SC06826A-s001 [file SC-015-D3SC06826A-s001.pdf]

Electronic Supplementary Material (ESI) for Chemical Science.
This journal is © The Royal Society of Chemistry 2024

Fine-Tuning of Stable Organic Free-Radical Photosensitizers for Photodynamic Immunotherapy

Xiang Wang[‡,1], Gaona Shi[‡,1], Rao Wei[1], Meng Li[2], Qingyang Zhang[1], Tiantai Zhang[1,*], Chuan-Feng Chen[2,*] and Hai-Yu Hu[1,*]

[1] State Key Laboratory of Bioactive Substances and Function of Natural Medicine, Beijing Key Laboratory of Active Substances Discovery and Draggability Evaluation, Institute of Materia Medica, Chinese Academy of Medical Sciences & Peking Union Medical College, Beijing, 100050, China.

[2] Beijing National Laboratory for Molecular Sciences, CAS Key Laboratory of Molecular Recognition and Function, Institute of Chemistry, Chinese Academy of Sciences, Beijing 100190, China

Keywords: photodynamic immunotherapy • organic neutral π-radical • photosensitizer • hypoxic environment.

[*]Corresponding Author(s): Tiantai Zhang: ttzhang@imm.ac.cn; Chuan-Feng Chen: cchen@iccas.ac.cn; Hai-Yu Hu: haiyu.hu@imm.ac.cn.

[‡]X. Wang and G. Shi contributed equally to this work.

Abstract**:** Photodynamic immunotherapy (PDI) is an innovative approach to cancer treatment that utilizes photodynamic therapy (PDT) and photosensitizers (PSs) to induce immunogenic cell death (ICD). However, currently most used PSs have restricted capabilities to generate reactive oxygen species (ROS) via a Type-II mechanism under hypoxic environments, which limits their effectiveness in PDI. To overcome this, we propose a novel approach for constructing oxygen independent PSs based on stable organic free-radical molecules. By fine-tuning the characteristics of TTM radical through the incorporation of electron-donating moieties, we successfully found that TTMIndoOMe could produce substantial amounts of ROS even in hypoxic environments. In vitro experiments showed TTMIndoOMe could effectively produce $O_2^{\bullet-}$, kill tumor cells and trigger ICD. Moreover, in vivo experiments also demonstrated that TTMIndoOMe could further trigger anti-tumor immune response and exhibit a superior therapeutic effect compared with PDT alone. Our study offers a promising approach towards the development of next-generation PSs functioning efficiently even in hypoxic conditions, and also paves the way for the creation of more effective PSs for PDI.

Contents

1. Abbreviations and General Methods -- S3

2. Synthetic Procedures and Characterized Data -- S4

3. UV-vis Absorption and Fluorescence Spectra -- S9

4. EPR Spectra and Double Integrals and Spins of EPR Signals ---------------------------------- S9

5. DFT Calculations: Molecular Orbital Energy Levels -- S10

6. DFT Calculations: Mülliken Spin Densities Values -- S11

7. ROS, $^1O_2$ and $O_2^{\cdot-}$ Generation Capabilities of PSs -- S12

8. Cell Culture --- S15

9. The Photostability Evaluation of **TTMIndoOMe** -- S15

10. The Stability Evaluation of **TTMIndoOMe** in Different Cell Lysates ------------------------- S15

11. Dark and Light Cell Cytotoxicity of the **TTMIndoOMe** under Normoxic and Hypoxic (O_2% = 2%) Conditions --- S17

12. Confocal Fluorescence Images of HepG2 Cells Incubated with **TTMIndoOMe** under Normoxic and Hypoxic (O_2% = 2%) Conditions --- S17

13. Animals and Tumor Models-- S19

14. Measurement of TNF-α, IFN-γ, IL-12p70 and IL-6 -- S20

15. DCs Maturation *in vitro* -- S20

16. Immunogenic Cell Death Induction of **TTMIndoOMe** *in vitro* --------------------------------- S20

17. Anti-Tumor Effect of **TTMIndoOMe** *in vivo* -- S23

18. Evaluation of the Safety of **TTMIndoOMe** *in vivo* --- S23

19. Antitumor Immune Response *in vivo* -- S24

20. Statistical Analysis --- S26

21. NMR Spectra --- S27

22. References --- S35

Experimental Procedures

Abbreviations

ABDA = 9,10-Anthracenediyl-bis(methylene)dimalonic Acid

DCFH = 2′,7′-Dichlorofluorescin

DCFH-DA = 2′,7′-Dichlorofluorescin Diacetate

DCM = Dichloromethane

DFT = Density Functional Theory

DHE = Dihydroethidium

DMEM = Dulbecco's Modified Eagle Medium

DMF = *N,N*-Dimethylformamide

DMSO = Dimethyl Sulfoxide

ELISA = Enzyme Linked Immunosorbent Assay

EPR = Electron Paramagnetic Resonance

ESI = Electrospray Ionisation

FBS = Fetal Bovine Serum

HRMS = High Resolution Mass Spectrometry

MIN = Min

NMR = Nuclear Magnetic Resonance

PE = Petrol Ether

rpm = Revolutions per minute

r.t. = Room Temperature

ROS = Reactive Oxygen Species

TFA = Trifluoroacetic Acid

THF = Tetrahydrofuran

TTM = Tris-(2,4,6-Trichlorophenyl)-Methane

Results and Discussion

General Methods

All the chemicals were purchased from Innochem. Commercially available reagents were used without further purification. TTM was purchased from Pharmaron Beijing Co., Ltd. (China). Fluorescence emission spectra and full wavelength absorption spectra were performed on a 2300 EnSpire multimode plate reader. EPR spectra were performed on a Bruker ElexSys E580 spectrometer. The confocal laser scanning microscopic imaging studies were conducted with Leica TCS SP2 Confocal Microscope and Zeiss LSM880 Confocal Microscope. All ^{1}H NMR spectra were recorded at 400 MHz. ^{13}C NMR spectra were recorded at 100 MHz. HRMS were measured with a Thermo LCQ Deca XP Max mass spectrometer for ESI. MALDI-TOF mass spectra were recorded on a Bruker Autoflex III-MALDI-TOF-MS with DCTB as a matrix. Light sources (white light, 20 mW/cm^{2}) for chemical reactions and bioassays were from LED Light provided by PURI Materials, Shenzhen. The hypoxic environments *in vitro* experiments were constructed by SANYO O_2/CO_2 incubator MCO-5M, SANYO Electric Co., Ltd.. The HPLC analysis was performed on the Thermo U3000, with chromatographic separation achieved on an InfinityLab Poroshell 120 EC-C18 column (150 mm × 4.6 mm, 4 μm particle size). A linear gradient from 50% B (0.1% formic acid in methanol) to 100% B (0.1% formic acid in methanol) in 5 min at a flow rate of 0.6 mL/min, and 100% B (0.1% formic acid in methanol) in 18 min at a flow rate of 0.6 mL/min with a detection wavelength of 600 nm was applied. Flow cytometric analysis of cells was conducted with BD FACSVERSE (BD Inc., USA). The human cancer cell lines HepG2 and A549 and mouse cancer cell line B16 in this study were from the Cell Resource Centre, Institute of Basic Medical Sciences, Chinese Academy of Medical Sciences & Peking Union Medical College. Dulbecco's modified eagle medium (DMEM), Roswell Park Memorial Institute (RPMI) 1640, and fetal bovine serum (FBS) were purchased from Gibco Company (USA). Penicillin and streptomycin were purchased from HyClone Company (USA). Anti-CD11c-APC, anti-CD86-PE, anti-CD4-FITC, anti-CD8a-PE, anti-CD25-APC, anti-FoxP3-PE, anti-MHC II-Percpcy5.5, anti-CD44-PE, and anti-CD62L- APC antibodies were all purchased from BioLegend, Inc. (San Diego, USA). Antibody for Calreticulin (CRT) was obtained from Abcam (Cambridge, UK). The ELISA kit for HMGB1 was purchased from Solarbio Science & Technology Co. Ltd (Beijing, China). The ELISA kit for ATP was obtained from Beyotime Biotech, Inc (Beijing, China). The antibody IFN-γ was purchased from Bioss Antibodies (Beijing, China). The ELISA Kits for IL-12p70, IL6, IFN-γ, and TNF-α detecting were purchased from Thermo Fisher Scientific (Waltham, MA, USA). The evaluation of biosafety was carried out by Mindray BC-5150.

Synthetic Procedures and Characterized Data

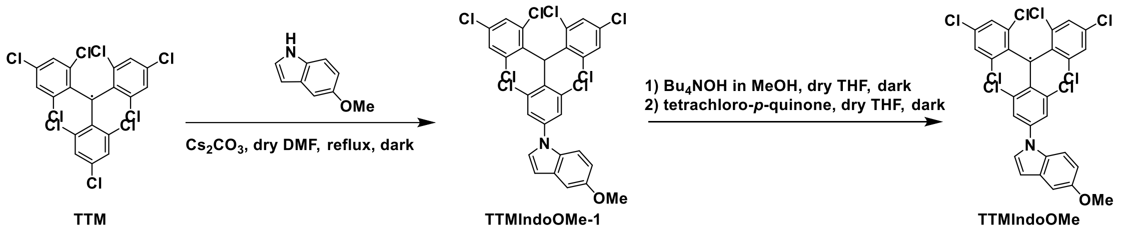

Scheme S1. Synthesis of **TTMIndoOMe**.

TTMIndoOMe-1

In a 50 mL round bottom flask, TTM (553.3 mg, 1 mmol) was dissolved in 10 mL dry DMF. Then 5-Methoxyindole (441.5 mg, 3 mmol) and anhydrous Cs_2CO_3 (977.5 mg, 3 mmol) were added and the solution was stirred degassed (Ar) at 160 °C for 4 h in the dark. After cooling to r.t., the resulting mixture was poured into (1 M) hydrochloric acid solution, and the precipitate was filtered. The residue was purified by silica gel column chromatography (PE : DCM = 2 : 1) to give green solid 232.75 mg, yielding 35%. ^{1}H NMR (400 MHz, Chloroform-*d*): *δ* 7.52-7.49 (m, 2H, -Ar), 7.39-7.37 (m, 3H, -Ar), 7.28-7.24 (m, 3H, -Ar), 7.11 (s, 1H, -Ar), 6.91 (d, *J* = 6.8 Hz, 1H, -Ar), 6.91 (s, 1H, -CH), 6.61 (s, 1H, -Ar), 3.87 (s, 3H, -CH_3). ^{13}C NMR (100 MHz, Chloroform-*d*): *δ* 155.1, 140.0, 138.3, 138.1, 137.2, 134.1, 133.8, 132.3, 130.5, 130.1, 130.1, 128.5, 127.5, 124.5, 122.9, 113.0, 111.3, 105.0, 103.2, 55.8, 49.9. HRMS (ESI): Calcd for $C_{28}H_{15}NOCl_8^+$ ([M]$^+$), 660.8662, found, 660.8663.

TTMIndoOMe

In a 25 mL round bottom flask, **TTMIndoOMe**-1 (66.5 mg, 0.1 mmol) was dissolved in 6 mL of dry THF, tetrabutylammonium hydroxide solution in methanol (1.5 M) (135 μL, 0.2 mmol) was added and the solution was stirred degassed (Ar) for 1 h in the dark. Then, tetrachloro-pbenzoquinone (49.2 mg, 0.2 mmol) was added to the mixture. The mixture was stirred for another 1 h in the dark. After the reaction was completed, solvent was concentrated under vacuum, and the residue was purified by silica gel column chromatography (PE : DCM = 2 : 1) to give green solid 47 mg, yielding 71%. HRMS (ESI): Calcd for $C_{28}H_{14}NOCl_8^+$ ($[M]^+$), 659.8578, found, 659.8584.

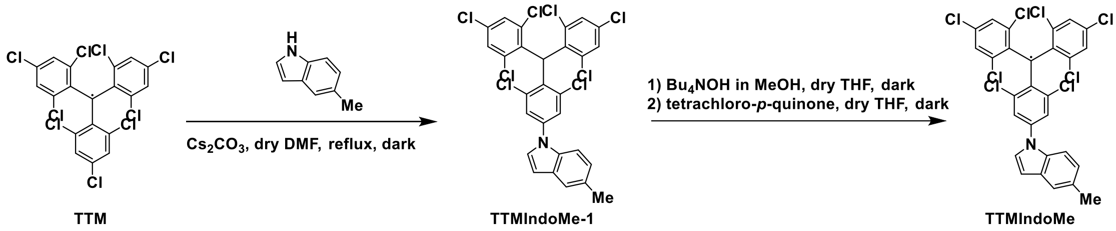

Scheme S2. Synthesis of **TTMIndoMe**.

TTMIndoMe-1

In a 50 mL round bottom flask, TTM (553.3 mg, 1 mmol) was dissolved in 10 mL dry DMF. Then 5-methylindole (393.5 mg, 3 mmol) and anhydrous Cs_2CO_3 (977.5 mg, 3 mmol) were added and the solution was stirred degassed (Ar) at 160 °C for 4 h in the dark. After cooling to r.t., the resulting mixture was poured into (1 M) hydrochloric acid solution, and the precipitate was filtered. The residue was purified by silica gel column chromatography (PE : DCM = 2 : 1) to give green solid 181.7 mg, yield 28%. ^1H NMR (400 MHz, Chloroform-*d*): δ 7.53 (d, J = 2.0 Hz, 1H, -Ar), 7.50 (d, J = 8.4 Hz, 1H, -Ar), 7.46 (s, 1H, -Ar), 7.40-7.39 (m, 3H, -Ar), 7.28-7.27 (m, 1H, -Ar), 7.27-7.25 (m, 2H, -Ar), 7.10 (d, J = 8.8 Hz, 1H, -Ar), 6.79 (s, 1H, -CH), 6.62 (d, J = 3.2 Hz, 1H, -Ar), 2.47 (s, 3H, $-CH_3$). ^{13}C NMR (100 MHz, Chloroform-*d*): δ 138.3, 137.6, 134.3, 133.9, 132.4, 130.7, 130.23, 130.17, 128.6, 127.2, 124.8, 123.4, 123.2, 121.3, 110.2, 105.0, 50.1, 21.5. HRMS (ESI): Calcd for $C_{28}H_{14}NCl_8^-$ ($[M-1]^-$), 643.8629, found, 643.8633.

TTMIndoMe

In a 25 mL round bottom flask, TTMIndoMe-1 (64.9 mg, 0.1 mmol) was dissolved in 6 mL of dry THF, tetrabutylammonium hydroxide solution in methanol (1.5 M) (135 μL, 0.2 mmol) were added and the solution was stirred degassed (Ar) for 1 h in the dark. Then, tetrachloro-pbenzoquinone (49.2 mg, 0.2 mmol) was added to the mixture. The mixture was stirred for another 1 h in the dark. After the reaction was completed, solvent was concentrated under vacuum, and the residue was purified by silica gel column chromatography (PE : DCM = 2 : 1) to give green solid 47.3 mg, yield 73%. HRMS (ESI): Calcd for $C_{28}H_{14}NCl_8^+$ ($[M]^+$), 643.8629, found, 643.8638.

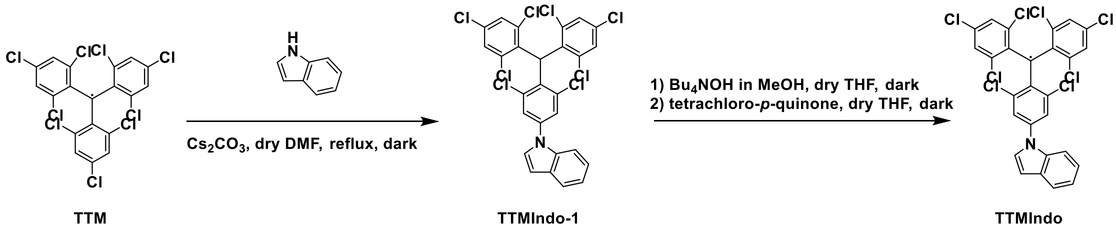

Scheme S3. Synthesis of **TTMIndo.**

TTMIndo-1

In a 50 mL round bottom flask, TTM (553.3 mg, 1 mmol) was dissolved in 10 mL dry DMF. Then indole (351.5 mg, 3 mmol) and anhydrous Cs_2CO_3 (977.5 mg, 3 mmol) were added and the solution was stirred degassed (Ar) at 160 °C for 4 h in the dark. After cooling to r.t., the resulting mixture was poured into (1 M) hydrochloric acid solution, and the precipitate was filtered. The residue was purified by silica gel column chromatography (PE : DCM = 4 : 1) to give green solid 292.1 mg, yield 46%. ^1H NMR (400 MHz, Chloroform-*d*): δ 7.69 (d, J = 7.6 Hz, 1H, -Ar), 7.62 (d, J = 8.4 Hz, 1H, -Ar), 7.56 (d, J = 2.4 Hz, 1H, -Ar), 7.42-7.40 (m, 3H, -Ar), 7.32 (d, J = 3.2 Hz, 1H, -Ar), 7.29-7.26 (m, 3H, -Ar), 7.21 (t, J = 7.2 Hz, 1H, -Ar), 6.81 (s, 1H, -CH), 6.72 (d, J = 3.2 Hz, 1H, -Ar). ^{13}C NMR (100 MHz, Chloroform-*d*): δ 139.9, 138.3, 138.2, 138.1, 137.6, 137.3, 137.3, 135.4, 134.2, 133.9, 132.6, 130.2, 130.2,129.8, 128.6, 127.2, 125.0, 123.4, 123.2, 121.6, 121.3, 110.5, 105.4, 50.0. HRMS (MALDI): Calcd for $C_{27}H_{12}NCl_8^-$ ($[M-1]^+$), 629.8468, found, 629.8472.

TTMIndo

In a 25 mL round bottom flask, TTMIndo-1 (63.5 mg, 0.1 mmol) was dissolved in 6 mL of dry THF, tetrabutylammonium hydroxide solution in methanol (1.5 M) (135 μL, 0.2 mmol) were added and the solution was stirred degassed (Ar) for 1 h in the dark. Then, tetrachloro-pbenzoquinone (49.2 mg, 0.2 mmol) was added to the mixture. The mixture was stirred for another 1 h in the dark. After the reaction was completed, solvent was concentrated under vacum, and the residue was purified by silica gel column chromatography (PE : DCM = 2 : 1, v/v) to give green solid 47.6 mg, yield 75%. HRMS (ESI): Calcd for $C_{27}H_{12}NCl_8^+$ ($[M]^+$), 629.8469, found, 629.8472.

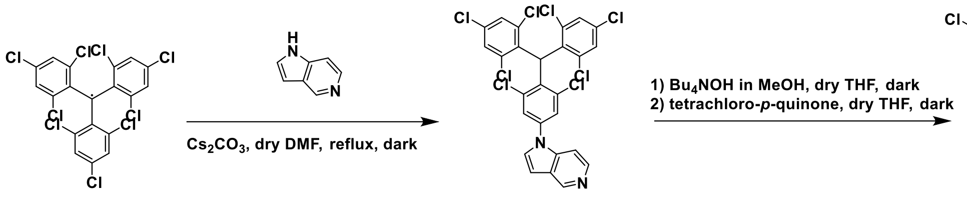

TTM TTMIndoN-1 TTMIndoN

Scheme S4. Synthesis of **TTMIndoN.**

TTMIndoN-1

In a 50 mL round bottom flask, TTM (553.3 mg, 1 mmol) was dissolved in 10 mL dry DMF. Then 5-azaindole (354.4 mg, 3 mmol) and anhydrous Cs_2CO_3 (977.5 mg, 3 mmol) were added and the solution was stirred degassed (Ar) at 160 °C for 4 h in the dark. After cooling to r.t., the resulting mixture was poured into (1 M) hydrochloric acid solution, and the precipitate was filtered. The residue was purified by silica gel column chromatography (PE : EA = 2 : 1, v/v) to give red solid 286.2 mg, yielding 45%. ^{1}H NMR (400 MHz, $CDCl_3$): *δ* 8.99 (s, 1H, -Ar), 8.41 (s, 1H, -Ar), 7.52 (s, 1H, -Ar), 7.48 (d, *J* = 4.8 Hz, 1H, -Ar), 7.39 (s, 3H, -Ar), 7.36 (d, *J* = 2.8 Hz, 1H, -Ar), 7.26 (s, 2H, -Ar), 6.81 (s, 1H, -CH), 6.80 (s, 1H, -Ar). ^{13}C NMR (100 MHz, $CDCl_3$): *δ* 144.3, 142.2, 138.9, 138.4, 137.8, 137.7, 137.6, 137.00, 136.97, 133.72, 133.67, 133.63, 133.57, 129.94, 129.91, 128.4, 128.3, 128.0, 126.0, 124.8, 123.2, 105.4, 104.2, 49.8. HRMS (ESI): $C_{26}H_{13}N_2Cl_8^+$ ($[M+1]^+$), 632.8582, found, 632.8530.

TTMIndoN

In a 25 mL round bottom flask, TTMIndoN-1 (63.6 mg, 0.1 mmol) was dissolved in 6 mL of dry THF, tetrabutylammonium hydroxide solution in methanol (1.5 M) (135 μL, 0.2 mmol) was added and the solution was stirred degassed (Ar) for 1 h in the dark. Then, tetrachloro-pbenzoquinone (49.2 mg, 0.2 mmol) was added to the mixture. The mixture was stirred for another 1 h in the dark. After the reaction was completed, solvent was concentrated under vacuum, and the residue was purified by silica gel column chromatography (PE : EA = 2 : 1, v/v) to give red solid 53.2 mg, yield 84%. HRMS (ESI): Calcd for $C_{26}H_{12}N_2Cl_8^+$ ($[M+1]^+$), 631.8503, found, 631.8464.

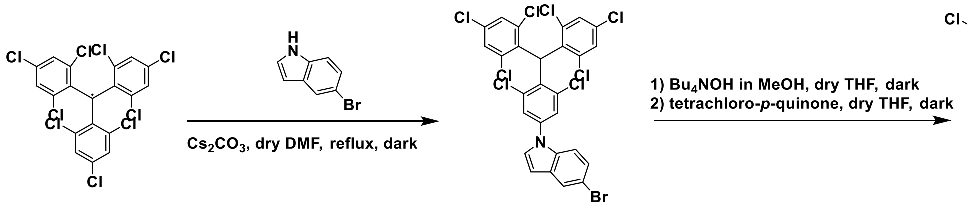

TTM TTMIndoBr-1 TTMIndoBr

Scheme S5. Synthesis of **TTMIndoBr.**

TTMIndoBr-1

In a 50 mL round bottom flask, TTM (553.3 mg, 1 mmol) was dissolved in 10 mL dry DMF. Then 5-bromoindole (588.12 mg, 3 mmol) and anhydrous Cs_2CO_3 (977.5 mg, 3 mmol) were added and the solution was stirred degassed (Ar) at 160 °C for 4 h in the dark. After cooling to r.t., the resulting mixture was poured into (1 M) hydrochloric acid solution, and the precipitate was filtered. The residue was purified by silica gel column chromatography (PE : DCM = 4 : 1) to give red solid 286.2 mg, yield 45%. ^{1}H NMR (400 MHz, $CDCl_3$): *δ* 7.80 (s, 1H, -Ar), 7.50 (d, *J* = 2.0 Hz, 1H, -Ar), 7.56 (d, *J* = 2.4 Hz, 1H, -Ar), 7.45 (d, *J* = 8.8 Hz, 1H, -Ar), 7.40 (t, *J* = 2.4 Hz, 2H, -Ar), 7.37 (d, *J* = 2.0 Hz, 1H, -Ar), 7.35-7.33 (m, 1H, -Ar), 7.31 (d, *J* = 3.2 Hz, 1H, -Ar), 7.27 (s, 2H, -Ar), 6.81 (s, 1H, -CH), 6.64 (d, *J* = 3.2 Hz, 1H, -Ar). ^{13}C NMR (100 MHz, $CDCl_3$): *δ* 139.4, 138.5, 138.1, 137.7, 137.3, 134.12, 134.05, 133.9, 133.2, 131.4, 130.2, 128.6, 128.4, 126.0, 125.0, 124.1, 123.5, 114.4, 111.9, 104.7, 50.0. HRMS (MALDI): Calcd for $C_{27}H_{11}BrNCl_8^-$ ($[M-1]^-$), 707.7574, found, 707.7578.

TTMIndoBr

In a 25 mL round bottom flask, TTMIndoBr-1 (71.4 mg, 0.1 mmol) was dissolved in 6 mL of dry THF, tetrabutylammonium hydroxide solution in methanol (1.5 M) (135 μL, 0.2 mmol) were added and the solution was stirred degassed (Ar) for 1 h in the dark. Then, tetrachloro-pbenzoquinone (49.2 mg, 0.2 mmol) was added to the mixture. The mixture was stirred for another 1 h in the dark. After the reaction was completed, solvent was concentrated under vacum, and the residue was purified by silica gel column chromatography (PE : DCM = 4 : 1) to give red solid 52.0 mg, yield 73%. HRMS (MADLI): Calcd for $C_{27}H_{11}BrNCl_8^+$ ([M]$^+$), 707.7571, found, 707.7578.

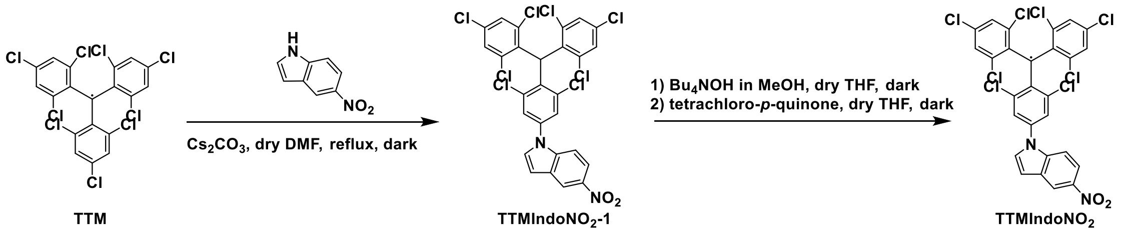

Scheme S6. Synthesis of **TTMIndoNO$_2$**.

TTMIndoNO$_2$-1

In a 50 mL round bottom flask, TTM (553.3 mg, 1 mmol) was dissolved in 10 mL dry DMF. Then 5-nitroindole (483.45 mg, 3 mmol) and anhydrous Cs_2CO_3 (977.5 mg, 3 mmol) were added and the solution was stirred degassed (Ar) at 160 °C for 4 h in the dark. After cooling to r.t., the resulting mixture was poured into (1 M) hydrochloric acid solution, and the precipitate was filtered. The residue was purified by silica gel column chromatography (PE : DCM = 2 : 1) to give red solid 286.2 mg, yield 45%. ^{1}H NMR (400 MHz, Chloroform-*d*): δ 8.63 (d, J = 2.4 Hz, 1H, -Ar), 8.17 (dd, J_1 = 9.2 Hz, J_2 = 2.4 Hz, 1H, -Ar), 7.58 (d, J = 9.2 Hz, 1H, -Ar), 7.53 (d, J = 2.0 Hz, 1H, -Ar), 7.47 (t, J = 3.6 Hz, 1H, -Ar), 7.40 (t, J = 2.0 Hz, 3H, -Ar), 7.27 (d, J = 2.0 Hz , 2H, -Ar), 6.98 (d, J = 3.6 Hz, 1H, -Ar), 6.80 (s, 1H, -CH). ^{13}C NMR (100 MHz, Chloroform-*d*): δ 142.8, 138.8, 138.6, 138.3, 138.1, 138.0, 137.3, 134.5, 134.1, 133.9, 133.9, 130.6, 130.3, 130.2, 129.1, 128.7, 128.7, 125.6, 124.0, 118.7, 118.6, 110.6, 107.0, 50.1. HRMS (MALDI): Calcd for $C_{27}H_{12}N_2O_2Cl_8^+$ ([M]$^+$), 675.8407, found, 675.8407.

TTMIndoNO$_2$

In a 25 mL round bottom flask, TTMIndoNO$_2$-1 (68.0 mg, 0.1 mmol) was dissolved in 6 mL of dry THF, tetrabutylammonium hydroxide solution in methanol (1.5 M) (135 μL, 0.2 mmol) was added and the solution was stirred degassed (Ar) for 1 h in the dark. Then, tetrachloro-pbenzoquinone (49.2 mg, 0.2 mmol) was added to the mixture. The mixture was stirred for another 1 h in the dark. After the reaction was completed, the solvent was concentrated under vacuum, and the residue was purified by silica gel column chromatography (PE : DCM = 2 : 1) to give red solid 55.0 mg, yielding 81%. HRMS (MADLI): Calcd for $C_{27}H_{11}N_2O_2Cl_8^+$ ([M]$^+$), 674.8321, found, 674.8323.

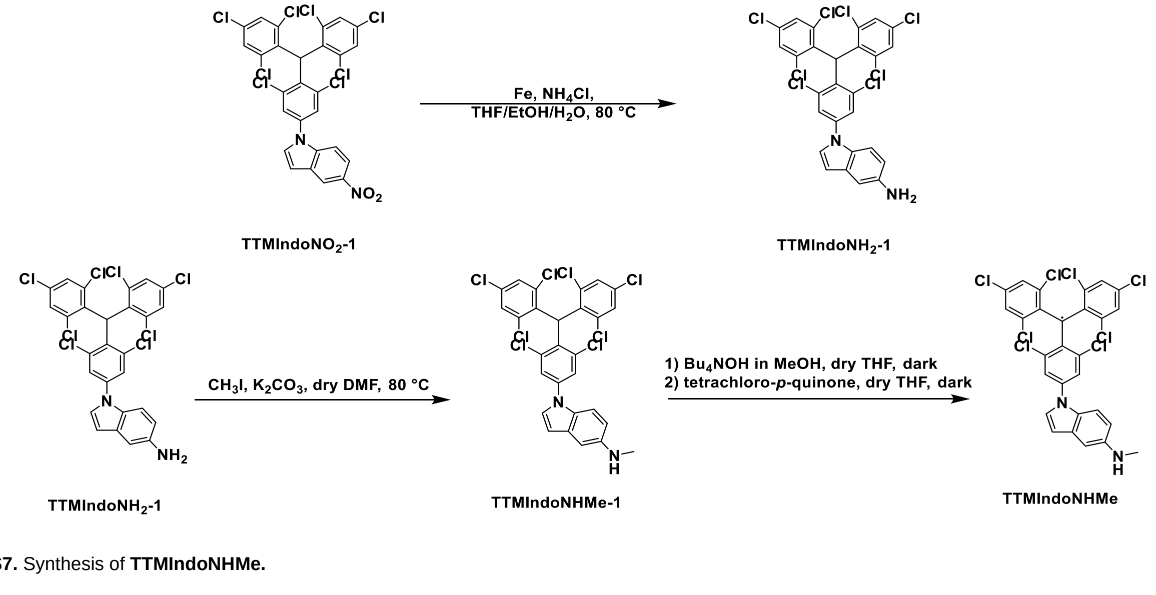

Scheme S7. Synthesis of **TTMIndoNHMe.**

TTMIndoNH$_2$-1

In a 50 mL round bottom flask, TTMIndoNO_2-1 (204.0 mg, 0.3 mmol) was dissolved in 5 mL THF, 5 mL EtOH and 2.5 mL H_2O. Then NH_4Cl (32.1 mg, 0.6 mmol) and Fe (33.6 mg, 0.6 mmol) were added and the solution was stirred at 80 ^oC overnight. After cooling to r.t., the resulting mixture was filtered with celite and filtrate was evaporated under vacuum. The residue was purified by silica gel column chromatography (PE : DCM = 1 : 4) to give green solid 171.6 mg, yielding 86%. ^1H NMR (400 MHz, Chloroform-*d*): δ 7.50 (t, J = 2.0 Hz 2H, -Ar), 7.42 (d, J = 8.6 Hz, 1H, -Ar), 7.38 (dq, J_1 = 6.5 Hz, J_2 = 2.1 Hz, 3H, -Ar), 7.26 (d, J = 3.2 Hz, 2H, -Ar), 7.23 (t, J = 2.4 Hz, 1H, -Ar), 6.94 (d, J = 2.3 Hz, 1H, -Ar), 6.77 (s, 1H, -CH), 6.71 (dd, J_1 = 8.6 Hz, J_2 = 2.3 Hz, 1H, -Ar), 6.52 (d, J = 3.3 Hz, 1H, -Ar), 3.59 (s, 2H, $-NH_2$). ^{13}C NMR (100 MHz, Chloroform-*d*): δ 142.9, 141.0, 140.2, 138.3, 137.5, 134.3, 133.9, 132.0, 131.1, 130.2, 128.6, 127.4, 124.3, 122.8, 113.5, 111.2, 106.2, 104.6, 50.0. HRMS (ESI): Calcd for $C_{27}H_{15}N_2Cl_8^+$ ($[M+1]^+$), 646.8739, found, 646.8743.

TTMIndoNHMe-1

In a 38 mL seal tube, TTMIndoNH_2-1 (195.0 mg, 0.3 mmol) was dissolved in 5 mL dry DMF. Then K_2CO_3 (124.4 mg, 0.9 mmol) and CH_3I (213.0 mg, 1.5 mmol) were added and the solution was stirred at 80 ^oC for overnight in the dark. After cooling to r.t., the resulting mixture was extracted by EA three times. Then, the organic phase was combined and dried by Na_2SO_4. The solvent was concentrated under vacuum and then residue was purified by silica gel column chromatography (PE : DCM = 2 : 1) to give green solid 155.4 mg, yield 78%. ^1H NMR (400 MHz, Chloroform-*d*): δ 7.51 (s, 1H, -Ar), 7.44 (d, J = 6.8 Hz, 1H, -Ar), 7.40-7.36 (m, 3H, -Ar), 7.22 (s, 1H, -Ar), 6.85 (s, 1H, -Ar), 6.72 (s, 1H, -Ar), 6.67 (d, J = 6.8 Hz, 1H, -Ar), 6.55 (s, 1H, -CH), 2.90 (s, 3H, $-CH_3$). ^{13}C NMR (100 MHz, Chloroform-*d*): δ 145.6, 141.3, 139.2, 138.3, 135.3, 134.8, 132.1, 131.1, 130.4, 129.6, 128.1, 126.6, 125.2, 123.6, 113.5, 112.2, 105.9, 103.4, 51.0, 32.9. HRMS (ESI): Calcd for $C_{28}H_{18}N_2Cl_8^+$ ($[M+2]^+$), 6612.8973, found, 661.8928.

TTMIndoNHMe

In a 25 mL round bottom flask, TTMIndoNHMe-1 (66.4 mg, 0.1 mmol) was dissolved in 6 mL of dry THF, tetrabutylammonium hydroxide solution in methanol (1.5 M) (135 μL, 0.2 mmol) were added and the solution was stirred degassed (Ar) for 1 h in the dark. Then, tetrachloro-pbenzoquinone (49.2 mg, 0.2 mmol) was added to the mixture. The mixture was stirred for another 1 h in the dark. After the reaction was completed, the solvent was concentrated under vacuum, and the residue was purified by silica gel column chromatography (PE : DCM = 2 : 1) to give green solid 41.1 mg, yield 62%. HRMS (ESI): Calcd for $C_{28}H_{17}N_2Cl_8^+$ ($[M+2]^+$), 660.8895, found, 660.8899.

UV-vis Absorption Spectra

The TTMIndo derivatives compounds were each dissolved in chloroform to generate stock solutions of 10 mM, respectively. Immediately before UV-Vis measurement, aliquots of fluorogen stocks were diluted in chloroform, to obtain a final concentration of 10 μM. The **TTMIndoOMe** was each dissolved in DMSO to generate stock solutions of 10 mM, respectively. Immediately before UV-Vis measurement, aliquots of fluorogen stocks were diluted in PBS and water respectively, to obtain a final concentration of 20 μM. Wavelength interval: 2.0 nm.

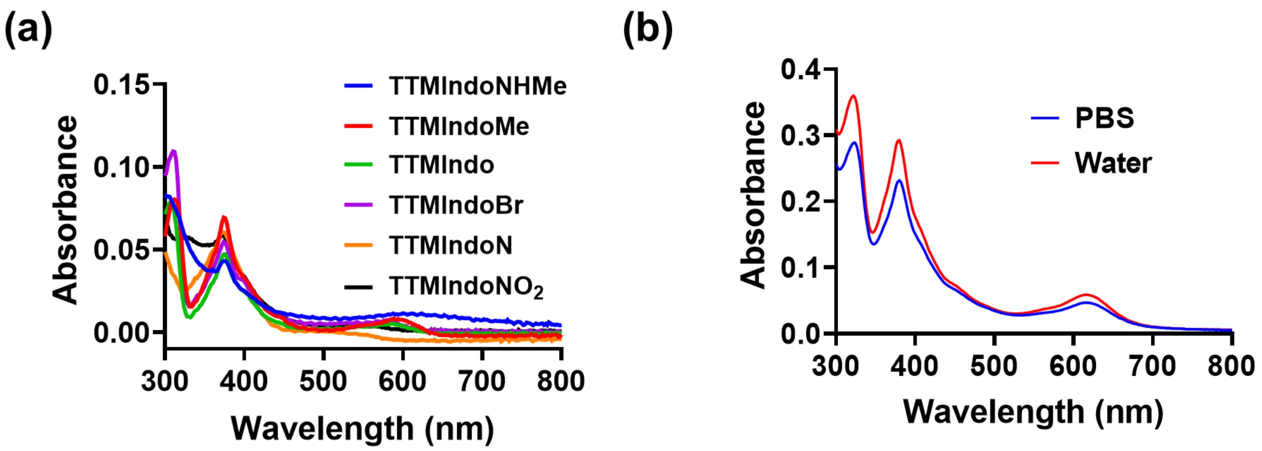

Figure S1. (a) Absorption spectra of 10 μM **TTMIndo** derivative compounds in chloroform. (b) Absorption spectra of 20 μM **TTMIndoOMe** compounds in PBS and water.

EPR Spectra and Double Integrals and Spins of EPR Signals

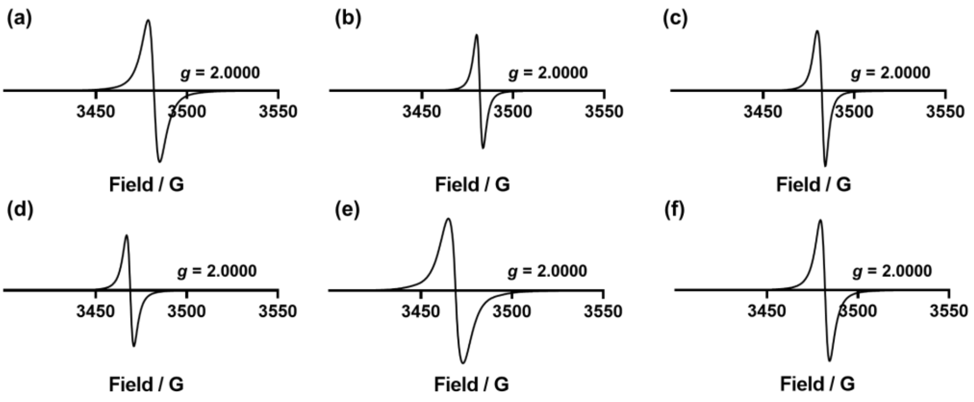

Figure S2. The EPR spectra of (a) **TTMIndoNHMe**, (b) **TTMIndoMe**, (c) **TTMIndo**, (d) **TTMIndoN**, (e) **TTMIndoNO₂**, (f) **TTMIndoBr** in toluene (4 mM) measured at room temperature.

Table S1. Double integrals and spins of EPR signals

Compound	Area / a.u.	Spins / mm^3
**TTMIndoOMe**	1733	5.649×10^{15}
**TTMIndoNHMe**	1093	3.563×10^{15}
**TTMIndoMe**	2342	7.638×10^{15}
**TTMIndo**	1778	5.727×10^{15}
**TTMIndoN**	1389	4.474×10^{15}
**TTMIndoNO$_2$**	527	1.721×10^{15}

**TTMIndoBr**	1258	4.202×10^{15}

DFT Calculations: Molecular Orbital Energy Levels

The DFT calculations were performed with the Gaussian16 series of programs[1] using the B3LYP hybrid functional and 6-31G(d) basis set. The spin densities and Mülliken spin densities were calculated by Multiwfn[2]. Molecular orbital energy levels were made with Multiwfn and VMD 1.9.3 software support. The isovalue of the figures are 0.03 au.

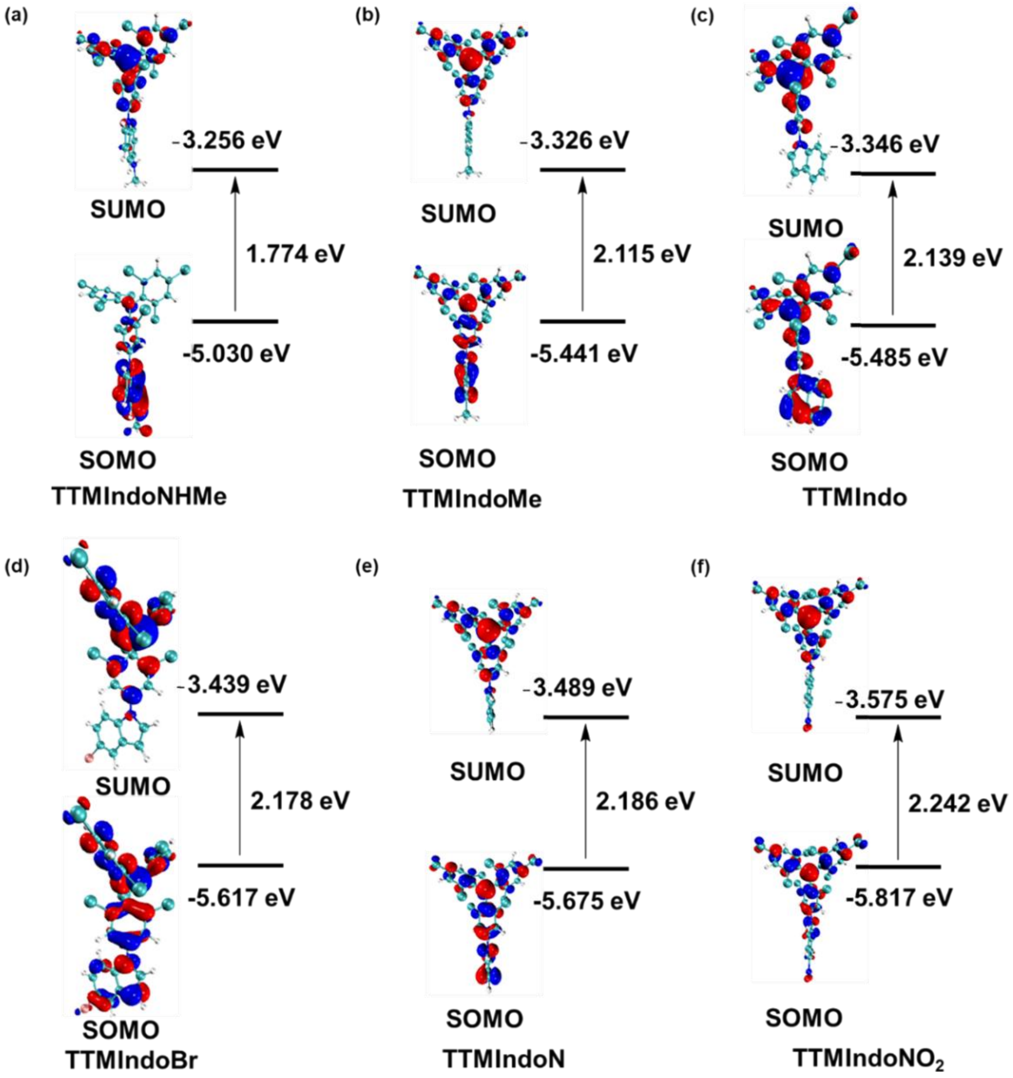

Figure S3. Molecular orbital energy levels of the **TTMIndo** derivative compounds. The energy levels (left panels) and contour plots (right panels) of the molecular orbitals (SUMO and SOMO) of (a) **TTMIndoNHMe**, (b) **TTMIndoMe**, (c) **TTMIndo**, (d) **TTMIndoBr**, (e) **TTMIndoN**, (f) **TTMIndoNO$_2$.**

DFT Calculations: Mülliken Spin Densities Values

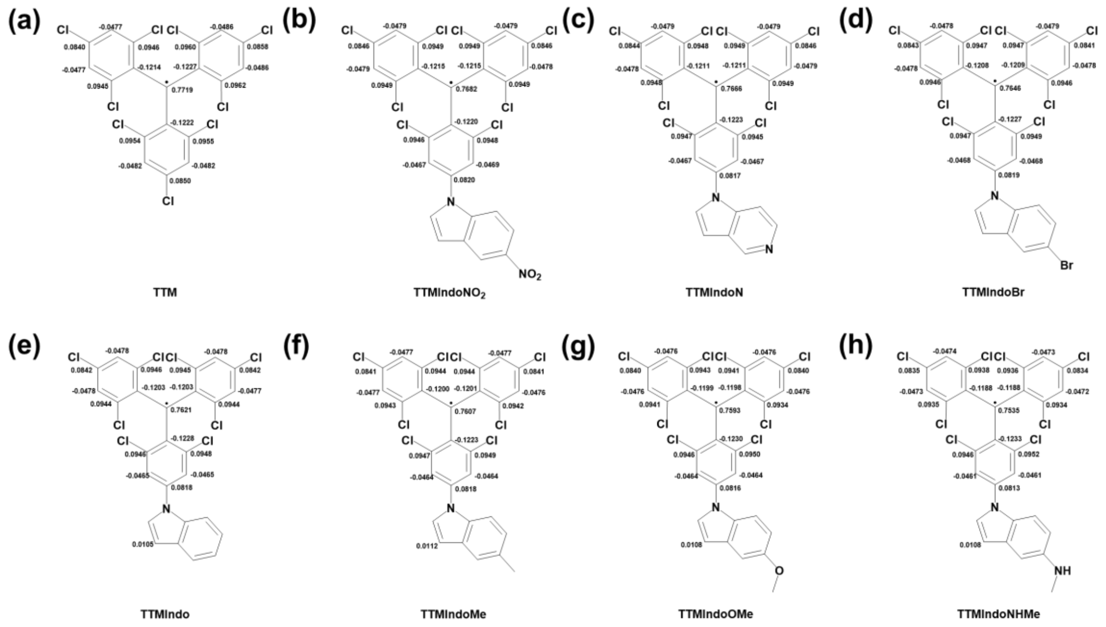

Figure S4. Mülliken spin densities values at the B3LYP/6-31G* level of the **TTMIndo** derivative compounds and **TTM**. (a)**TTM**, (b)**TTMIndoNO₂**, (c)**TTMIndoN**, (d)**TTMIndoBr**, (e)**TTMIndo**, (f)**TTMIndoMe**, (g)**TTMIndoOMe**, (h)**TTMIndoNHMe**. Only the sites with values higher than 0.01 are shown.

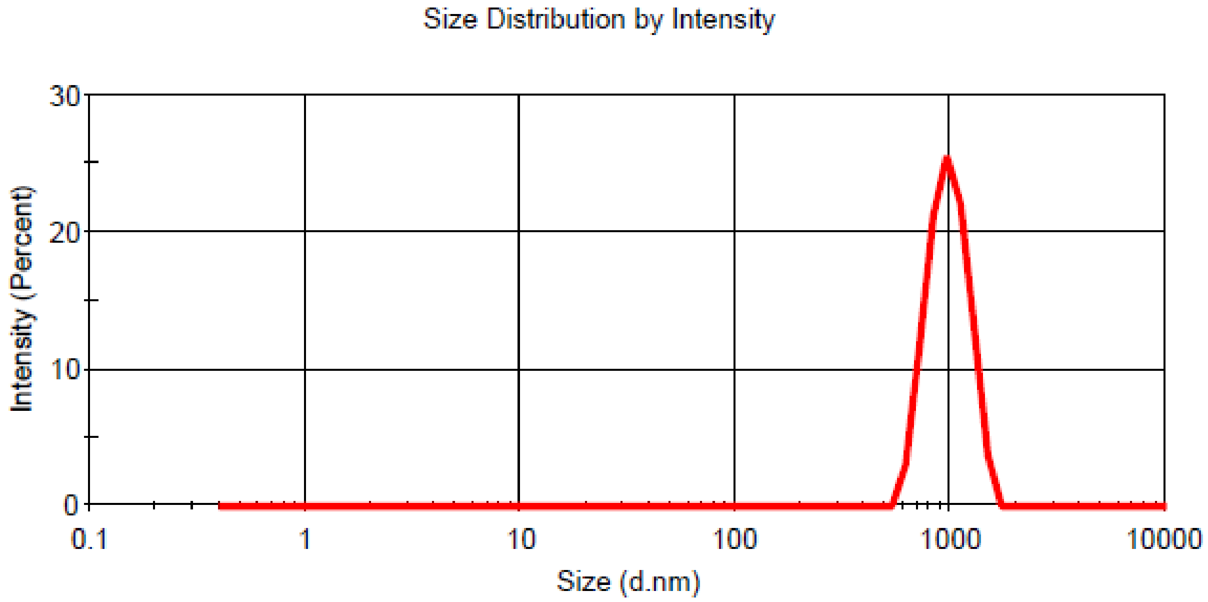

Figure S5. Representative size distributions of **TTMIndoMe** in water with 1% DMSO, measured by DLS (n = 3).

ROS, $^1O_2$ and $O_2^{\cdot-}$ Generation Capabilities of PSs

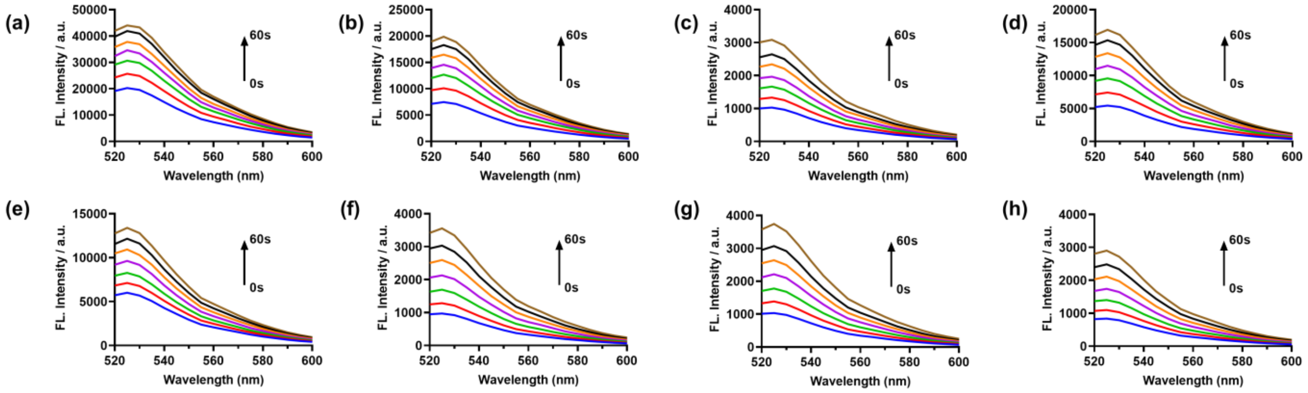

Figure S6. ROS generation capability of PSs indicated by DCFH. (a) **TTMIndoOMe**, (b) **TTMIndoNHMe**, (c) **TTMIndoMe**, (d) **TTMIndo**, (e) **TTMIndoNO₂**, (f) **TTMIndoBr**, (g) **TTMIndoN**, (h) DCFH, DCFH (40 μM) fluorescent spectra incubated with **TTMIndo** derivative compounds (10 μM) in PBS (λ_{ex} = 480 nm) upon 20 mW/cm^2 white light LED irradiation.

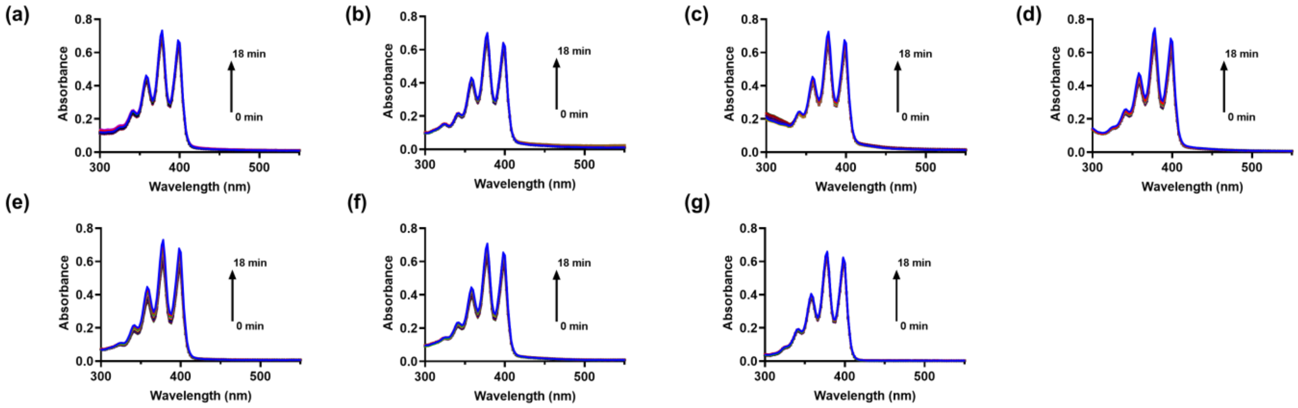

Figure S7. $^1O_2$ Generation Capabilities of PSs Indicated by ABDA. (a) **TTMIndoNHMe**, (b) **TTMIndoMe**, (c) **TTMIndo**, (d) **TTMIndoNO₂**, (e) **TTMIndoBr**, (f) **TTMIndoN**, (g) ABDA, ABDA (50 μM) absorbance spectra incubated with **TTMIndo** derivative compounds (10 μM) in water upon 20 mW/cm^2 white light LED irradiation.

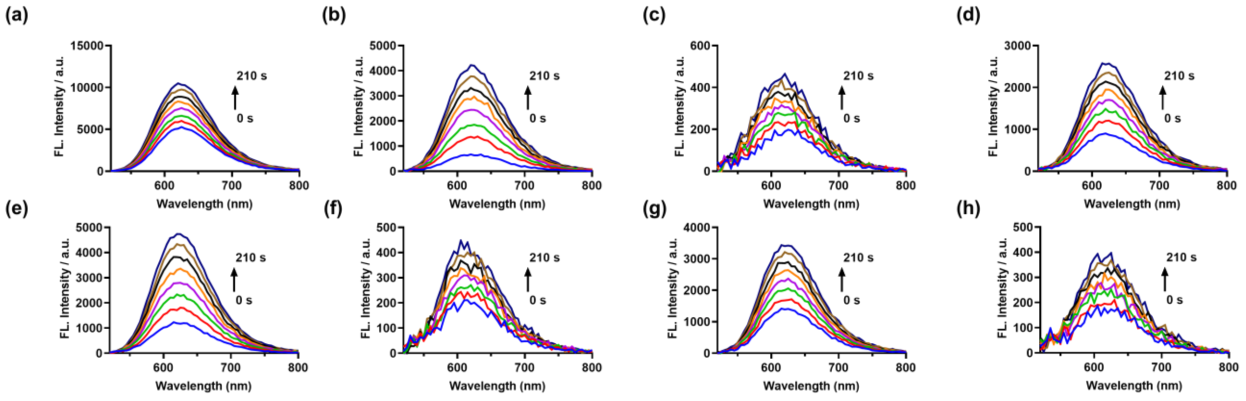

Figure S8. $O_2^{\cdot-}$ generation capabilities of PSs indicated by DHE. (a) **TTMIndoOMe**, (b) **TTMIndoNHMe**, (c) **TTMIndoMe**, (d) **TTMIndo**, (e) **TTMIndoNO₂**, (f) **TTMIndoBr**, (g) **TTMIndoN**, (h) DHE, DHE (20 μM) fluorescent spectra incubated with **TTMIndo** derivative compounds (10 μM) in PBS upon 20 mW/cm^2 white light LED irradiation (λ_{ex} = 535 nm).

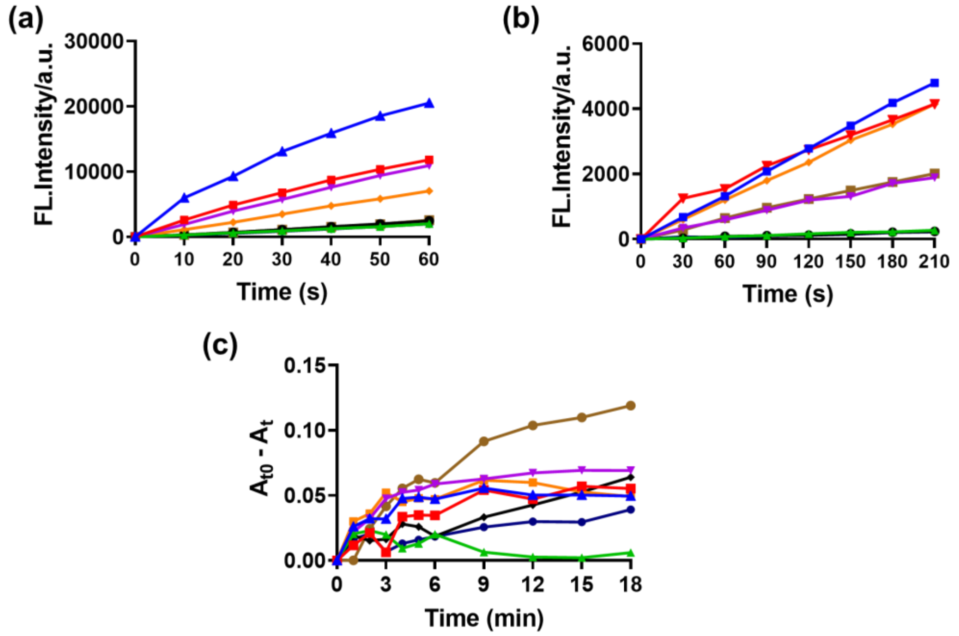

Figure S9. (a) ROS generation evaluated by DCFH (40 μM) in PBS with or without the TTMIndo derivative compounds (10 μM) fluorescent emission at 520 nm as a function of the 20 mW/cm^2 white light LED irradiation duration (λ_{ex} = 480 nm). (b) $O_2^{\cdot-}$ generation evaluated by DHE (20 μM) in PBS with or without the TTMIndo derivative compounds (10 μM) fluorescent emission at 625 nm as a function of the 20 mW/cm^2 white light LED irradiation duration (λ_{ex} = 535 nm). (c) $^1O_2^{\cdot-}$ generation evaluated by ABDA (50 μM) in water with or without the TTM-Indo derivative compounds (10 μM) absorption at 380 nm as a function of the 20 mW/cm^2 white light LED irradiation duration. Blue: **TTMIndoOMe,** Red: **TTMIndoNHMe**, Green: **TTMIndoMe**, Purple: **TTMIndo**, Orange: **TTMIndoNO$_2$**, Black: **TTMIndoBr**, Brown: **TTMIndoN**, Dark Blue: ROS Indicator.

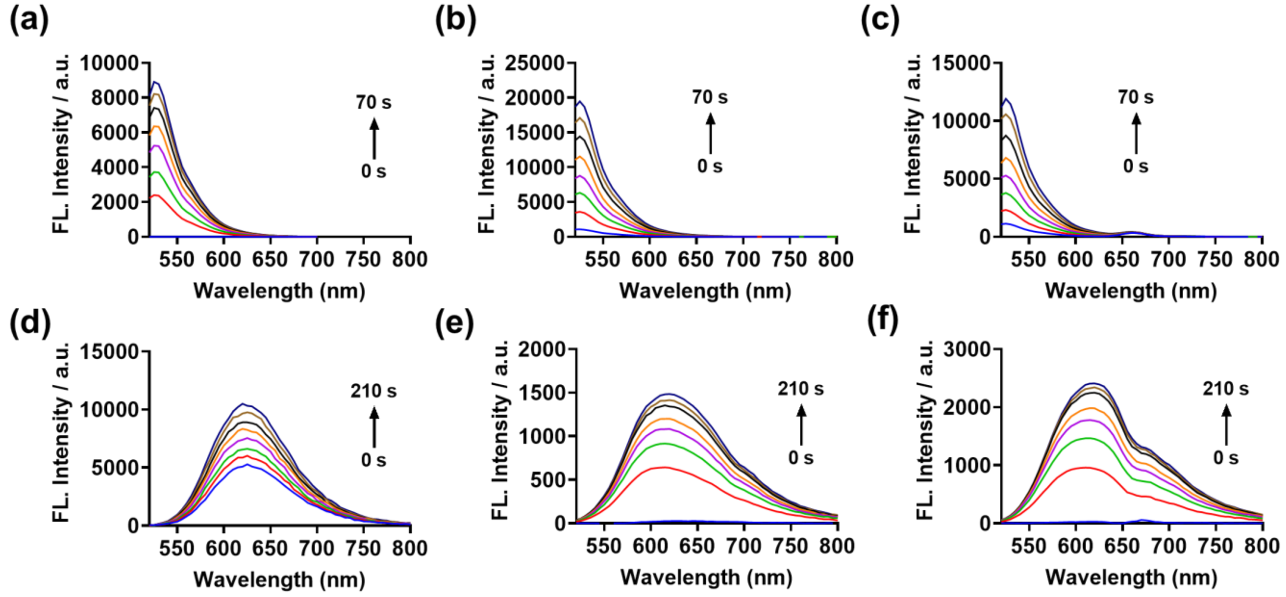

Figure S10. ROS generation of **TTMIndoOMe** (a), and commercial PSs RB (b) and Ce6 (c) in PBS (λ_{ex} = 480 nm) upon irradiation by the 20 mW/cm^2 white light LED irradiation. $O_2^{\cdot-}$ generation of **TTMIndoOMe** (d), and commercial PSs RB (e) and Ce6 (f) in PBS (λ_{ex} = 535 nm) upon irradiation by the 20 mW/cm^2 white light LED irradiation.

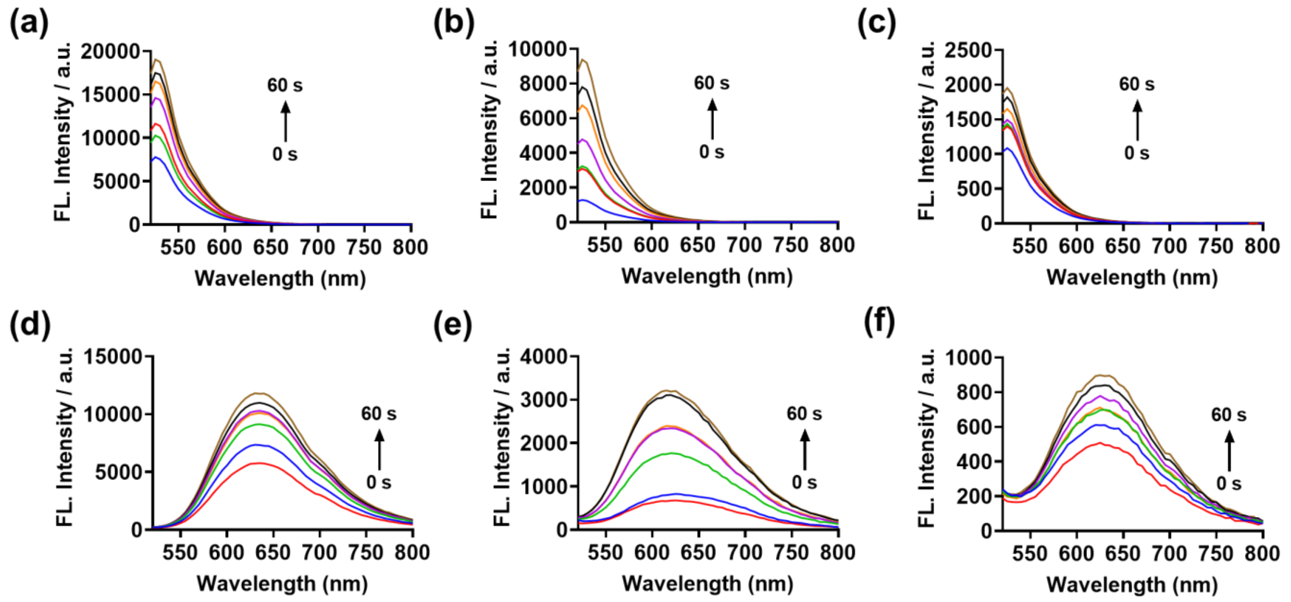

Figure S11. ROS generation of **TTMIndoOMe** (a), and commercial PS methylene blue (b) and 2% DMSO as co-solvent (c) in PBS (λ_{ex} = 480 nm) upon irradiation by the 20 mW/cm^2 white light LED irradiation under hypoxic (O_2% = 2%) condition. $O_2^{\bullet-}$ generation of of **TTMIndoOMe** (d), and commercial PS methylene blue (e) and 2% DMSO as co-solvent (f) in PBS (λ_{ex} = 535 nm) upon irradiation by the 20 mW/cm^2 white light LED irradiation under hypoxic (O_2% = 2%) condition.

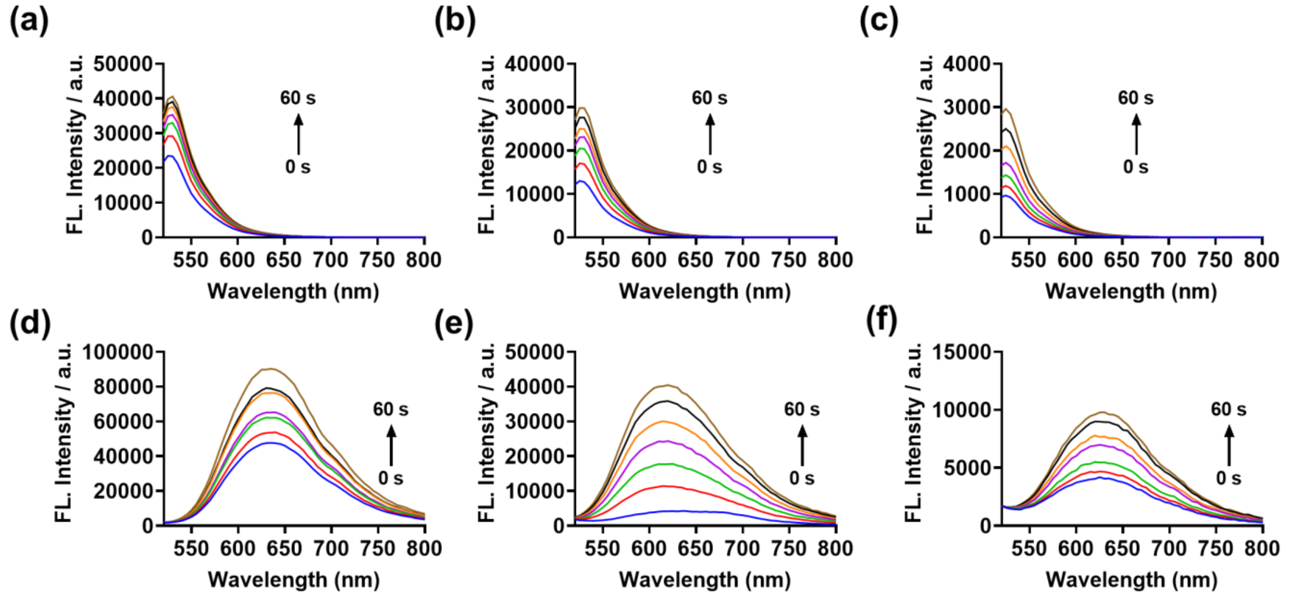

Figure S12. ROS generation of **TTMIndoOMe** (a), and commercial PS methylene blue (b) and 2% DMSO as co-solvent (c) in PBS (λ_{ex} = 480 nm) upon irradiation by the 20 mW/cm^2 white light LED irradiation ambient condition. $O_2^{\bullet-}$ generation of of **TTMIndoOMe** (d), and commercial PS methylene blue (e) and 2% DMSO as co-solvent (f) in PBS (λ_{ex} = 535 nm) upon irradiation by the 20 mW/cm^2 white light LED irradiation under ambient condition.

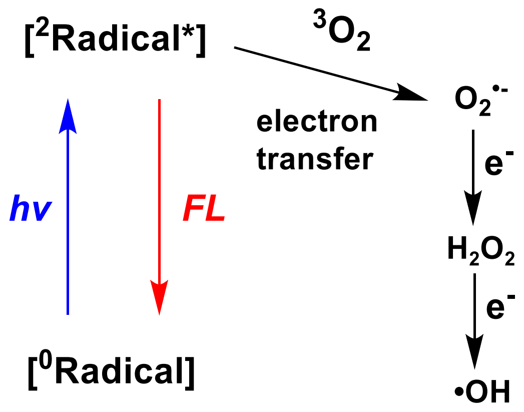

Figure S13. Proposed mechanism of TTMIndo π-radicals PS for ROS generation upon irradiation.

Cell Culture

The human cell lines HepG2, A549, and mice cell lines B16 used in our experiments were purchased from the Cell Resource Centre, Institute of Basic Medical Sciences, Chinese Academy of Medical Sciences & Peking Union Medical College. DC cells were generated from the bone marrow of 6-week-old C57BL/6 mice. HepG2 cells were cultured in DMEM (Dulbecco's modified Eagle's medium) containing 10% FBS and 1% penicillin-streptomycin under humidified atmosphere of 5% CO_2 at 37 ^oC under normoxic or hypoxic (O_2% = 2%) conditions. A549 cells were cultured in DMEM (Dulbecco's modified Eagle's medium) containing 10% FBS and 1% penicillin-streptomycin under humidified atmosphere of 5% CO_2 at 37 ^oC under normoxic condition. B16 cells were cultured in Roswell Park Memorial Institute (RPMI) 1640 containing 10% FBS and 1% penicillin-streptomycin under humidified atmosphere of 5% CO_2 at 37 ^oC under normoxic condition. The hypoxic environments *in vitro* experiments were constructed by SANYO O_2/CO_2 incubator MCO-5M, SANYO Electric Co., Ltd..

The Photostability Evaluation of TTMIndoOMe

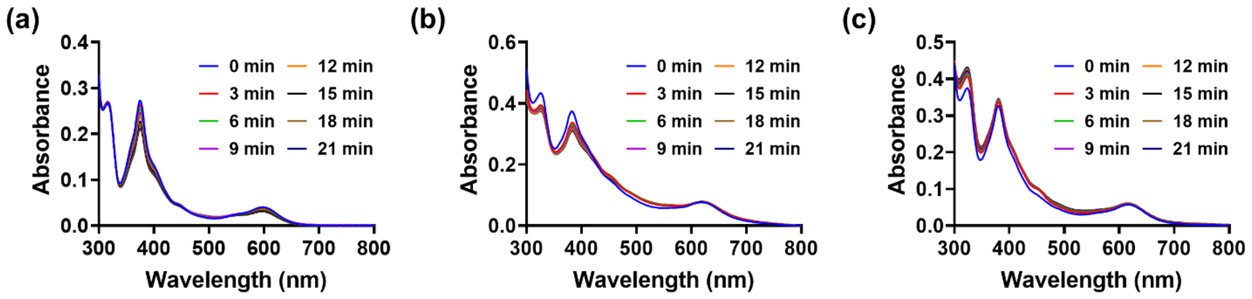

Figure S14. The time-dependent absorption spectra of 20 μM **TTMIndoOMe** compounds in (a) $CHCl_3$, (b) PBS and (c) water upon irradiation by a white light source (20 mW/cm^2).

The Stability Evaluation of TTMIndoOMe in Different Cell Lysates

In the cell lysate, T**TMIndoOMe** was diluted to 100 μM in the lysate of B16, A549, and HepG2 cells and incubated at 37 °C for the specified time intervals. After incubation, twice the volume of methanol was added to the lysate, and the supernatant was obtained through centrifugation at 12000 rpm for 30 min. The HPLC system was performed on a Thermo U3000, with chromatographic separation achieved on an InfinityLab Poroshell 120 EC-C18 column (150 mm × 4.6 mm, 4 μm particle size). A linear gradient from 50% B (0.1% formic acid in methanol) to 100% B (0.1% formic acid in methanol) in 5 min at a flow rate of 0.6 mL/min, and 100% B (0.1% formic acid in methanol) in 18 min at a flow rate of 0.6 mL/min with a detection wavelength of 600 nm was applied. The HPLC chromatogram profiles of **TTMIndoOMe**, recorded at various incubation times, showed no degraded peaks or significant changes in peak areas over time, indicating the excellent stability of **TTMIndoOMe** within B16, A549, and HepG2 tumor cells as time extended.

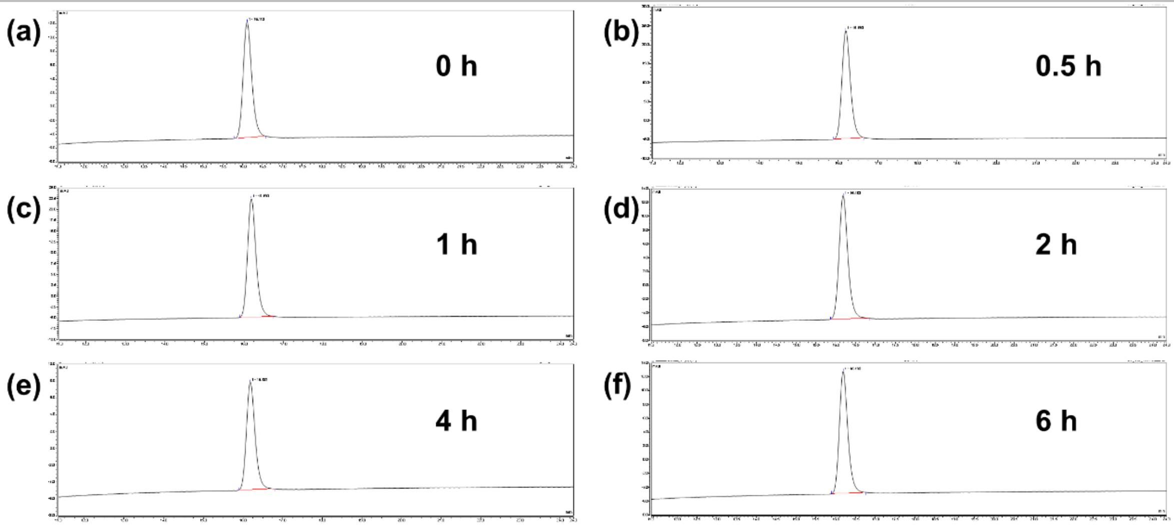

Figure S15. Stability study of **TTMIndoOMe** in the lysate of B16 cells at various time points (0 h, 0.5 h, 1 h, 2 h, 4 h, and 6 h) using HPLC analysis.

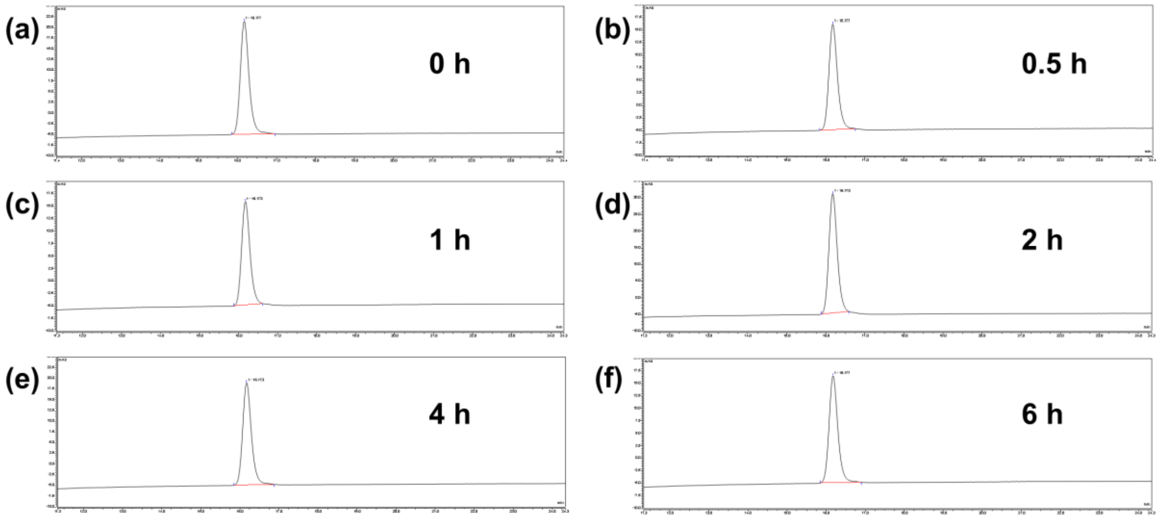

Figure S16. Stability study of **TTMIndoOMe** in the lysate of A549 cells at various time points (0 h, 0.5 h, 1 h, 2 h, 4 h, and 6 h) using HPLC analysis.

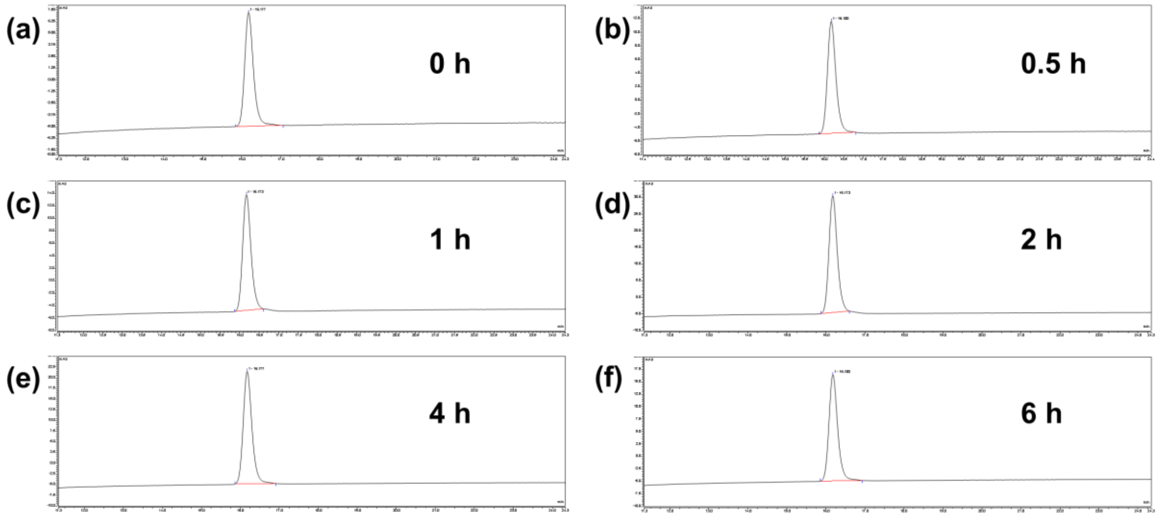

Figure S17. Stability study of **TTMIndoOMe** in the lysate of HepG2 cells at various time points (0 h, 0.5 h, 1 h, 2 h, 4 h, and 6 h) using HPLC analysis.

Dark and Light Cell Cytotoxicity of the TTMIndoOMe under Normoxic and Hypoxic (O_2% = 2%) Conditions

The dark and light cell cytotoxicity of **TTMIndoOMe** under normoxic and hypoxic (O_2% = 2%) conditions were determined by MTS assay *in vitro*. HepG2, A549, and B16cells were seeded in 96-well flat-bottom microtiter plates at a density of 1×10^4 cells/mL with 200 μL per well, incubated in a humidified atmosphere containing 5% CO_2 at 37 °C for 24 h under normoxic condition. HepG2 cells were incubated hypoxic (O_2% = 2%) condition with the same atmosphere for 24 h. Then exposed to different concentrations (0-20 μM) of **TTMIndoOMe** with or without white light (20 mW/cm^2) irradiation for 10 min in PBS, respectively, Then, incubated in a humidified atmosphere containing 5% CO_2 at 37 °C for 24 h under normoxic and hypoxic (O_2% = 2%) conditions for another 16 h. After treatment, 20 μL MTS solution was added to each well and continued to incubate for 4 h. After 4 h incubation at 37 °C, the absorbance was measured at 490 nm with Tecan Spark 10M Multimode Microplate Reader. The hypoxic (O_2% = 2%) environments *in vitro* experiments were constructed by SANYO O_2/CO_2 incubator MCO-5M, SANYO Electric Co., Ltd.. Cell viability was calculated according to the following formula: Cell viability (%) = $(A-A_0) / (A_s-A_0) \times100\%$, where A is the absorbance of the experimental group, A_s is the absorbance of the control group, and A_0 is the absorbance of the blank group (no cells). The experiment was repeated three times for each compound.

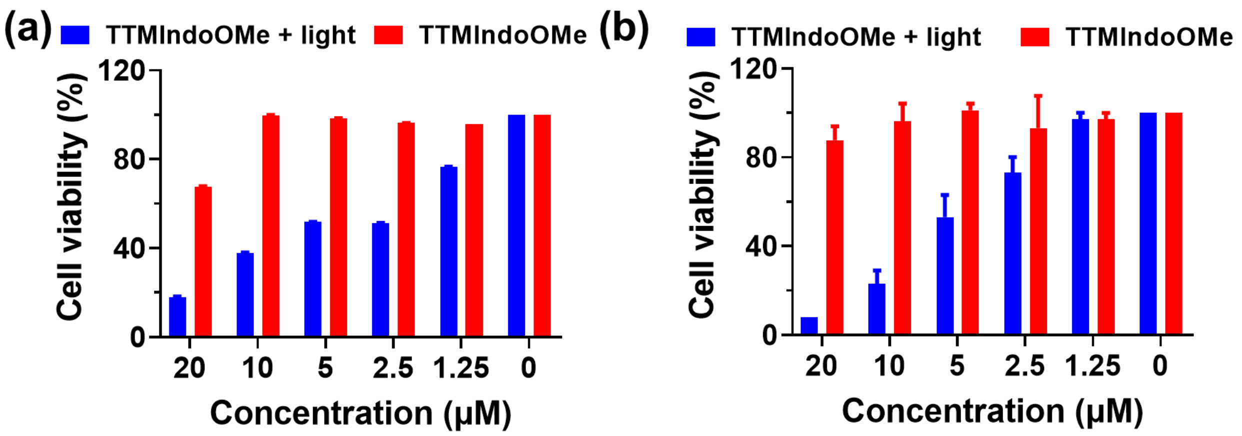

Figure S18. (a) B16 and (b) A549 cell cytotoxicity of the **TTMIndoOMe** in the dark (red) and upon white light irradiation (20 mW/cm^2, 10 min) (blue) under normoxic condition.

Confocal Fluorescence Images of HepG2 Cells Incubated with TTMIndoOMe under Normoxic and Hypoxic (O_2% = 2%) Conditions

Upon reaching 80% confluence, the HepG2 (300 μL, 4×10^4 cells/mL) was transferred into an 8-well chamber containing sterile coverslips at the bottom. After overnight culture at 37 °C under normoxic and hypoxic (O_2% = 2%) conditions, the medium was replaced with PBS, and HepG2 cells were incubated with 10 μM **TTMIndoOMe** for 1 h with or without irradiated by white light (20 mW/cm^2, 10 min), and then stained with 40 μM DCFH-DA, 20 μM DHE, 1 μg/mL PI and Hoechst 33324 (20 μg/mL) for 30 min. Then cells were washed with PBS at least three times. Fluorescence images were acquired by confocal microscopy Leica TCS SP8 X Confocal Microscope using 63× oil immersion objective with 4× magnification. The hypoxic environments *in vitro* experiments were constructed by SANYO O_2/CO_2 incubator MCO-5M, SANYO Electric Co., Ltd..

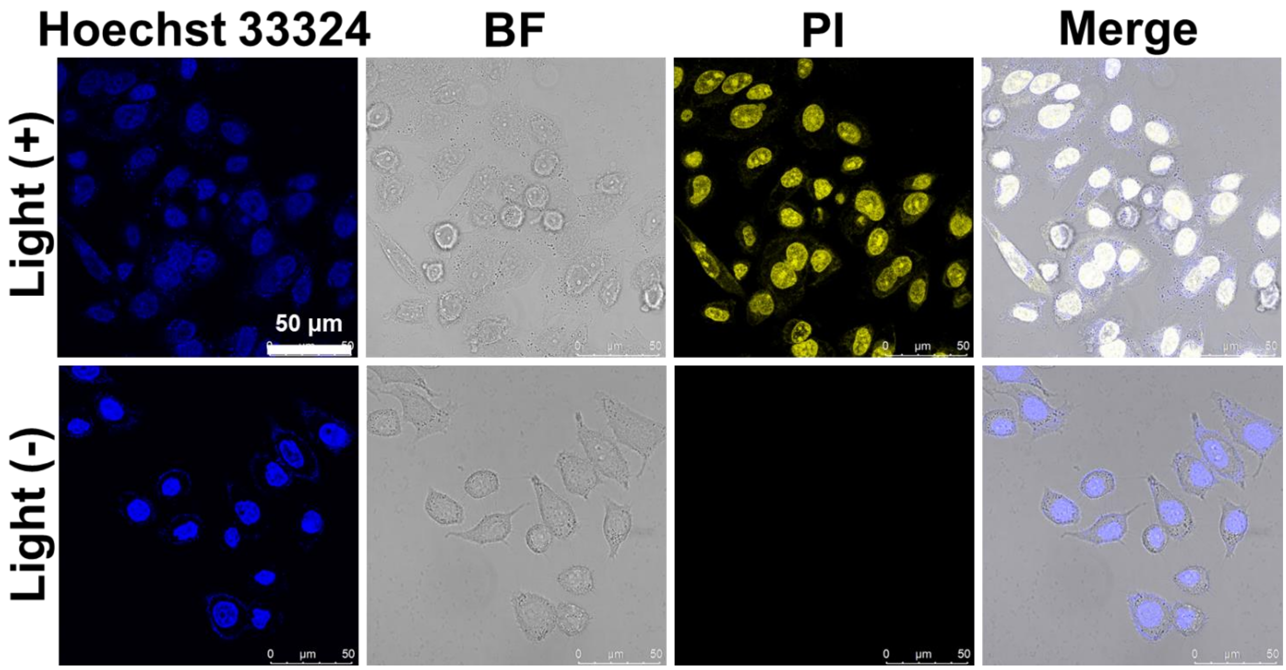

Figure S19. Confocal fluorescence images of HepG2 cells hypoxic (O_2% = 2%) condition. HepG2 cells were incubated with **TTMIndoOMe** (10 μM) for 1 h, irradiated by white light (20 mW/cm^2, 10 min), and then stained with 1 μg/mL PI and Hoechst 33324 (20 μg/mL) for 30 min. The cells were washed with PBS at least three times. Fluorescence images were acquired by confocal microscopy (PI, death cell marker, λ_{ex} = 488 nm, λ_{em} = 600-650 nm). The yellow fluorescence indicates HepG2 cell was killed by ROS generated by **TTMIndoOMe** upon light irradiation. Scale bar: 50 μm.

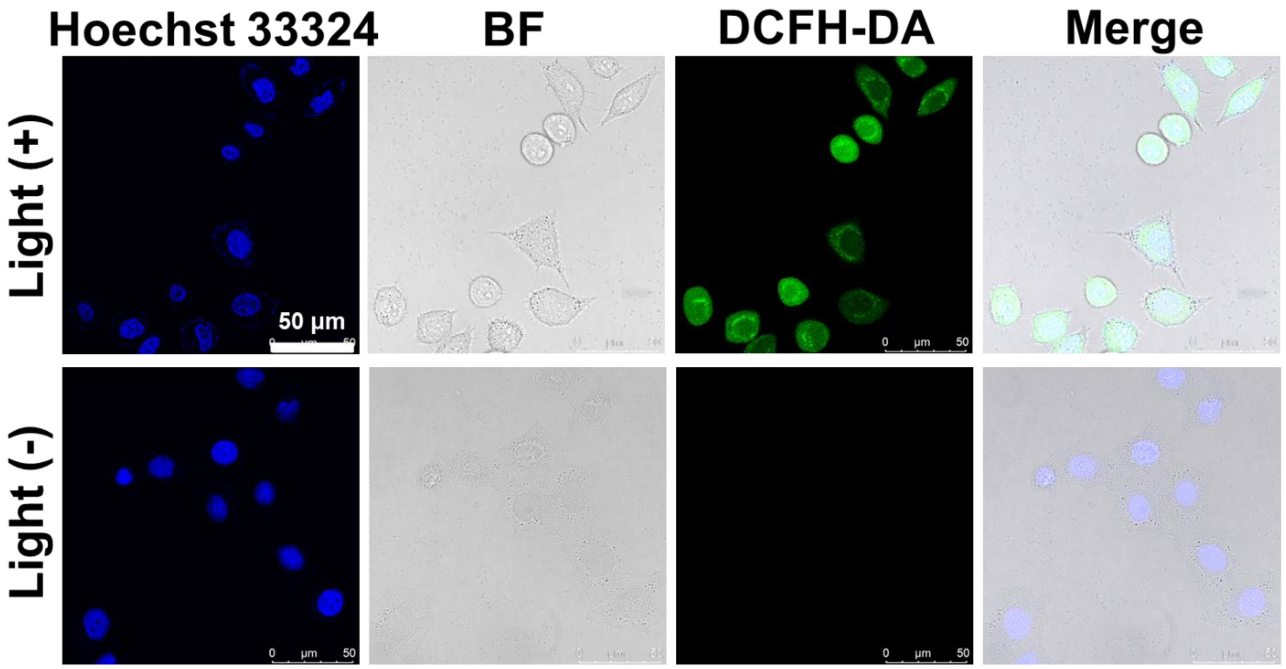

Figure S20. Confocal fluorescence images of HepG2 cells hypoxic (O_2% = 2%) condition. HepG2 cells were incubated with **TTMIndoOMe** (10 μM) for 1 h, irradiated by white light (20 mW/cm^2, 10 min), and then stained with 40 μM DCFH-DA and Hoechst 33324 (20 μg/mL) for 30 min. The cells were washed with PBS at least three times. Fluorescence images were acquired by confocal microscopy (DCFH-DA, ROS generation marker, λ_{ex} = 488 nm, λ_{em} = 495-550 nm). The green fluorescence indicates DCFH-DA is oxidized to DCF by ROS generated by **TTMIndoOMe** upon light irradiation. Scale bar: 50 μm.

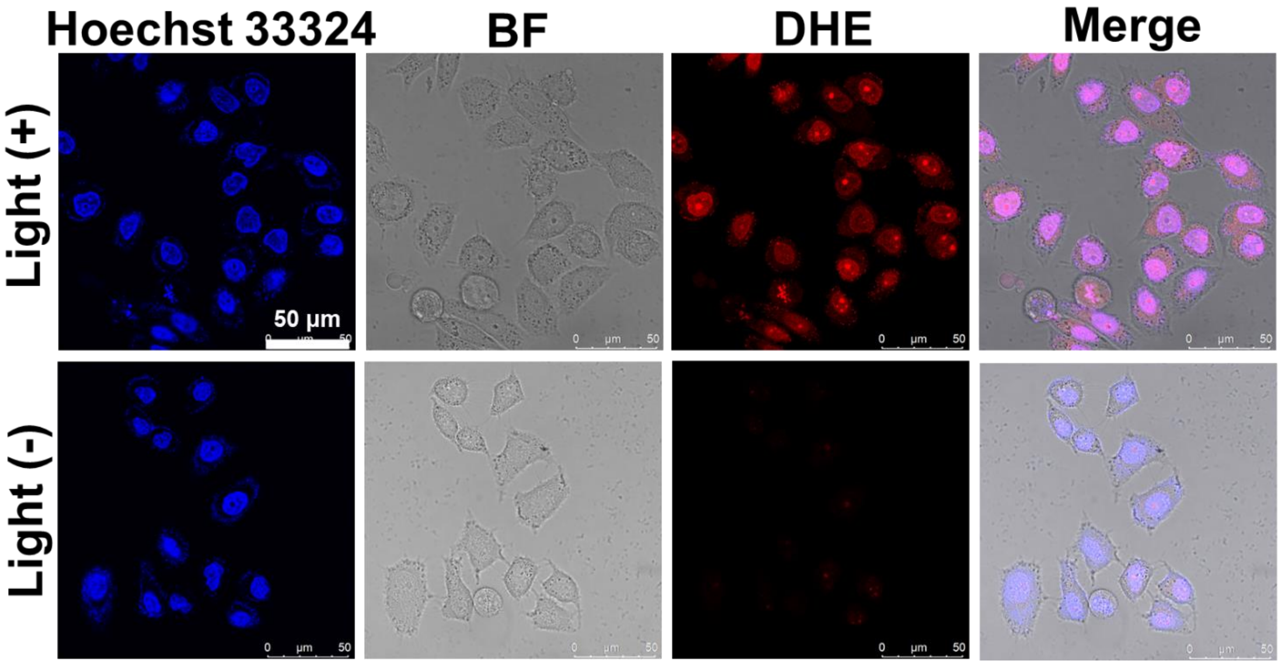

Figure S21. Confocal fluorescence images of HepG2 cells hypoxic (O_2% = 2%) condition. HepG2 cells were incubated with **TTMIndoOMe** (10 μM) for 1 h, irradiated by white light (20 mW/cm^2, 10 min), and then stained with 20 μM DHE and Hoechst 33324 (20 μg/mL) for 30 min. The cells were washed with PBS at least three times. Fluorescence images were acquired by confocal microscopy (DHE, $O_2^{\cdot-}$ generation marker, λ_{ex} = 535 nm, λ_{em} = 600-650 nm). Scale bar: 50 μm.

Animals and Tumor Models

Immunocompetent mice Balb/c nude (female) mice, 5 weeks of age, were purchased from Beijing HFK Bioscience Co. Ltd. and were housed in a specific pathogen-free room. For investigating the photodynamic therapeutic efficacy of the **TTMIndoOMe** *in vivo*, the antitumor effects were conducted on HepG2 tumor-bearing nude mice. The HepG2 tumor model was established by subcutaneous injection of HepG2 cells ($\sim 2 \times 10^6$) suspended in 50 μL Saline.

C57BL/6 (female) mice, 5 weeks of age, were purchased from Beijing HFK bioscience Co. Ltd. For investigating the photodynamic immunotherapeutic efficacy of the **TTMIndoOMe** *in vivo*, the antitumor effects were conducted on B16 tumor-bearing mice. The B16 tumor model was established by subcutaneous injection of B16 cells ($\sim 5 \times 10^5$) suspended in 50 μL Saline.

All animal experiments were approved by the Animal Care and Use Committee of the Institute of Materia Medica, Chinese Academy of Medical College (Approval No.00003996, 00004181). All cell lines were tested and confirmed to be free of Mycoplasma and other rodent pathogens by the China Centre for Type Culture Collection (CCTCC). No other authentication assay was performed.

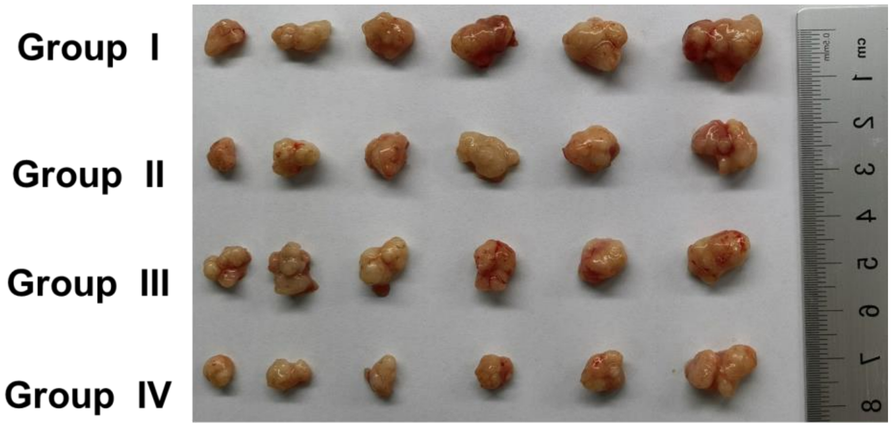

Figure S22. Representative photograph of HepG2 tumors collected from all groups on the 18th day post-treatment.

Measurement of TNF-α, IFN-γ, IL-12p70 and IL-6

BMDCs were generated from the bone marrow of 6-week-old C57/BL6 mice according to previously described methods [41,42]. Briefly, B16 cells were seeded in the 24-well plate at a density of 2×10^5 cells/well for 12 h and pretreated with **TTMIndoOMe**. Then, the cells were treated with PBS and co-incubated for 1 h, and treated with white light irradiation (20 mW/cm^2) for 10 min. Afterward 5×10^5 immature DC cells with fresh medium were co-cultured with pretreated B16 cells for 24 h. The culture medium was collected, and the expression levels of TNF-α, IFN-γ, IL-12p70, and IL-6 were determined by enzyme-linked immunosorbent assay (ELISA), according to the manufacturer's instruction.

DCs Maturation *in vitro*

BMDCs were generated from the bone marrow of 6-week-old C57/BL6 mice. B16 cells were seeded in the 24-well plate at a density of 2×10^5 cells/well for 12 h and pretreated with **TTMIndoOMe**. Then, the cells were treated with PBS and co-incubated for 1 h, and treated with white light irradiation (20 mW/cm^2) for 10 min. Afterward 5×10^5 immature DC cells with fresh medium were co-cultured with pretreated B16 cells for 24 h. Then, staining with anti-CD11c-APC, anti-MHCII-percpcy5.5, and anti-CD86-PE antibodies, the maturation of DC cells was examined by flow cytometry measurement.

Immunogenic Cell Death Induction of TTMIndoOMe *in vitro*

Immunofluorescence detected Calreticulin (CRT) expression on the cell's surface. For immunofluorescence analysis, B16, A549, and HepG2 cells were seeded in the 8-well chamber containing sterile coverslips at the bottom at a density of 2×10^5 cells/well and incubated at 37 °C for 24 h. After the cells were incubated with **TTMIndoOMe** in PBS for 1h, the cells were treated with white light irradiation (20 mW/cm^2) for 10 min, and 4 h later the cells were washed twice with cold PBS and fixed in 0.25% paraformaldehyde for 5 min. The cells were then incubated with the primary antibody of CRT after washing twice in cold PBS. The cells were washed again for 30 min and incubated with the FITC-conjugated monoclonal secondary antibody for 30 min. The cells were eventually stained with DAPI and examined by CLSM (LSM880, Zeiss).

HMGB1 and extracellular ATP secretion were tested using an ELISA assay kit. Typically, B16, A549, and HepG2 cells were seeded in the 24-well plate at a density of 2×10^5 cells/well and incubated at 37 °C for 24 h. After the cells were incubated with **TTMIndoOMe** in PBS for 1h, the cells were treated with white light irradiation (20 mW/cm^2) for 10 min. the fresh medium was added and the cell culture supernatant was collected after 24 h incubation, and then the HMGB1 and ATP secretion were examined using an ELISA assay kit according to the manufacturer's instructions.

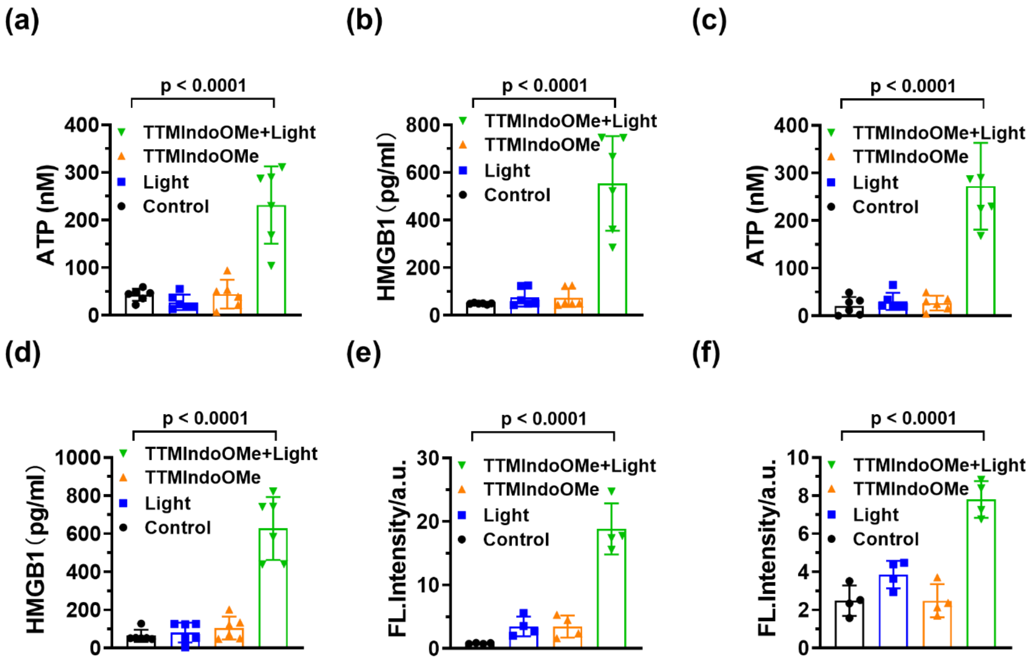

Figure S23. Characterizations of **TTMIndoOMe** plus light-treated A549 and HepG2 tumor cells *in vitro*. (a) ATP excretion (nM) and (b) HMGB1 release (pg/ml) from A549 cells upon indicated treatments (n = 6). (c) ATP secretion (nM) and (d) HMGB1 release (pg/ml) from HepG2 cells upon indicated treatments. Data are given as the mean ± SD (n = 6). FL Intensity of CRT exposure on the membrane of A549 (e) and HepG2 (f) cells upon indicated treatment capture by confocal fluorescence microscope. Data are given as the mean ± SD (n = 4).

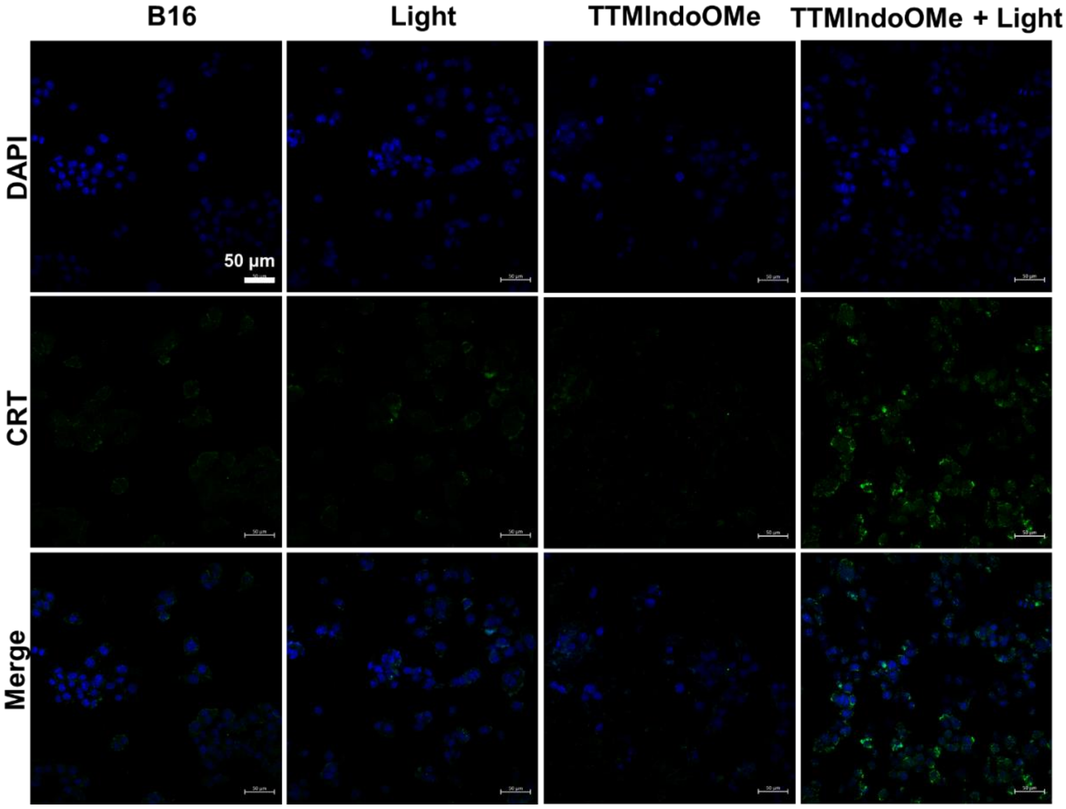

Figure S24. Confocal fluorescence images of calreticulin (CRT) expression of B16 cells. The fluorescence images were acquired by confocal microscopy. The green fluorescence (CRT, λ_{ex} = 488 nm, λ_{em} = 500-540 nm). The bule fluorescence (DAPI, λ_{ex} = 405 nm, λ_{em} = 450-490 nm). The green fluorescence indicates calreticulin (CRT) expression was detected and **TTMIndoOMe** could induce the ICD of B16 cells upon light irradiation. Scale bar: 50 μm.

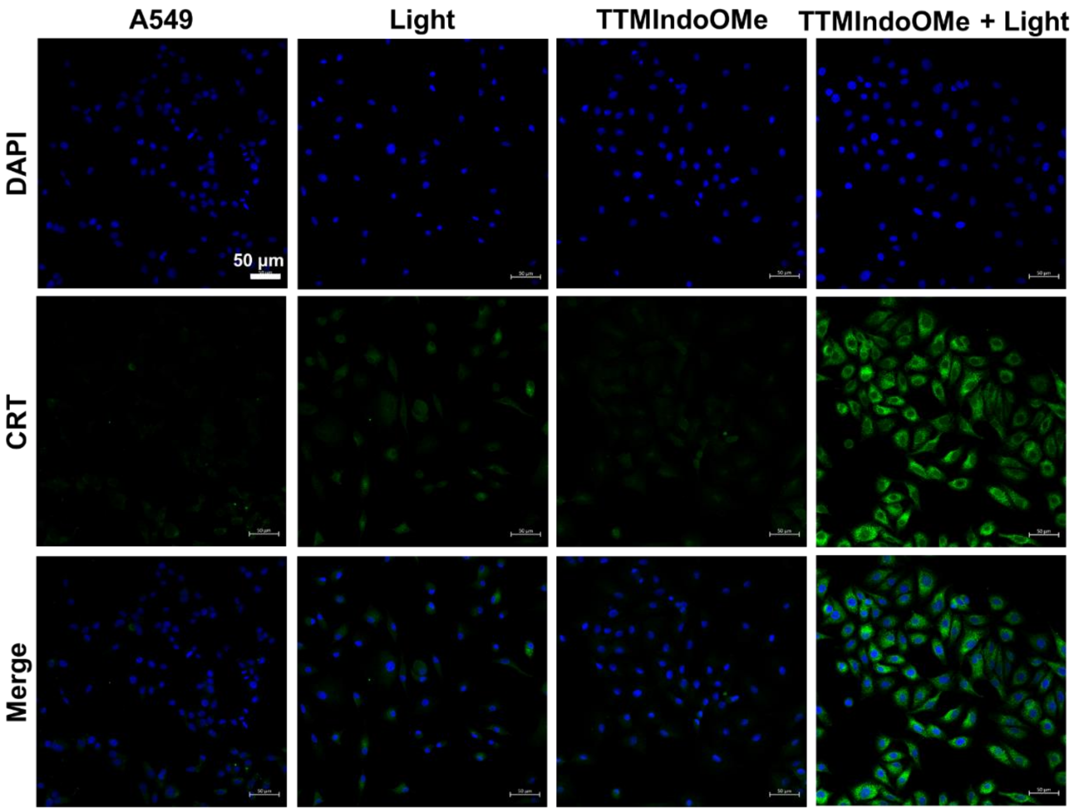

Figure S25. Confocal fluorescence images of calreticulin (CRT) expression of A549 cells. The fluorescence images were acquired by confocal microscopy. The green fluorescence (CRT, λ_{ex} = 488 nm, λ_{em} = 500-540 nm). The bule fluorescence (DAPI, λ_{ex} = 405 nm, λ_{em} = 450-490 nm). The green fluorescence indicates calreticulin (CRT) expression was detected and **TTMIndoOMe** could induce the ICD of A549 cells upon light irradiation. Scale bar: 50 μm.

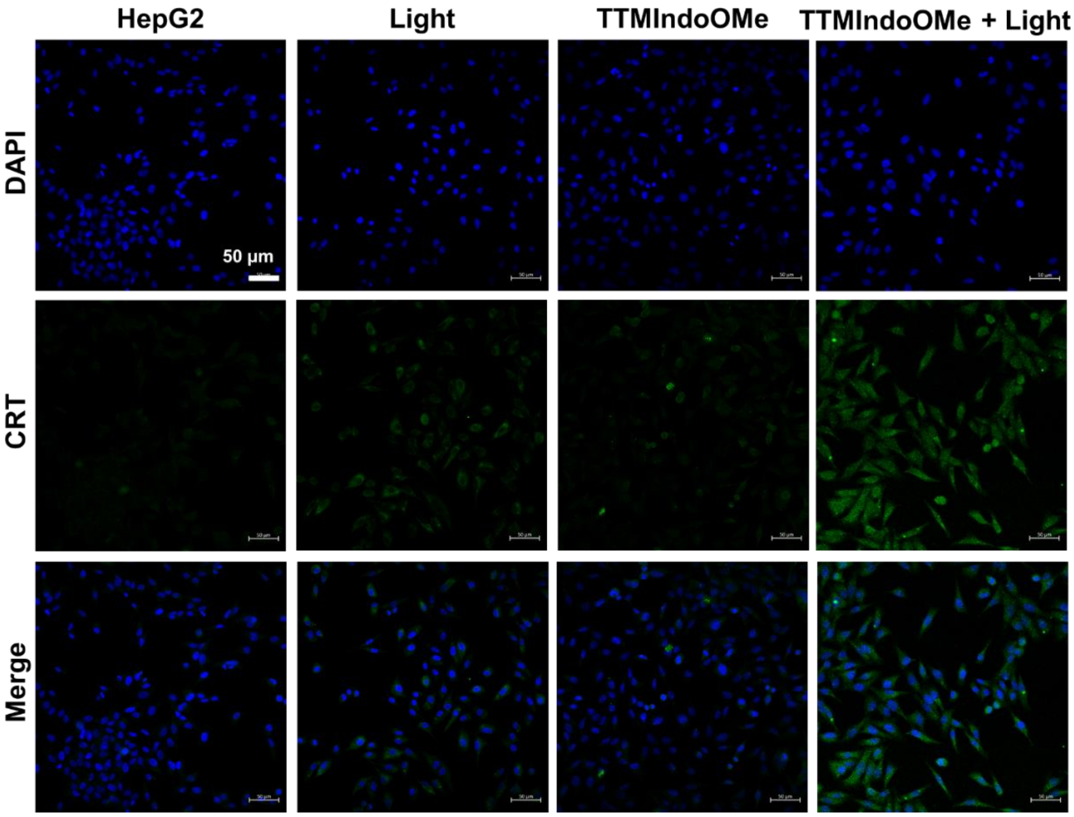

Figure S26. Confocal fluorescence images of calreticulin (CRT) expression of HepG2 cells. The fluorescence images were acquired by confocal microscopy. The green fluorescence (CRT, λ_{ex} = 488 nm, λ_{em} = 500-540 nm). The bule fluorescence (DAPI, λ_{ex} = 405 nm, λ_{em} = 450-490 nm). The green fluorescence indicates calreticulin (CRT) expression was detected and **TTMIndoOMe** could induce the ICD of HepG2 cells upon light irradiation. Scale bar: 50 μm.

Anti-Tumor Effect of TTMIndoOMe *in vivo*

The photodynamic therapy effect of **TTMIndoOMe** was performed in HepG2-bearing nude mice. HepG2-bearing nude mice were randomly divided into three groups when the tumor volume reached around 100 mm^3. The mice were treated with I: PBS(PBS), II: only Light, III: only **TTMIndoOMe** (**TTMIndoOMe**), IV: **TTMIndoOMe** plus light (**TTMIndoOMe** + Light), respectively. **TTMIndoOMe** was given at an equal dose of 100 µL, 20 mg/kg by subcutaneous injections at 7, 9, 11, 13, 15, 17, 19, 21, 23 and 25 day. After that, the tumors were treated with white light irradiation (20 mW/cm^2) for 15 min. The mice were anesthetized using isoflurane in the chamber, RWD small animal anesthesia machine. The white LED light were located about 2 cm below the chamber. Both mouse body weights and tumor volumes were recorded every two days during the whole treatment. The length (L) and width (W) of each tumor were measured with a caliper, and the tumor volume was calculated with the formula of $V = (L \times W^2) / 2$.

The photodynamic immunotherapy effect of **TTMIndoOMe** was performed in B16-bearing C57/BL6 mice. B16-bearing mice were randomly divided into four groups when the tumor volume reached around 100 mm^3. The mice were treated with I: PBS(PBS), II: only white light irradiation (Light), III: only **TTMIndoOMe** (**TTMIndoOMe**), IV: **TTMIndoOMe** plus white light irradiation (**TTMIndoOMe**+Light), respectively. **TTMIndoOMe** was given at an equal dose of 100 µL, 15 mg/kg by subcutaneous injections at 6, 8, 10, 12, and 14 day. After that, the tumors were treated with white light irradiation (20 mW/cm^2) for 15 min. The mice were anesthetized using isoflurane in the chamber, RWD small animal anesthesia machine. The white LED light were located about 2 cm below the chamber. Both mouse body weights and tumor volumes were recorded every two days during the whole treatment. The length (L) and width (W) of each tumor were measured with a caliper, and the tumor volume was calculated with the formula of $V = (L \times W^2) / 2$. After 8 days of treatment, the mice were sacrificed and tumor tissue, spleen, and lymph nodes were harvested. The tumor tissue was taken for hematoxylin and eosin (H&E) and TUNEL (TdT-mediateddUTPNick-EndLabeling) stain. The spleen and lymph node were collected for flow cytometric analysis.

Evaluation of the Safety of TTMIndoOMe *in vivo*

To further assess the biosafety of **TTMIndoOMe** *in vivo*, the blood samples were collected from tumor models with **TTMIndoOMe** photodynamic immunotherapy and healthy mice for hematology markers evaluation, mainly including white blood cells (WBC), red blood cells (RBC), hemoglobin (HGB), mean corpuscular volume (MCV), mean corpuscular volume (MCH), platelets (PLT), mean corpuscular hemoglobin concentration (MCHC), basophilic leukemia (Bas), lymphocyte (Lym), neutrophil (Neu), monocytes (Mon), and eosinophil (Eos).

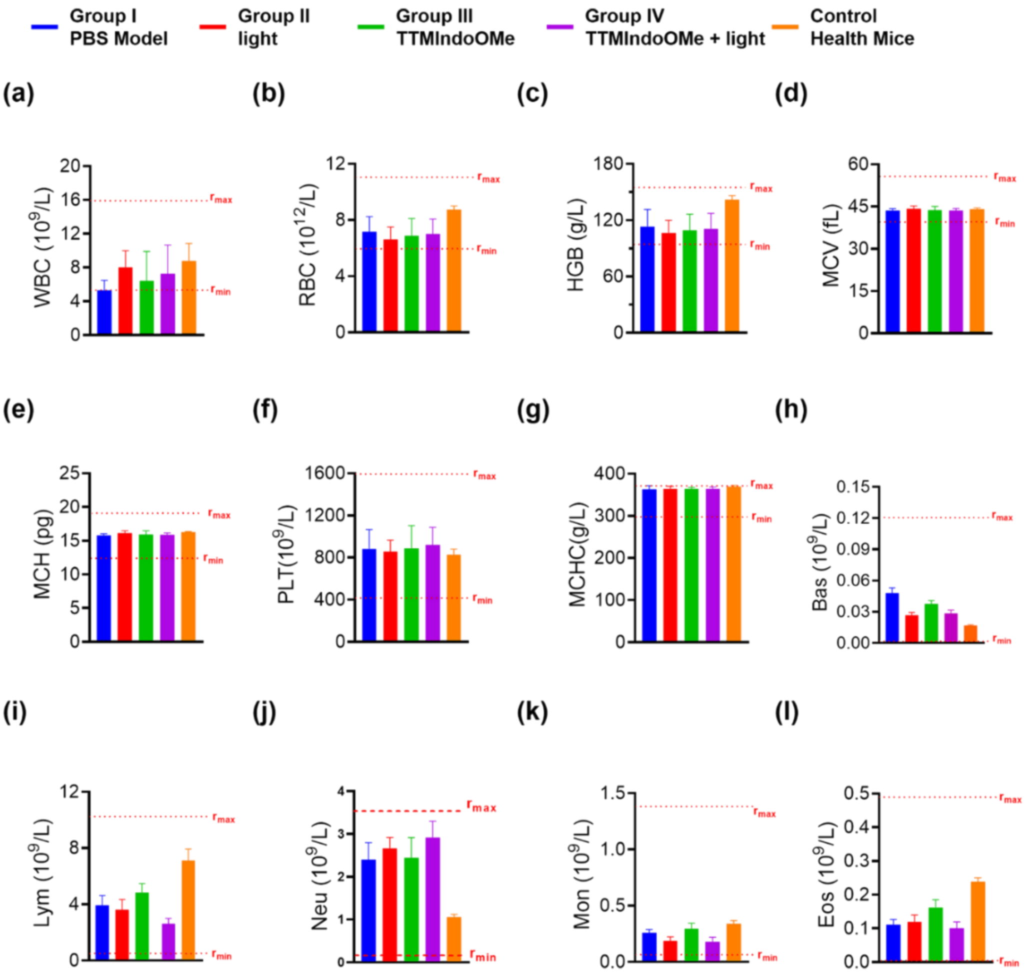

Figure S27. Investigation of the biosafety of **TTMIndoOMe** in B16 tumor-bearing mice model and healthy mice after photodynamic immunotherapy. Blue: PBS; Red: Light; Green: **TTMIndoOMe**; Purple: **TTMIndoOMe** + Light; Orange: Healthy mice. Abbreviations: white blood cells (WBC) (a), red blood cells (RBC) (b), hemoglobin (HGB) (c), mean corpuscular volume (MCV) (d), mean corpuscular volume (MCH) (e), platelets (PLT) (f), mean corpuscular hemoglobin concentration (MCHC) (g), basophilic leukemia (Bas) (h), lymphocyte (Lym) (i), neutrophil (Neu) (j), monocytes (Mon) (k), eosinophil (Eos) (l). Red short dotted lines indicate the range of normal reference values ($r_{min} \rightarrow r_{max}$) for BALB/c mice. Values are mean ± SEM (n = 6).

Antitumor Immune Response *in vivo*

Tumor-draining lymph nodes in each treatment group were obtained, then gently ground with rubbers of syringes and filtered to acquire single-cell suspension. Next, samples were centrifuged, and washed with PBS for 3 times followed by staining with Then, staining with anti-CD11c-APC, anti-MHCII-percpcy5.5, and anti-CD86-PE antibodies, the maturation of DC cells was examined by flow cytometry measurement.

Spleens in each treatment group were obtained, then gently ground with rubbers of syringes and filtered to acquire single-cell suspensions. Lysed with erythrocyte, and washed, single-cell suspensions of spleens in each treatment group were obtained. The percentages of $CD3^+CD8^+$ T cells, and $CD44^+CD62L^+$ T memory cells were analyzed using flow cytometry. For intracellular staining, cells were fixed, permeabilized, and washed according to the manufacturer's protocol. The percentages of $CD4^+CD25^+Foxp3^+$ Treg cells were analyzed using flow cytometry.

Tumor tissues were also used for IF and IHC analysis for characterizing $CD8^+$ T cells and IFN-γ, H&E staining, and TUNEL staining.

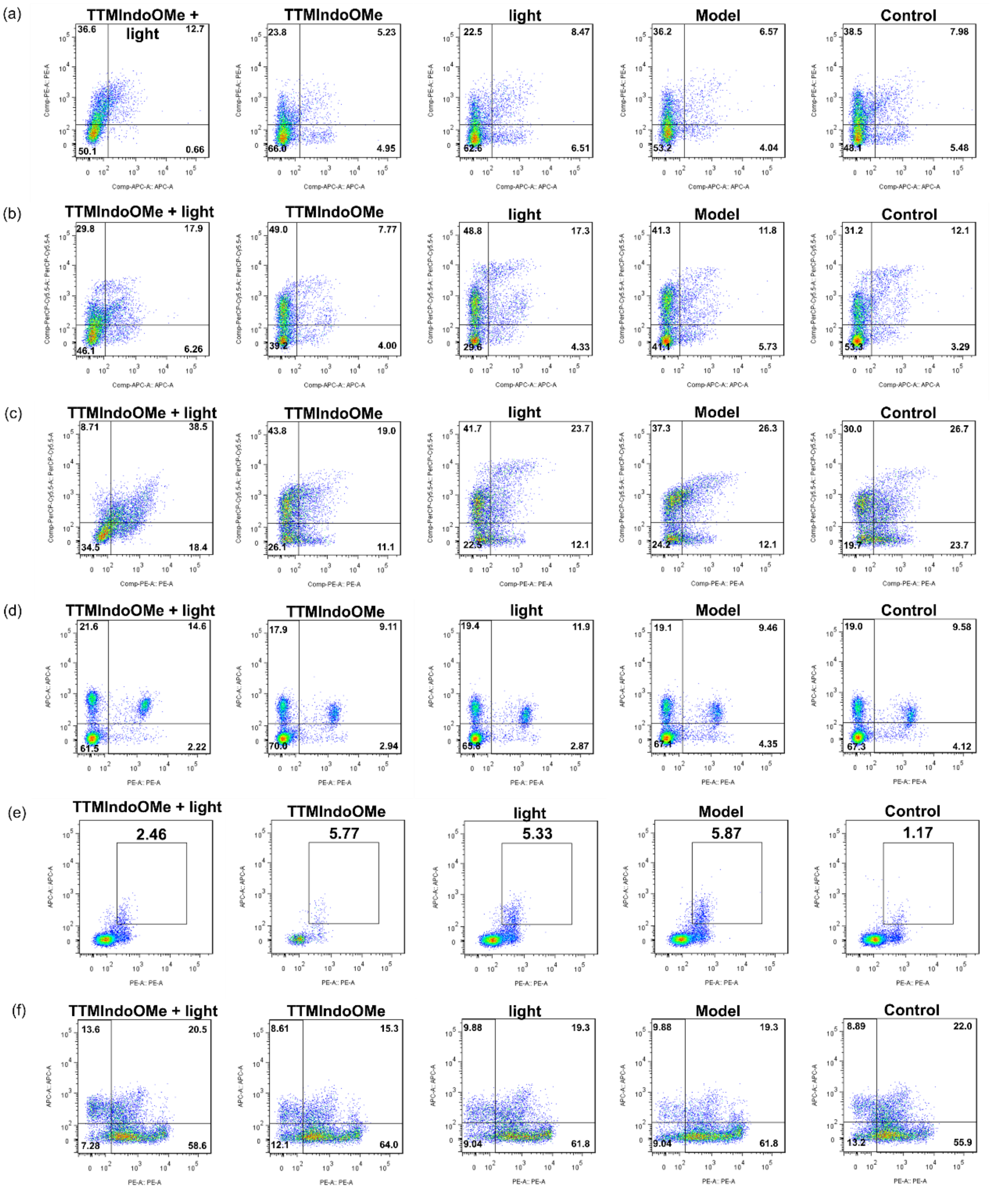

Figure S28. Flow cytometric analysis of immune responses in tumor-bearing mice after indicated treatments. (a) Flow cytometric analysis of CD11c^{+}CD86^{+} DCs cells, (b) CD11c^{+}MHCII^{+} DCs cells, and (c) CD86^{+} MHCII^{+} DCs cells in the LNs of tumor-bearing mice after indicated treatments. (d) Flow cytometric analysis of CD3^{+}CD8^{+} T cells in tumor-bearing mice after indicated treatments. (e) Flow cytometric analysis of the Tregs (CD4^{+}CD25^{+}Foxp3^{+} T cells) in tumor-bearing mice. (f) Flow cytometric analysis of CD44^{+}CD62L^{+} T cells in the spleen of tumor-bearing mice after indicated treatments.Data are given as the mean ± SEM (n = 6). *in vivo.*

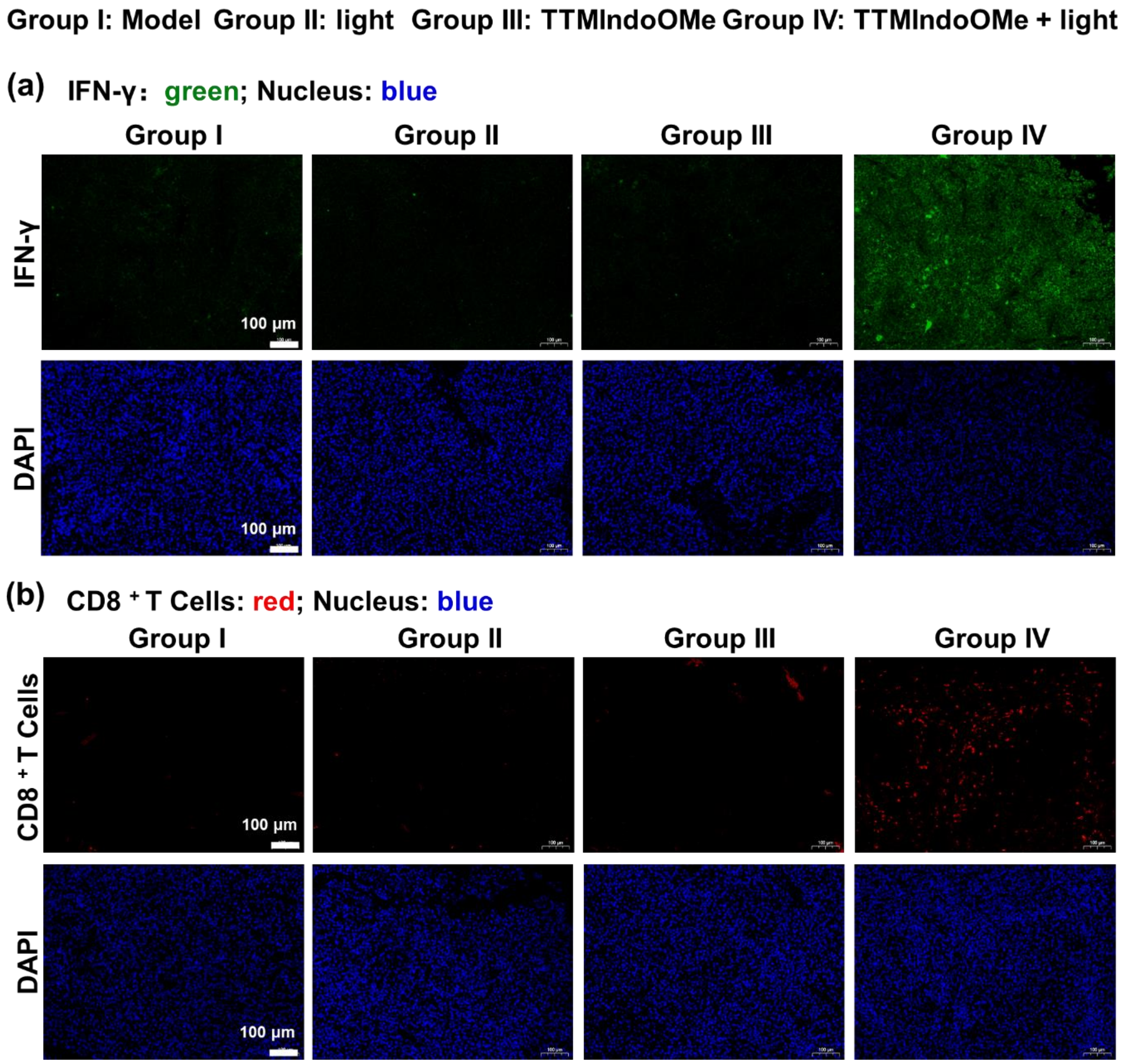

Figure S29. Immunofluorescence staining of IFN-γ (a) and $CD8^+$ T cells (b) in the tumor tissues after indicated treatments. Scale bars: 100 μm.

Statistical Analysis

Statistical analysis was implemented using Student's t-test or one-way ANOVA. All the results were analyzed with the use of Prism 7 (Prism GraphPad Software, Inc., San Diego). Analysis of flow cytometry data was performed by FlowJo 7.6.1 software (BD Biosciences, New York, USA). Data were expressed as mean ± SD, mean ± SEM.

NMR Spectra

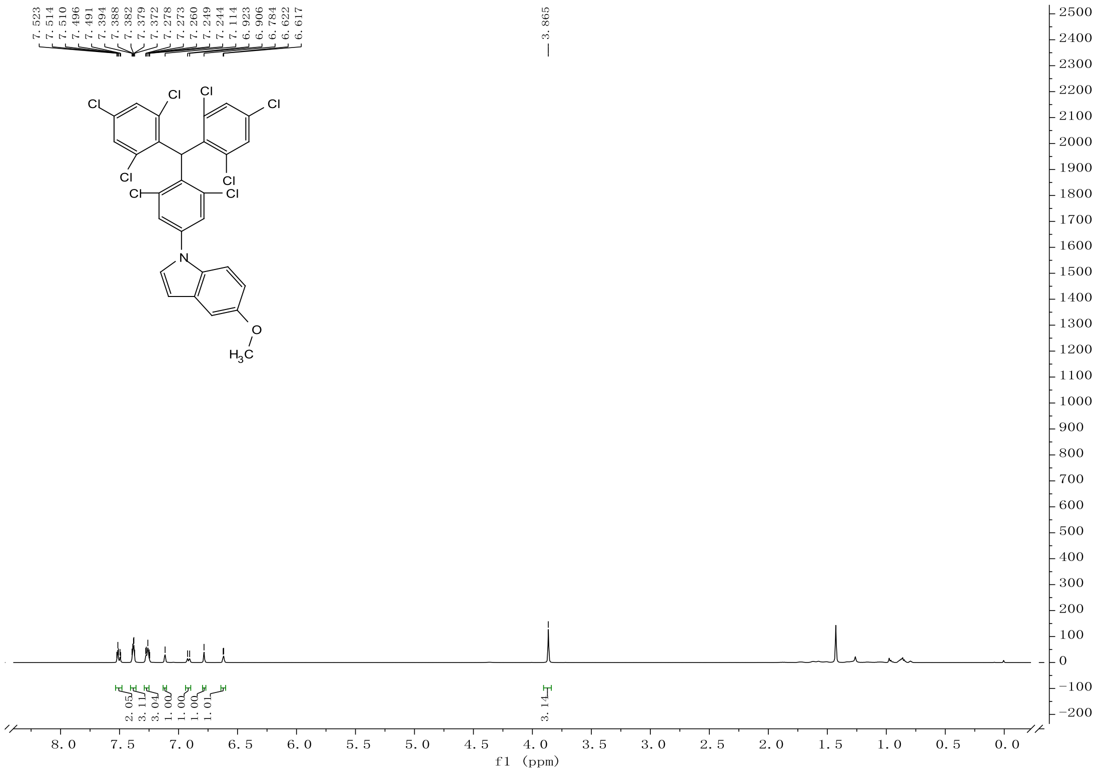

Figure S30. ^{1}H NMR (400 MHz, $CDCl_3$) of **TTMIndoOMe-1.**

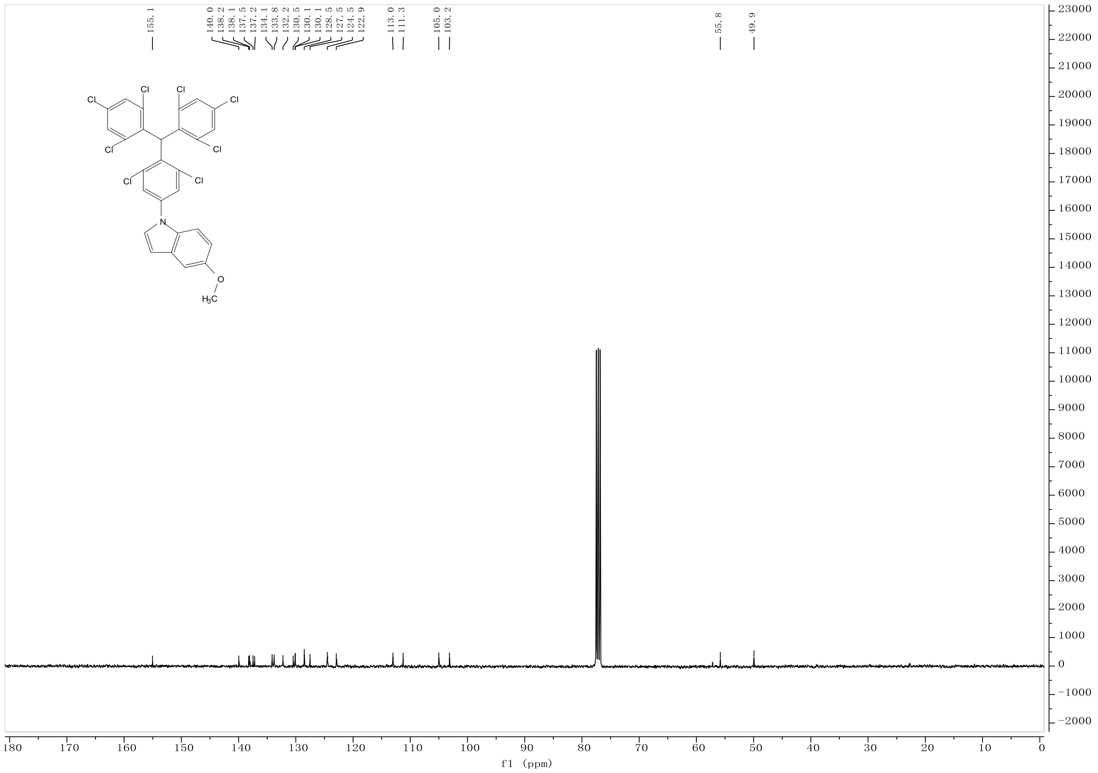

Figure S31. ^{13}C NMR (100 MHz, $CDCl_3$) of **TTMIndoOMe-1**.

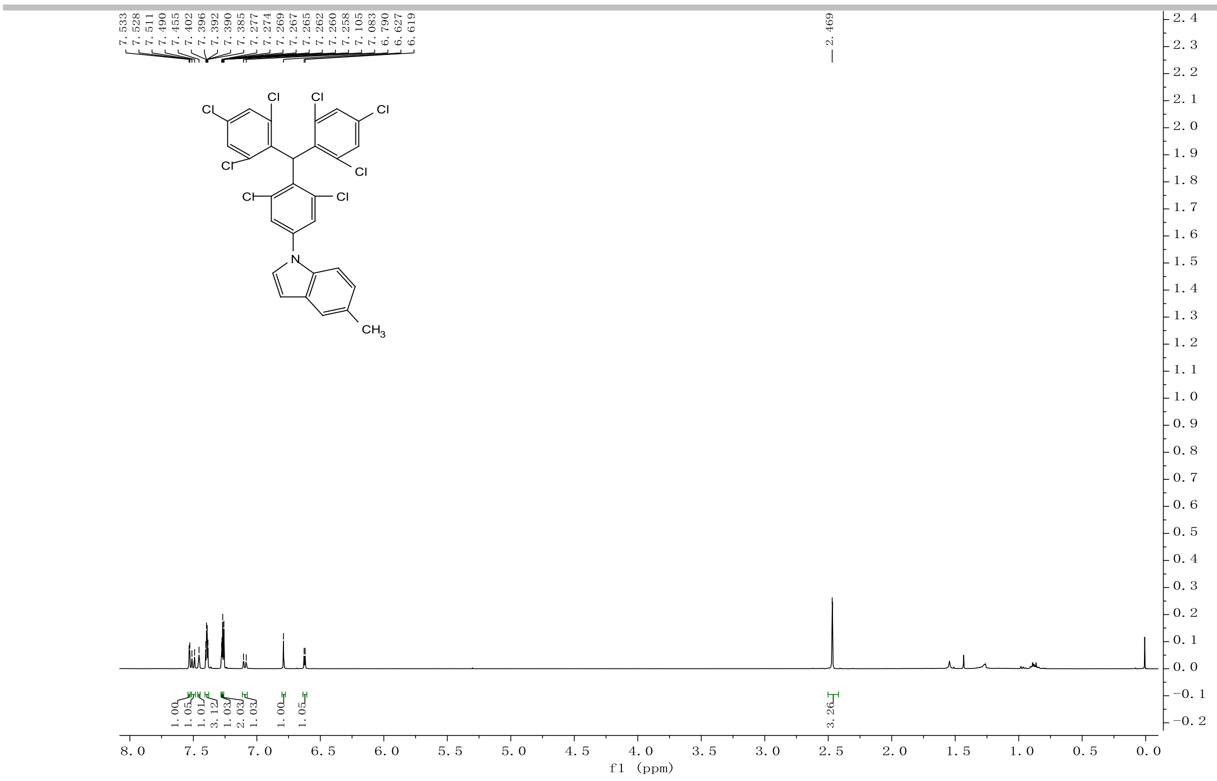

Figure S32. ^{1}H NMR (400 MHz, $CDCl_3$) of **TTMIndoMe-1**.

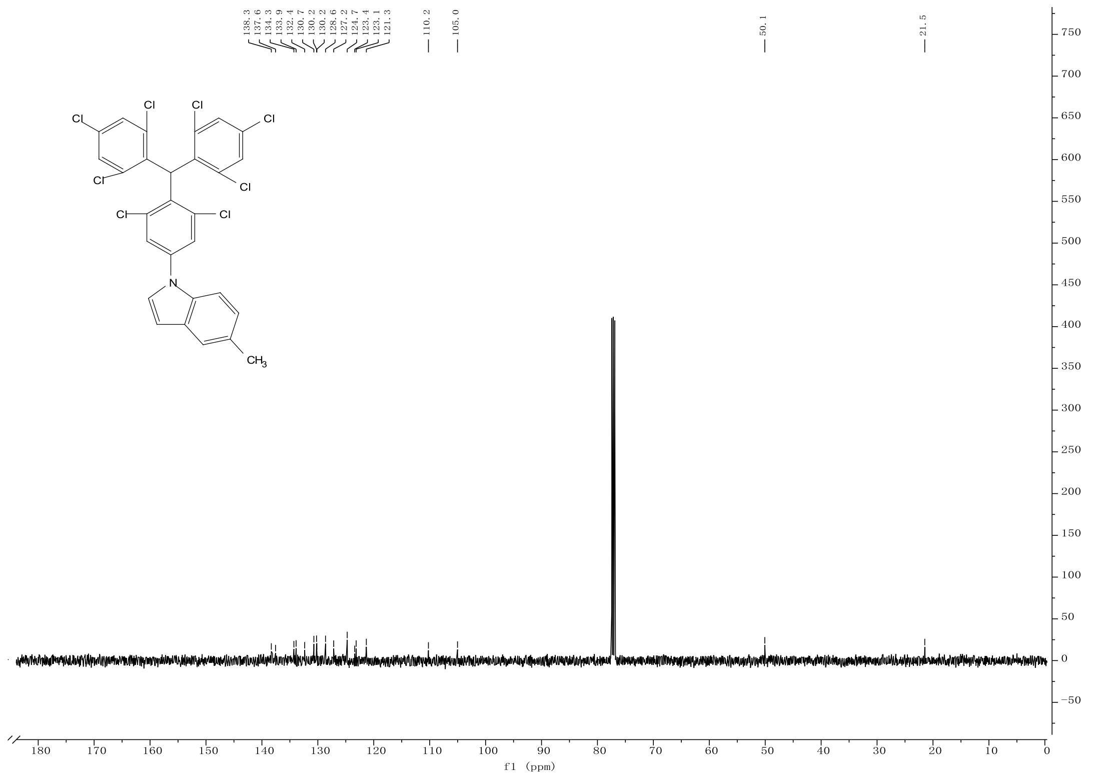

Figure S33. ^{13}C NMR (100 MHz, $CDCl_3$) of **TTMIndoMe-1**.

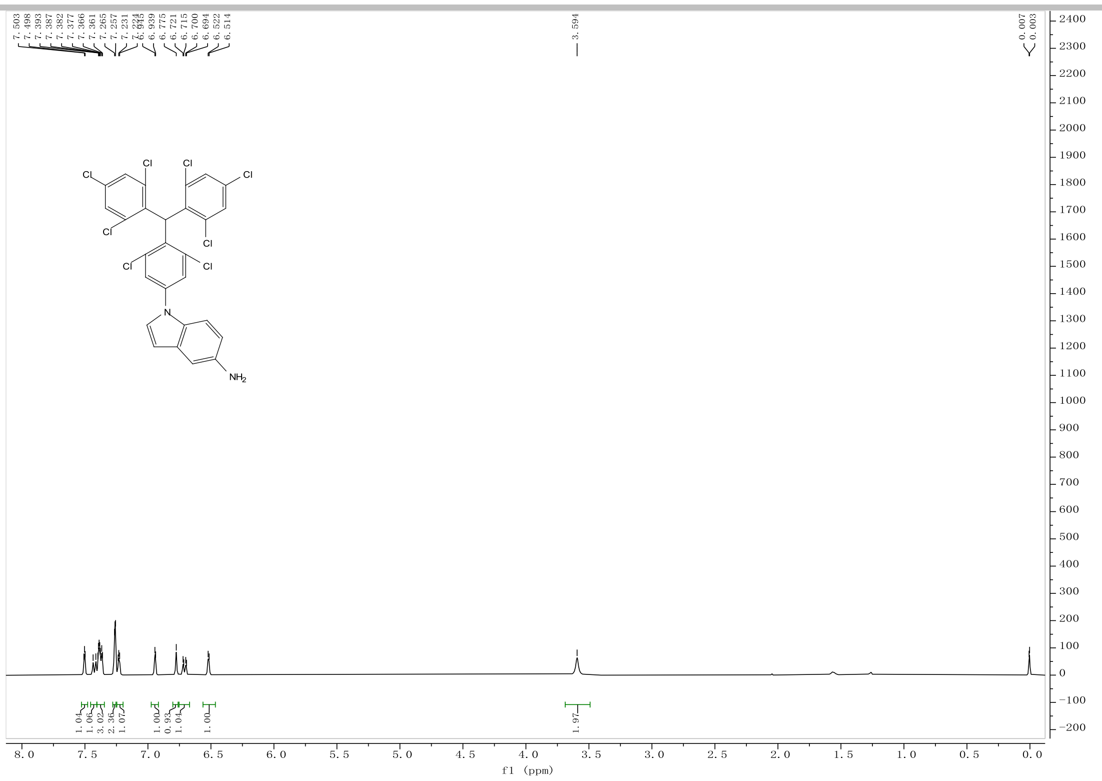

Figure S34. ^{1}H NMR (400 MHz, $CDCl_3$) of **TTMIndoNH₂-1**.

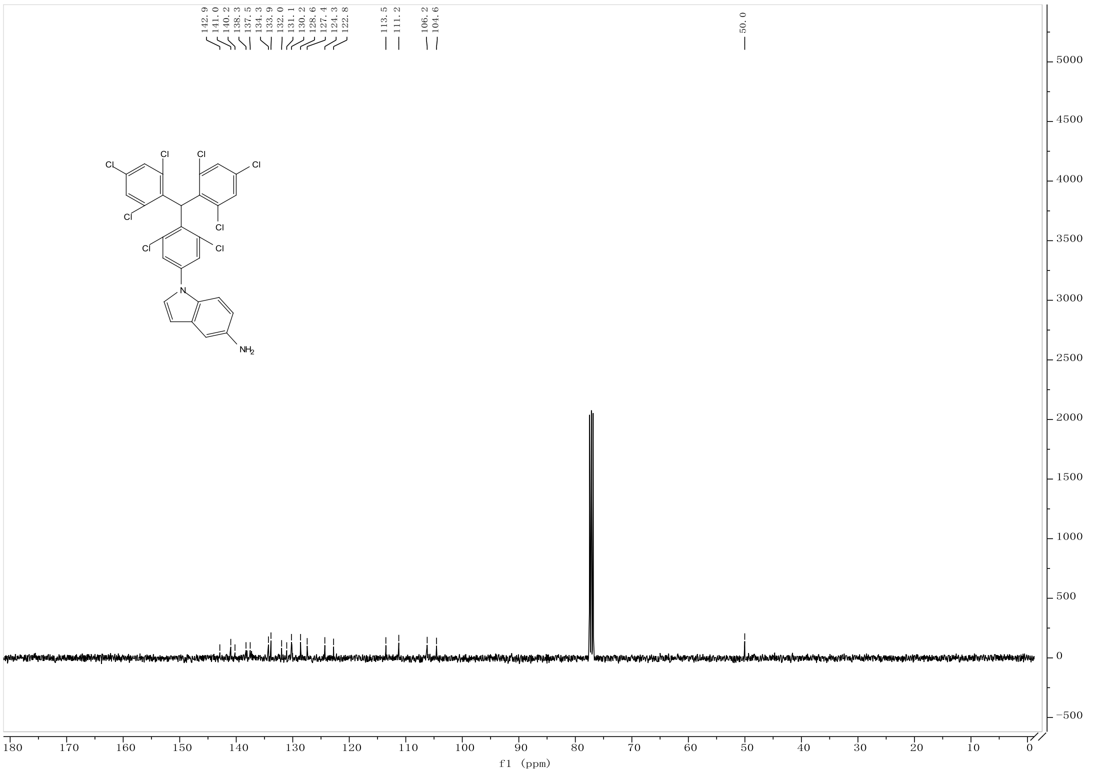

Figure S35. ^{13}C NMR (100 MHz, $CDCl_3$) of **TTMIndoNH₂-1**.

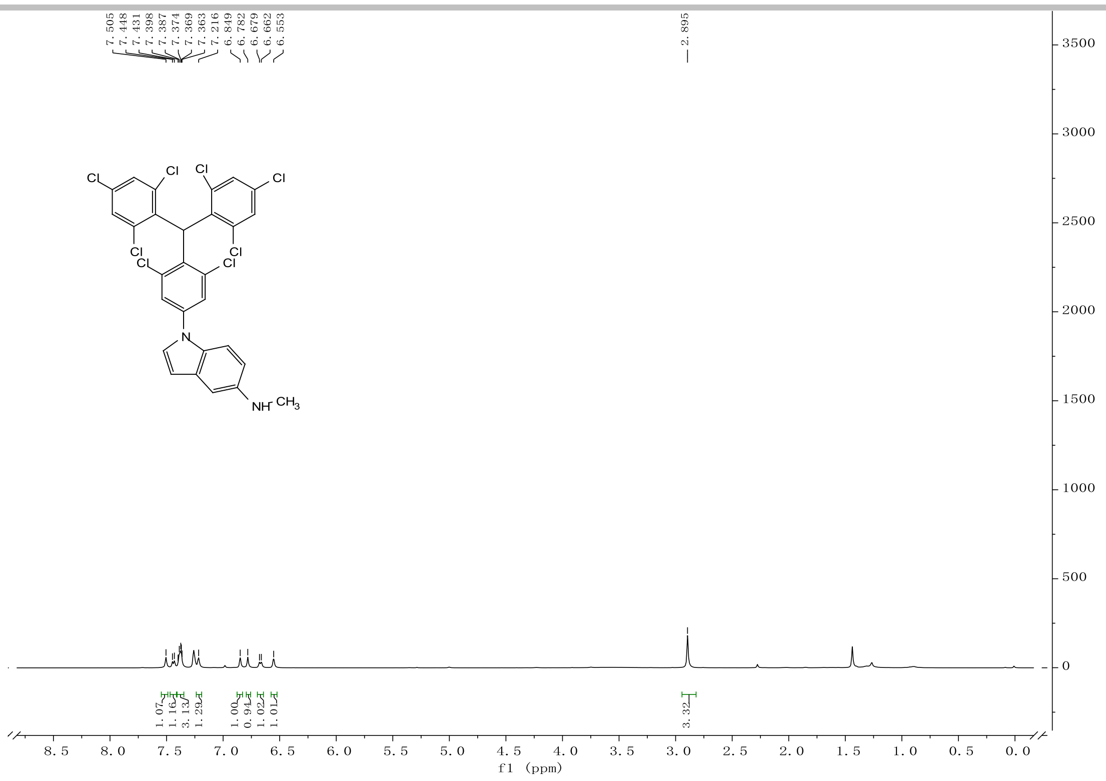

Figure S36. ^{1}H NMR (400 MHz, $CDCl_3$) of **TTMIndoNHMe-1**.

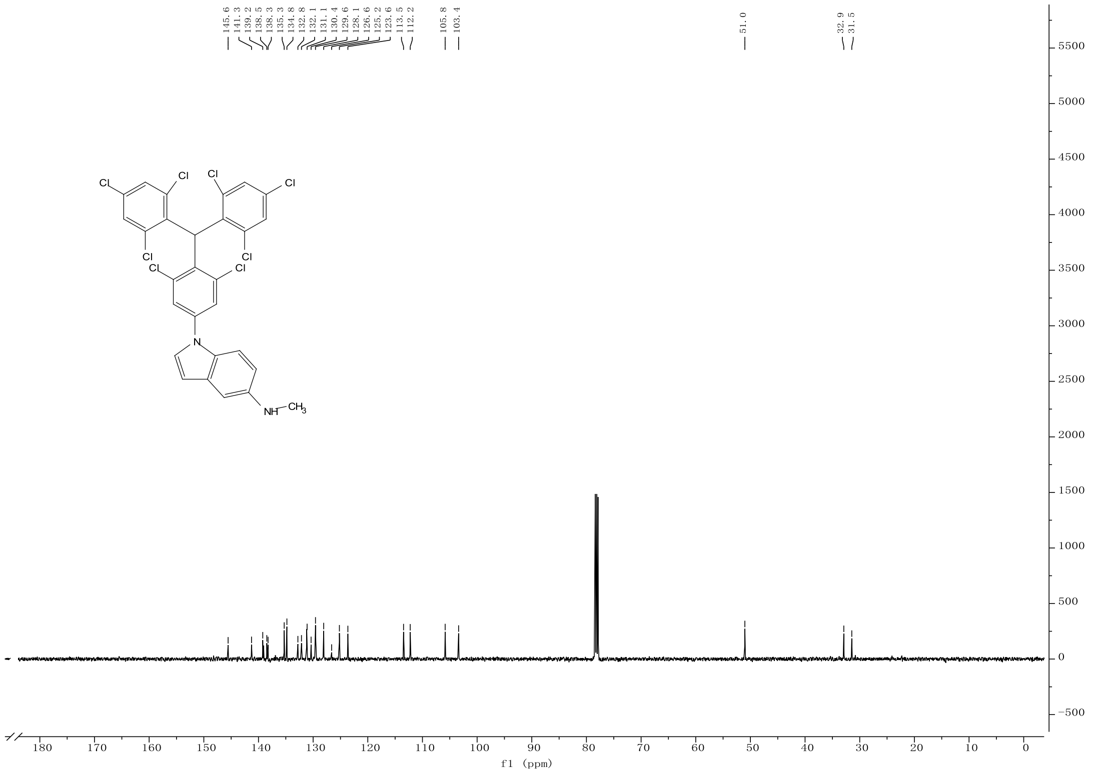

Figure S37. ^{13}C NMR (100 MHz, $CDCl_3$) of **TTMIndoNHMe-1**.

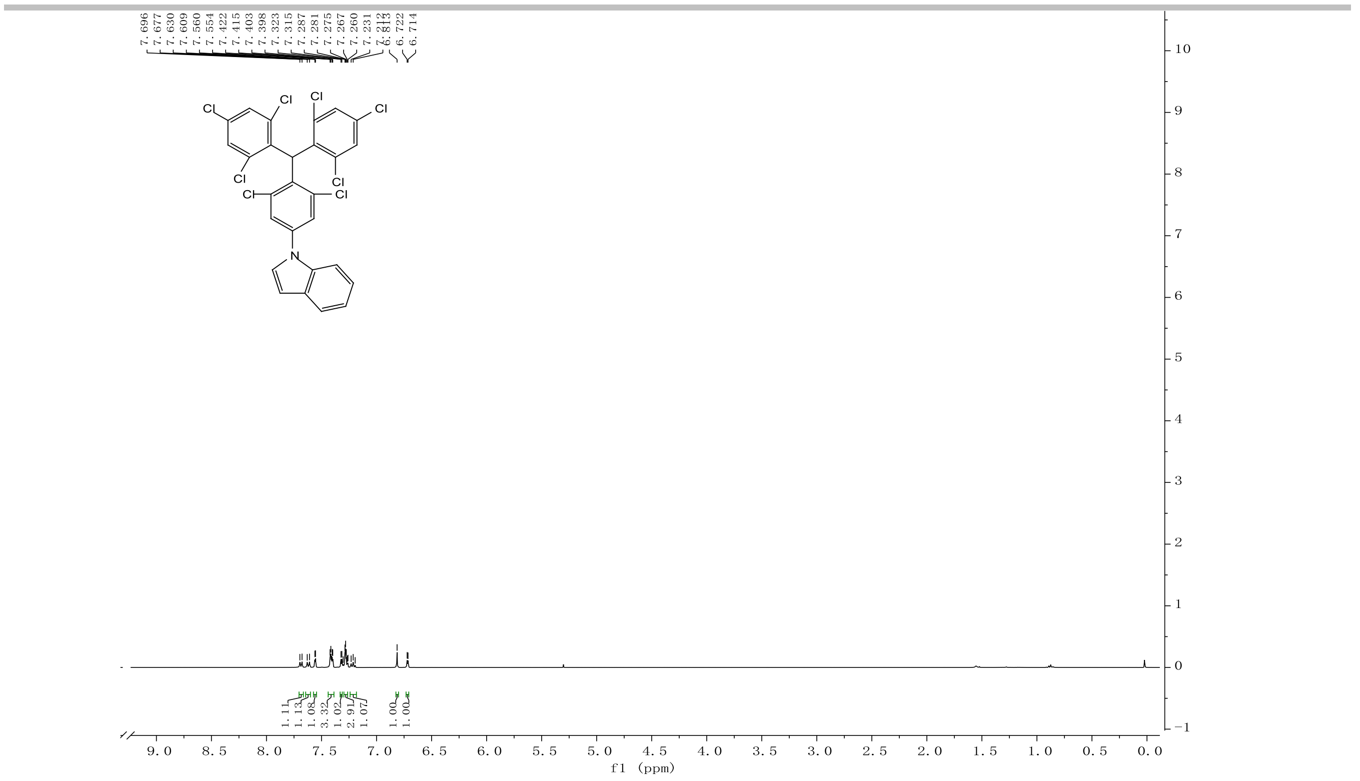

Figure S38. ^{1}H NMR (400 MHz, $CDCl_3$) of **TTMIndo-1**.

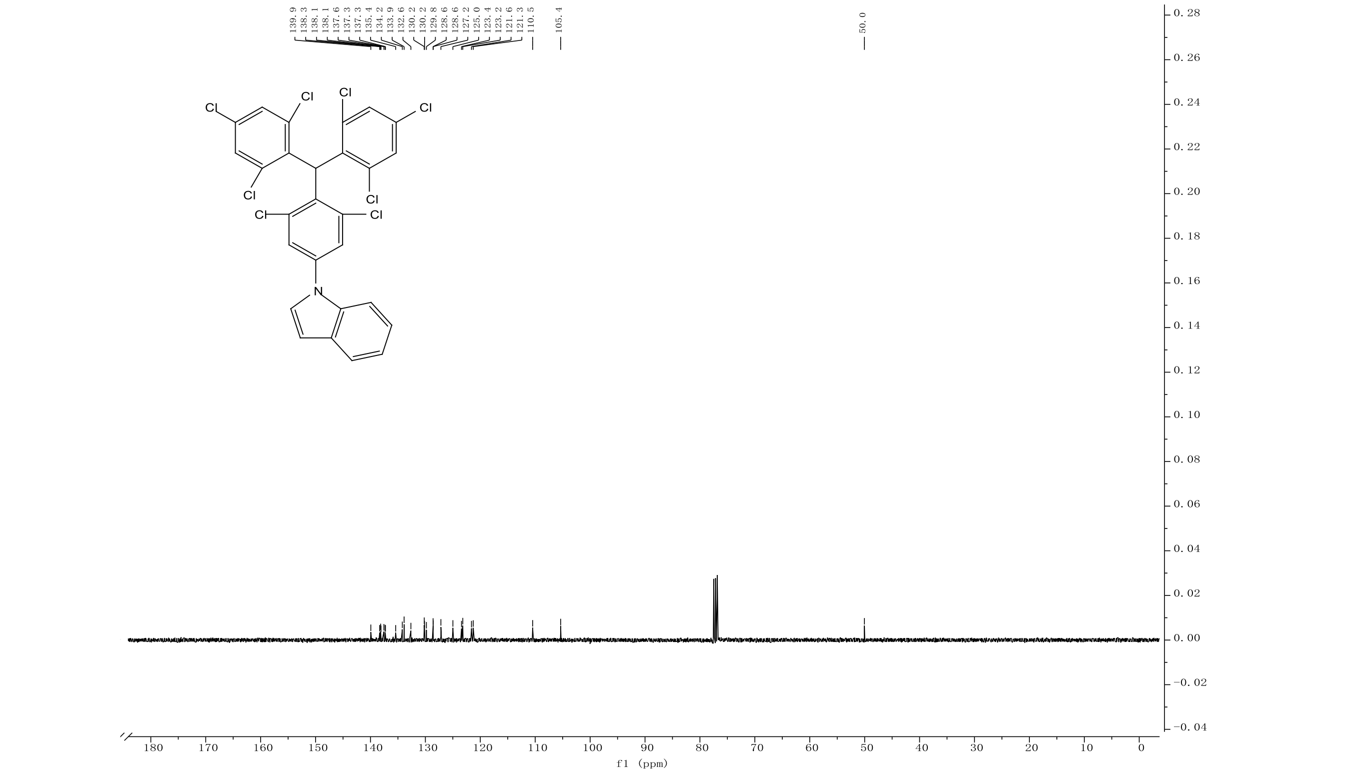

Figure S39. ^{13}C NMR (100 MHz, $CDCl_3$) of **TTMIndo-1**.

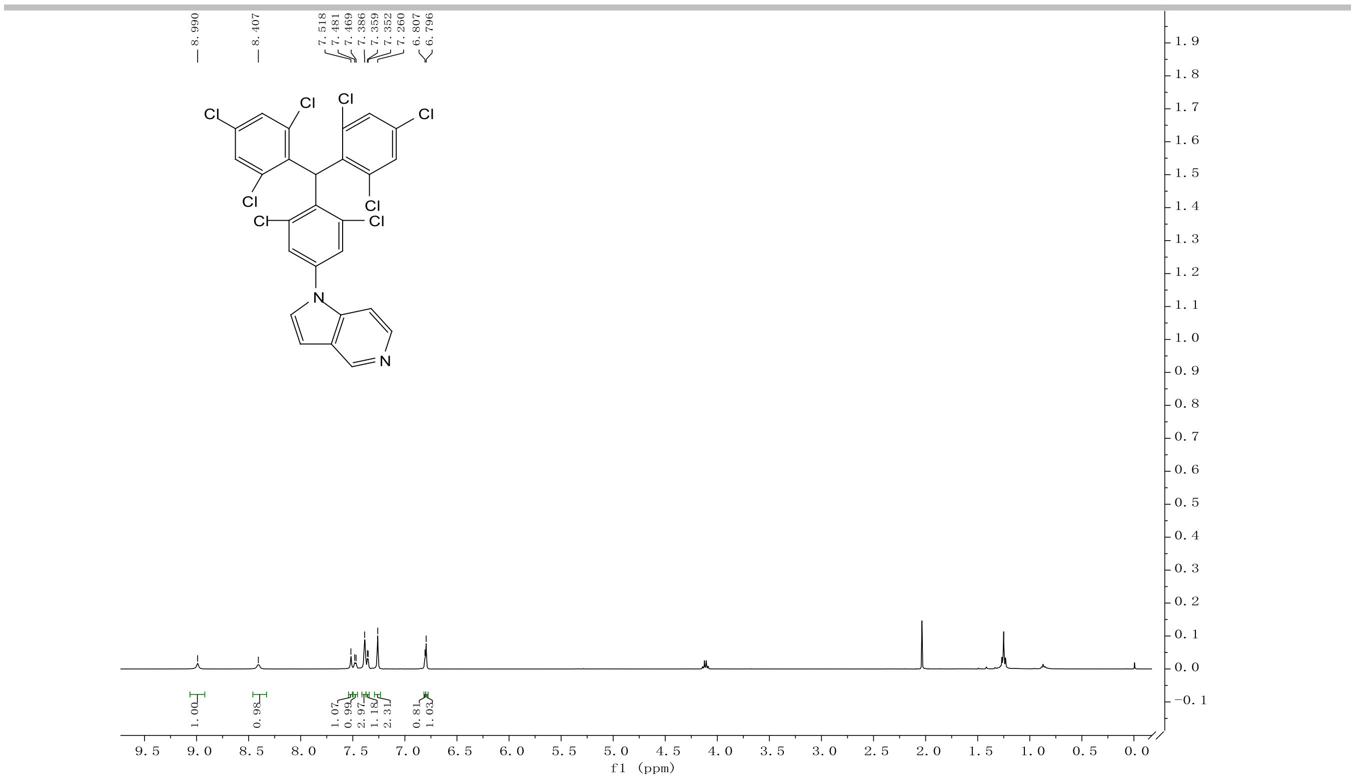

Figure S40. ^{1}H NMR (400 MHz, $CDCl_3$) of **TTMIndoN-1**.

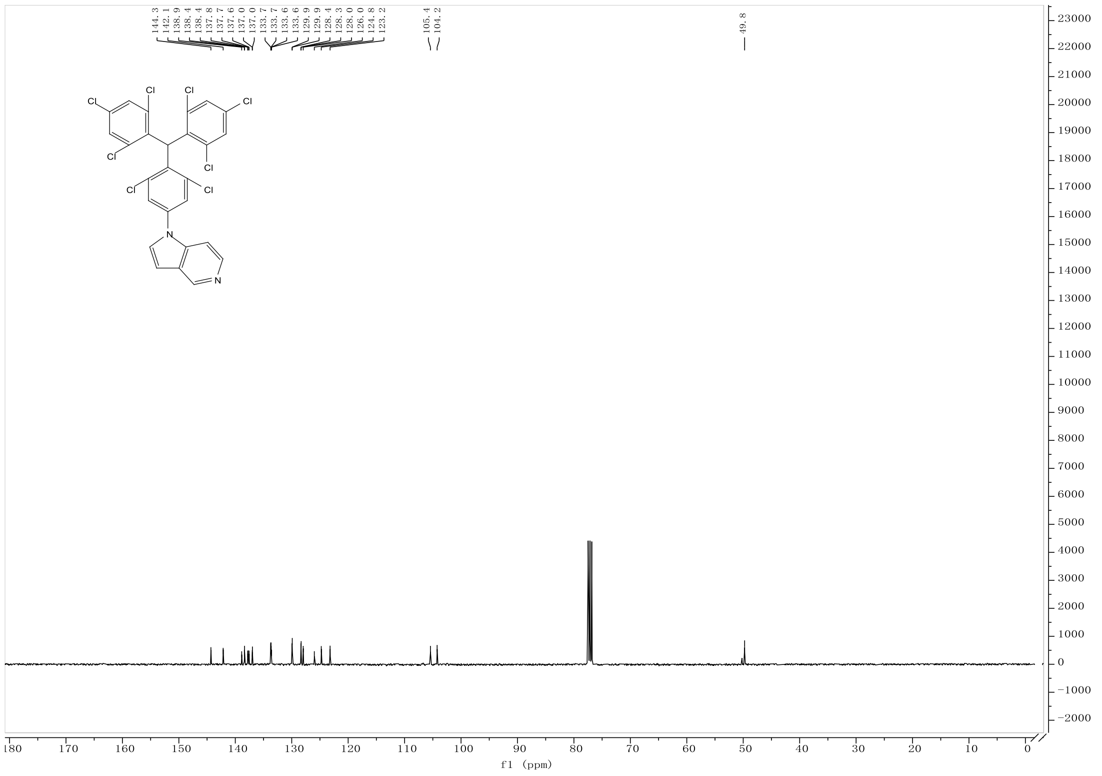

Figure S41. ^{13}C NMR (100 MHz, $CDCl_3$) of **TTMIndo-1**.

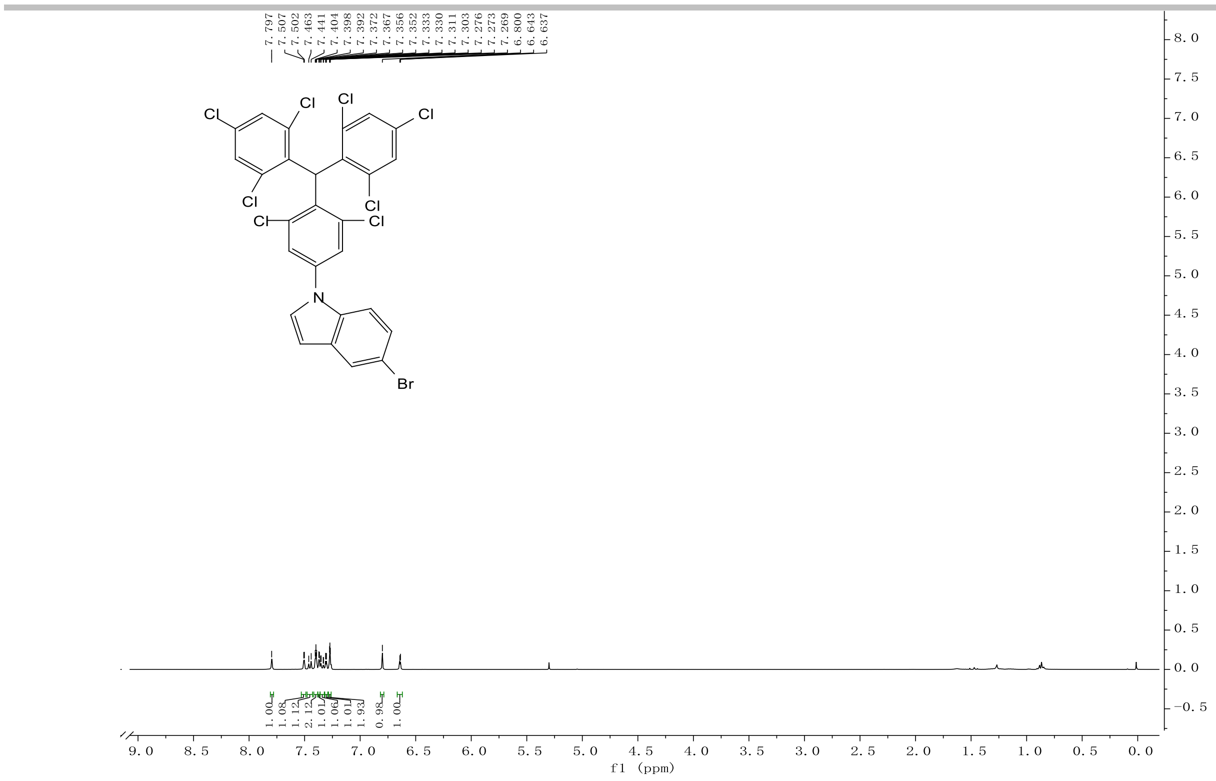

Figure S42. ^{1}H NMR (400 MHz, $CDCl_3$) of **TTMIndoBr-1**.

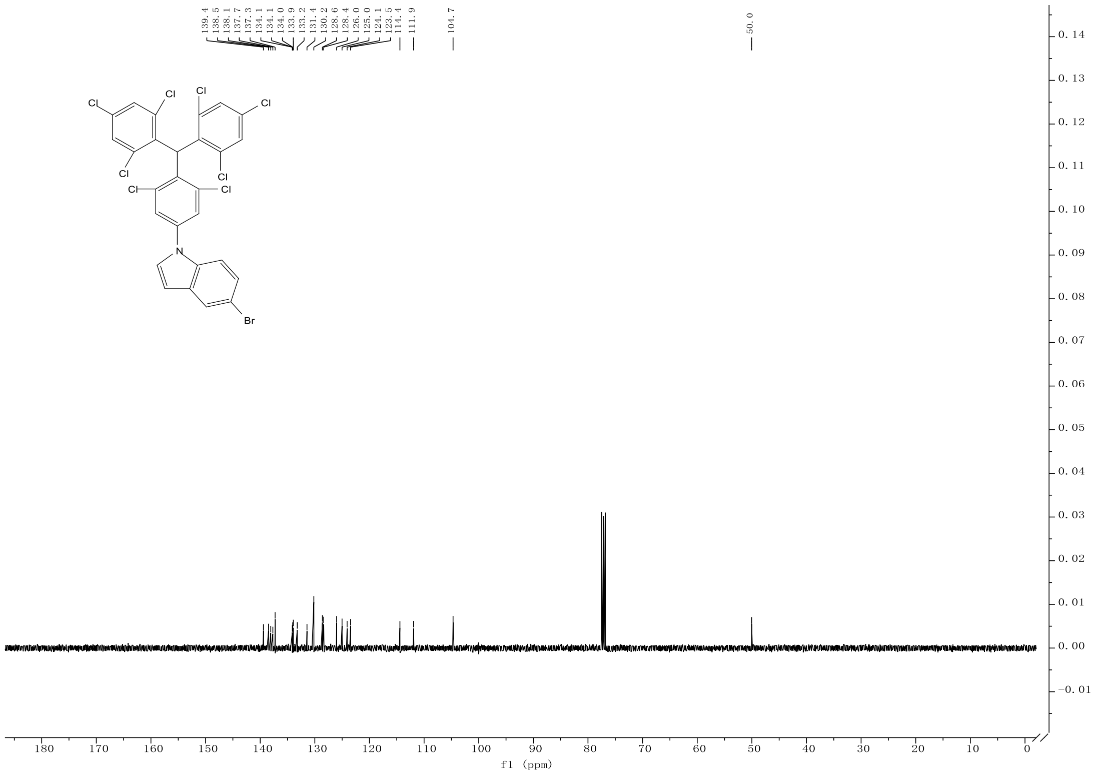

Figure S43. ^{13}C NMR (100 MHz, $CDCl_3$) of **TTMIndoBr-1**.

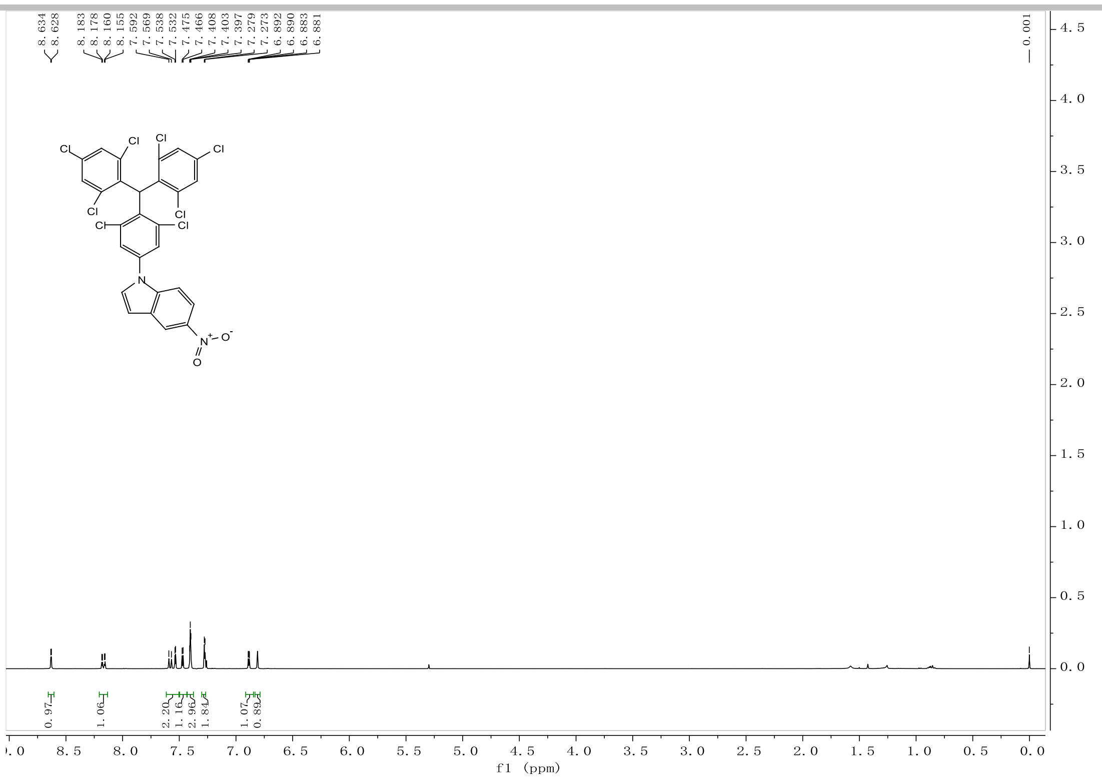

Figure S44. ^{1}H NMR (400 MHz, $CDCl_3$) of **TTMIndoNO$_2$-1**.

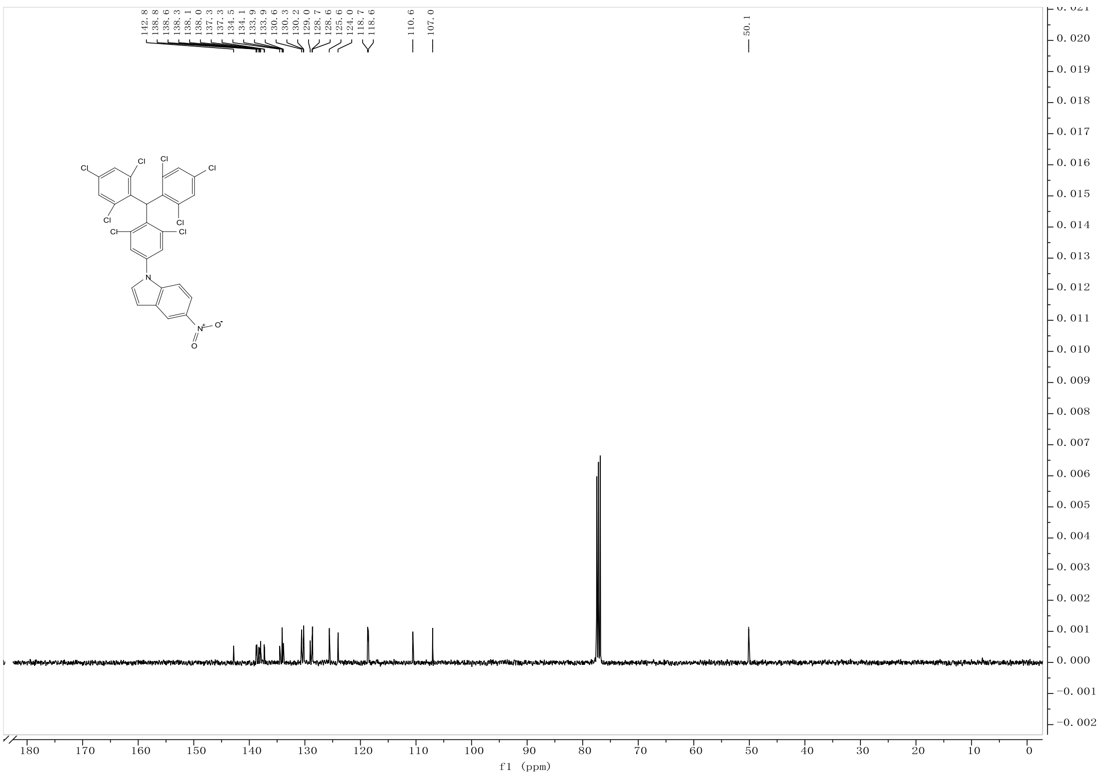

Figure S45. ^{13}C NMR (100 MHz, $CDCl_3$) of **TMIndoNO$_2$-1**.

References

[1] Gaussian 16, Revision C.01, M. J. Frisch, G. W. Trucks, H. B. Schlegel, G. E. Scuseria, M. A. Robb, J. R. Cheeseman, G. Scalmani, V. Barone, G. A. Petersson, H. Nakatsuji, X. Li, M. Caricato, A. V. Marenich, J. Bloino, B. G. Janesko, R. Gomperts, B. Mennucci, H. P. Hratchian, J. V. Ortiz, A. F. Izmaylov, J. L. Sonnenberg, D. Williams-Young, F. Ding, F. Lipparini, F. Egidi, J. Goings, B. Peng, A. Petrone, T. Henderson, D. Ranasinghe, V. G. Zakrzewski, J. Gao, N. Rega, G. Zheng, W. Liang, M. Hada, M. Ehara, K. Toyota, R. Fukuda, J. Hasegawa, M. Ishida, T. Nakajima, Y. Honda, O. Kitao, H. Nakai, T. Vreven, K. Throssell, J. A. Montgomery, Jr., J. E. Peralta, F. Ogliaro, M. J. Bearpark, J. J. Heyd, E. N. Brothers, K. N. Kudin, V. N. Staroverov, T. A. Keith, R. Kobayashi, J. Normand, K. Raghavachari, A. P. Rendell, J. C. Burant, S. S. Iyengar, J. Tomasi, M. Cossi, J. M. Millam, M. Klene, C. Adamo, R. Cammi, J. W. Ochterski, R. L. Martin, K. Morokuma, O. Farkas, J. B. Foresman, and D. J. Fox, Gaussian, Inc., Wallingford CT, **2019**.

[2] Lu, T.; Chen, F. Multiwfn: A multifunctional wavefunction analyzer. '*J. Comput. Chem.* **2012**, *33*, 580-592.

[3] Shi, G.; Zhang, C. N.; Xu, R.; Niu, J. F.; Song, H.; Zhang, X. Y.; Wang, W. W.; Wang, Y. M.; Li, C.; Wei, X. Q.; Kong, D. L. Enhanced antitumor immunity by targeting dendritic cells with tumor cell lysate-loaded chitosan nanoparticles vaccine. *Biomaterials* **2017**, *113*, 191-202.

[4] Shi, G.; Zhou, Y.; Liu, W.; Chen, C.; Wei, Y.; Yan, X.; Wu, L.; Wang, W.; Sun, L.; Zhang, T. Bone-derived MSCs encapsulated in alginate hydrogel prevent collagen-induced arthritis in mice through the activation of adenosine A2A/2B receptors in tolerogenic dendritic cells. *Acta Pharm Sin B* **2023**, *13*, 2778-2794.
